# Cobalt(I)-Catalyzed (3 + 2 + 2) Cycloaddition between Alkylidenecyclopropanes, Alkynes, and Alkenes

**DOI:** 10.1021/acs.orglett.3c03511

**Published:** 2023-11-10

**Authors:** Eduardo Da Concepción, Carlos Lázaro-Milla, Israel Fernández, José L. Mascareñas, Fernando López

**Affiliations:** † Centro Singular de Investigación en Química Biolóxica e Materiais Moleculares (CiQUS) and Departamento de Química Orgánica, Universidade de Santiago de Compostela, 15782, Santiago de Compostela, Spain; § Departamento de Química Orgánica I, Facultad de Ciencias Químicas, Universidad Complutense de Madrid, 28040 Madrid, Spain; ‡ Misión Biológica de Galicia, Consejo Superior de Investigaciones Científicas (CSIC), 36080 Pontevedra, Spain

## Abstract

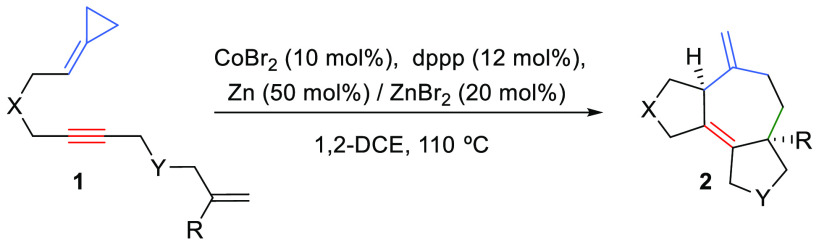

Cobalt(I) catalysts equipped with bisphosphine ligands can be used to promote formal (3 + 2 + 2) intramolecular cycloadditions of enynylidenecyclopropanes of type **1**. The method provides synthetically appealing 5,7,5-fused tricyclic systems in good yields and with complete diastereo- and chemoselectivity. Interestingly, its scope differs from that of previously reported annulations based on precious metal catalysts, specifically rhodium and palladium. Noticeably, density functional theory calculations confirm that the mechanism of the reaction is also different from those proposed for these other catalysts.

Metal-catalyzed cycloadditions can be catalogued among the most practical reactions to build cyclic products from acyclic precursors.^1^ While most formal cycloadditions involve two-carbon (2C) unsaturated substrates, a number of annulations involving 3C precursors have also been described.^2^ Specifically, alkylidenecyclopropanes (ACPs) are highly versatile 3C synthons that can participate in a variety of metal-catalyzed (3 + *n*) formal cycloadditions with different types of unsaturated partners.^3^

Most reports on this type of annulation are based on the use of noble metal-based catalysts, especially Pd and Rh, and are initiated by the distal cleavage of the ring to give metallacyclobutanes of type **A** (Scheme 1, eq 1).^4^ Migratory insertion of the unsaturated partner gives six-membered metallacycles that evolve to the product (**2**) by reductive elimination. Interestingly, it is possible to add an extra unsaturated 2C component to the reaction and, therefore, carry out formal (3 + 2 + 2) intramolecular cycloadditions (Scheme 1, eq 2).^5^ These types of tandem annulations provide a direct entry to polycyclic structures containing seven-membered carbocycles, a type of scaffold that forms the core of many types of bioactive natural products.^6^

**Scheme 1:**
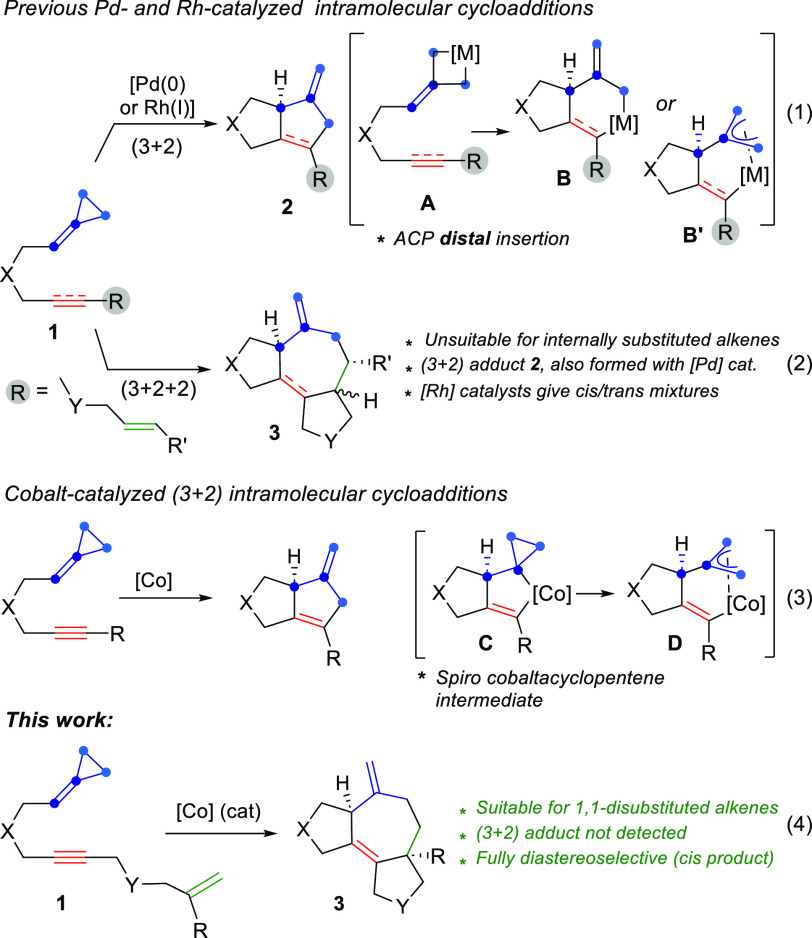
Previously Developed Related Intramolecular Cycloadditions of ACPs and This Work

Recently, our group and the Yu group independently found that cobalt(I) complexes can promote (3 + 2) cycloadditions of ynilidenecyclopropanes (Scheme 1, eq 3).^7^ This transformation is very attractive not only because it uses more sustainable, less expensive transition metals but also because its mechanism does not entail the standard pathway based on the insertion of the metal into the distal bond of the cyclopropyl ring. Instead, density functional theory (DFT) calculations supported an alternative path involving a spiro metallacyclic intermediate of type **C**. This species evolves by cyclopropane opening to a highly stable π-allyl cobaltacyclic species **D**, which undergoes a relatively easy reductive elimination, significantly less demanding than that of related intermediates formed with Rh catalysts (ca. ΔΔ*G*^‡^ ∼ 9 kcal/mol).^7a^

Considering the increasing socioeconomic pressure to develop more sustainable synthetic processes, and the challenges associated with using first-row transition metals in catalytic processes,^8,9^ we questioned whether cobalt catalysts could be used to promote (3 + 2 + 2) cycloadditions. At the outset, this was not trivial because the competing reductive elimination to give (3 + 2) cyclopentene adducts could be especially favored with Co catalysts.^7^ Herein we demonstrate that this Co-catalyzed multicomponent (3 + 2 + 2) annulation is indeed feasible and that ACPs of type **1** can be readily converted into *cis*-fused 5,7,5-polycyclic adducts **3** in a fully diastereoselective manner (Scheme 1, eq 4). The reactions are promoted by low-valent Co catalysts generated from Co(II) precursors and a reductant and hold a different but complementary scope than that provided by Pd and Rh catalysts. Indeed, the *trans* cycloadducts (**3′**), which are formed with Rh catalysts,^5d^ are not detected in the cobalt chemistry. Moreover, the formation of competitive (3 + 2) adducts (**2**), typical with Pd catalysts,^5a^ can be avoided by appropriate tuning of the Co catalyst. As will be shown below, DFT calculations confirmed that the mechanistic scenario for cobalt is different from that proposed for these other noble metals.

We started the study by analyzing the viability of the (3 + 2 + 2) intramolecular reaction using the model substrate **1a**, which bears a 1,6-diyne moiety tethered to the ACP (Scheme 2). In addition to the desired 5,7,5-tricyclic product **3a**, the reaction could provide competitive (3 + 2) and/or (2 + 2 + 2) adducts of type **2** and **4**. Unfortunately, the *in situ* generated low-valent cobalt catalyst, previously shown to be efficient for the (3 + 2) cycloaddition of ACPs and alkynes,^7a^ failed to give the desired adduct **3a**, or any alternative products, so that **1a** was mostly recovered after heating at 100 °C for 16 h.^10^ Exploration of alternative conditions or Co catalysts was also unsuccessful.

**Scheme 2:**
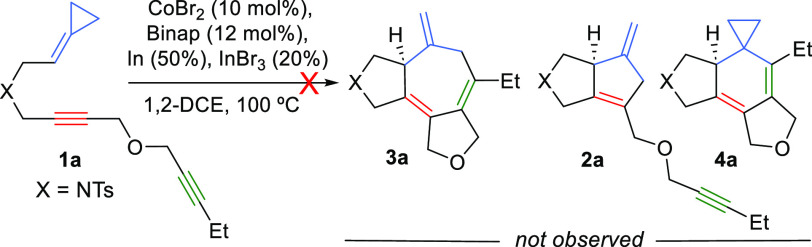
Preliminary Tests Using Diyne **1a**

We envisioned that this lack of reactivity could be associated with the well-known ability of cobalt complexes to strongly coordinate 1,*n*-diynes^8c,11^ and therefore we assayed the reactivity of the analogue **1b**, which bears a terminal alkene instead of an alkyne as third cycloaddition partner. When this precursor was treated with the catalyst generated *in situ* from CoBr_2_, Binap, and a reducing agent (In/InBr_3_) at 110 °C in 1,2-DCE, we observed reactivity but, unfortunately, the product turned out to be the (3 + 2) adduct **2b**, which was isolated in a 73% yield (Table 1, entry 1). Modifying the reaction temperature (entry 2) or the solvent (entry 3) did not change the chemoselectivity of the process. However, we were glad to observe that, using dppp instead of Binap, the desired (3 + 2 + 2) adduct **3b** could be obtained as a single product in a 51% yield (entry 4). This product was also the only cycloadduct detected when using other solvents or at lower temperatures (entries 5 and 6). Thus, NMR analysis of the crude mixture indicated that neither the (3 + 2) adduct **2b** nor alternative (2 + 2+2) products were formed under these conditions. Likewise, NMR confirmed that **3b** is a single stereoisomer, with the hydrogen atoms at the rings fusion in *cis* disposition. Importantly, the efficiency of the process to give adduct **3b** could be slightly increased by using Zn/ZnBr_2_ instead of In/InBr_3_ under otherwise identical reaction conditions (entry 7, 65% yield).^12^

**Table 1:**
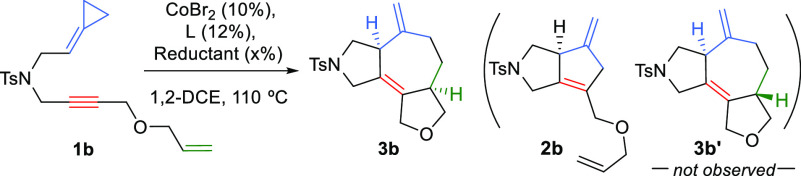
Preliminary Optimization of the Co-Catalyzed (3 + 2 + 2) Cycloaddition with the ACP Precursor **1b**^a^

entry	**L**	reductant (mol %)	product, yield
1	Binap	In (50)/InBr_3_ (20)	**2b**, 73%
2^b^	Binap	In (50)/InBr_3_ (20)	**2b**, 53%
3^c^	Binap	In (50)/InBr_3_ (20)	**2b**, 77%
4	dppp	In (50)/InBr_3_ (20)	**3b**, 51%
5^c^	dppp	In (50)/InBr_3_ (20)	**3b**, 47%
6^b^	dppp	In (50)/InBr_3_ (20)	**3b**, 50%
7	dppp	Zn (50)/ZnBr_2_ (20)	**3b**, 65%

a Conditions: **1b**, CoBr_2_ (10 mol %), **L** (12 mol %), and reductant [Zn or In (50 mol %)/ZnBr_2_ or InBr_3_ (20 mol %)] were heated in 1,2-DCE for 16 h at 110 °C. Full conversions were observed.

b Carried out at 80 °C.

c Carried out in acetonitrile.

Having developed an optimal protocol for the (3 + 2 + 2) cycloaddition reaction, we analyzed the scope of the process. Thus, a series of precursors bearing different connecting tethers between the ACP, alkyne, and alkene partners were synthesized. Gratifyingly, as can be deduced from Scheme 3, substrates in which the alkene is connected to the alkyne via a carbonade chain do also participate in the annulation; therefore, products like **3c** and **3d** were obtained in good yields and, again, with complete selectivity toward the *cis* isomer. Likewise, a precursor that bears a carbon-based tether between the ACP and the alkyne provided a 73% yield of the desired seven-membered cycloadduct **3e**. Furthermore, a fully carbon-based precursor also reacted to give **3f** in a 77% yield. The use of precursors bearing nitrogen- and oxygen-based tethers was equally possible, so that in all cases the formation of the tricyclic products (**3g**–**3j**) was observed in moderate to good yields. Then, we checked the influence of the alkene substitution in the multicomponent cycloaddition reaction. Curiously, and contrary to the results obtained with Pd and Rh catalysts,^5a,5d^ the cobalt-catalyzed reaction of these precursors led to the exclusive formation of the (3 + 2) adducts **2k** and **2l**, which were isolated in good yields.^10^ However, the cobalt-based technology was effective for the formation of tricyclic products such as **3m** and **3n** bearing carbon quaternary centers at the ring fusion, a type of product that was not accessible using the Rh and Pd-based methodologies. Indeed, treatment of the cycloaddition precursor **1m** with the reported Rh catalysts provided a complex mixture of unidentified products,^5d^ and the use of a Pd reagent led to the exclusive formation of a (3 + 2) adduct (**2m**, 72% yield). Additionally, the presence of an internal substituent at the double bond of the ACP is also tolerated in the cobalt chemistry. Thus, interesting cycloadducts such as **3o** could be readily built using the standard reaction conditions.

**Scheme 3:**
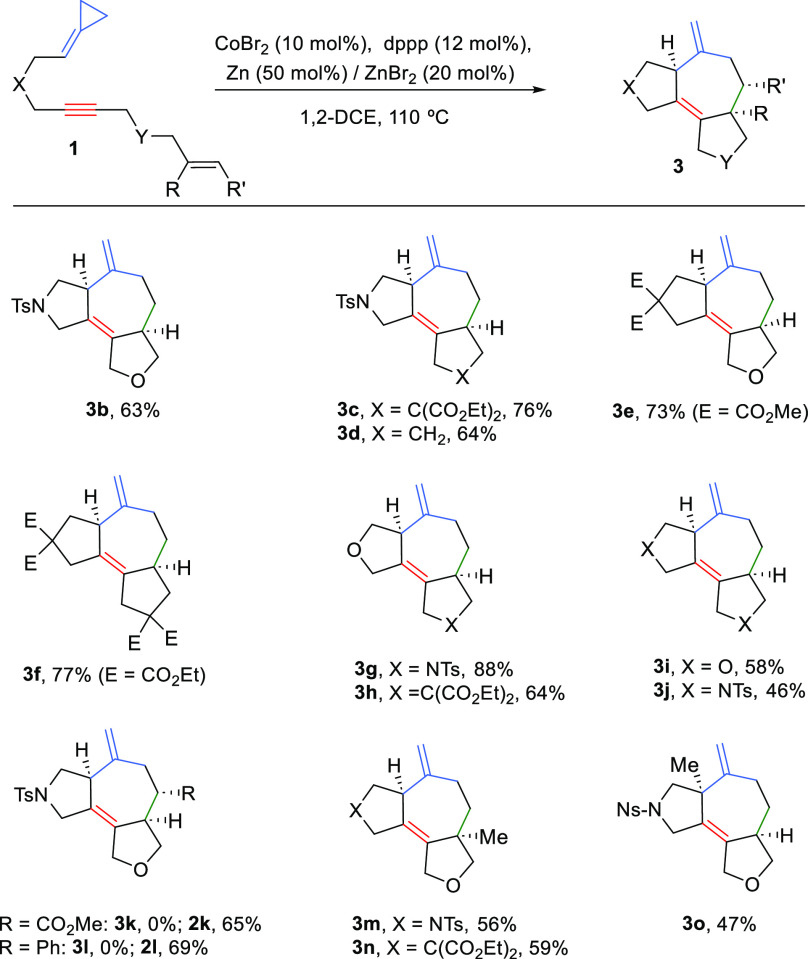
Scope of the Co-Catalyzed (3 + 2 + 2) Annulation Conditions: **1**, CoBr_2_ (10 mol %), dppp (12 mol %), and Zn (50 mol %)/ZnBr_2_ (20 mol %) were heated in DCE at 110 °C for 16 h.

The synthetic potential of the cycloadducts was preliminarily investigated. As can be seen in Scheme 4, treatment of **3b** with K_2_OsO_4_ and subsequent cleavage of the resulting diol with NaIO_4_ led to the tricyclic ketone **5b** in a 63% yield, without affecting the integrity of its adjacent stereocenter. On the other hand, reaction of **3b** with H_2_, in the presence of Pd/C, led to the exclusive hydrogenation of the *exo* methylene moiety to afford **6b** as a single diastereoisomer.

**Scheme 4:**
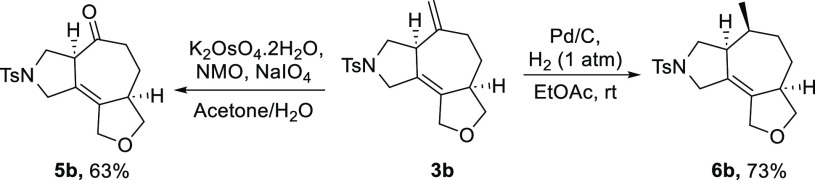
Synthetic Manipulation of the Cycloadducts

The data presented above indicate that the steric requirements of the Co-catalyzed annulation differ from those of Pd- and Rh-catalyzed reactions. Indeed, the scope of the reactions with different metal catalysts is rather complementary, which is interesting from a synthetic perspective. These differences suggest that the underlying mechanisms of the cobalt-promoted annulation might be different.

To obtain mechanistic information, as well as to shed light on the reasons for the distinct reactivity observed with the cobalt catalysts, we carried out DFT calculations at the dispersion-corrected PCM(DCE)-B3LYP-D3/def2-SVP level.^13^ We used the ACP precursor **1p** as the model substrate and the cationic Co(I) complex [Co(dppp)]^+^ as the reference catalyst (Figure 1). Similar to the (3 + 2) cycloadditions of alkynylidenecyclopropanes,^7a^ the process begins with the highly exergonic formation of **INT0**, an intermediate in which the alkyne and the C=C double bond of the ACP are coordinated to [Co(dppp)]^+^ (Figure 1). However, the presence of a second alkene in **1p** enables an alternative cobaltenyne complex, **INT0′**, whose formation was indeed found to be energetically comparable to that of **INT0** (−48.4 vs −47.3 kcal/mol, Figure 1). From these Co(I) complexes, we evaluated two possible oxidative cyclization processes to provide, respectively, the spiro cobaltacyclopentene intermediate **INT1** and its counterpart **INT1′**. Although both reactions require a rather similar energy barrier (Δ*G*^‡^ = 14.1 and 15.0 kcal/mol), the cobaltacyclopentene species **INT1** is significantly more stable than **INT1′** by 8.4 kcal/mol. Thus, intermediate **INT1** would then evolve via a cyclopropyl to π-allyl rearrangement toward the intermediate **INT2**, which is more than 20 kcal/mol more stable than **INT1**, through transition state **TS2** (Δ*G*^‡^ = 26.9 kcal/mol). At this point, a C–C reductive elimination through **TS3** would deliver the (3 + 2) adduct, a process that conveys an energy barrier of 20.1 kcal/mol. Gratifyingly, our calculations suggest that the alternative coordination of the alkene to provide **INT4**, followed by an almost barrierless migratory insertion to the Co(III) center via **TS4**, occurs with an overall lower energy barrier of only 17.8 kcal/mol (with respect to **INT2**). Therefore, the pathway eventually leading to the (3 + 2 + 2) adduct is favored by 2.3 kcal/mol, which is consistent with the experimental observations. From **INT5**, a C–C reductive elimination via **TS5** (Δ*G*^‡^ = 21.5 kcal/mol) delivers **INT6**, which upon decoordination of the cobalt catalyst affords the product.

**Figure 1:**
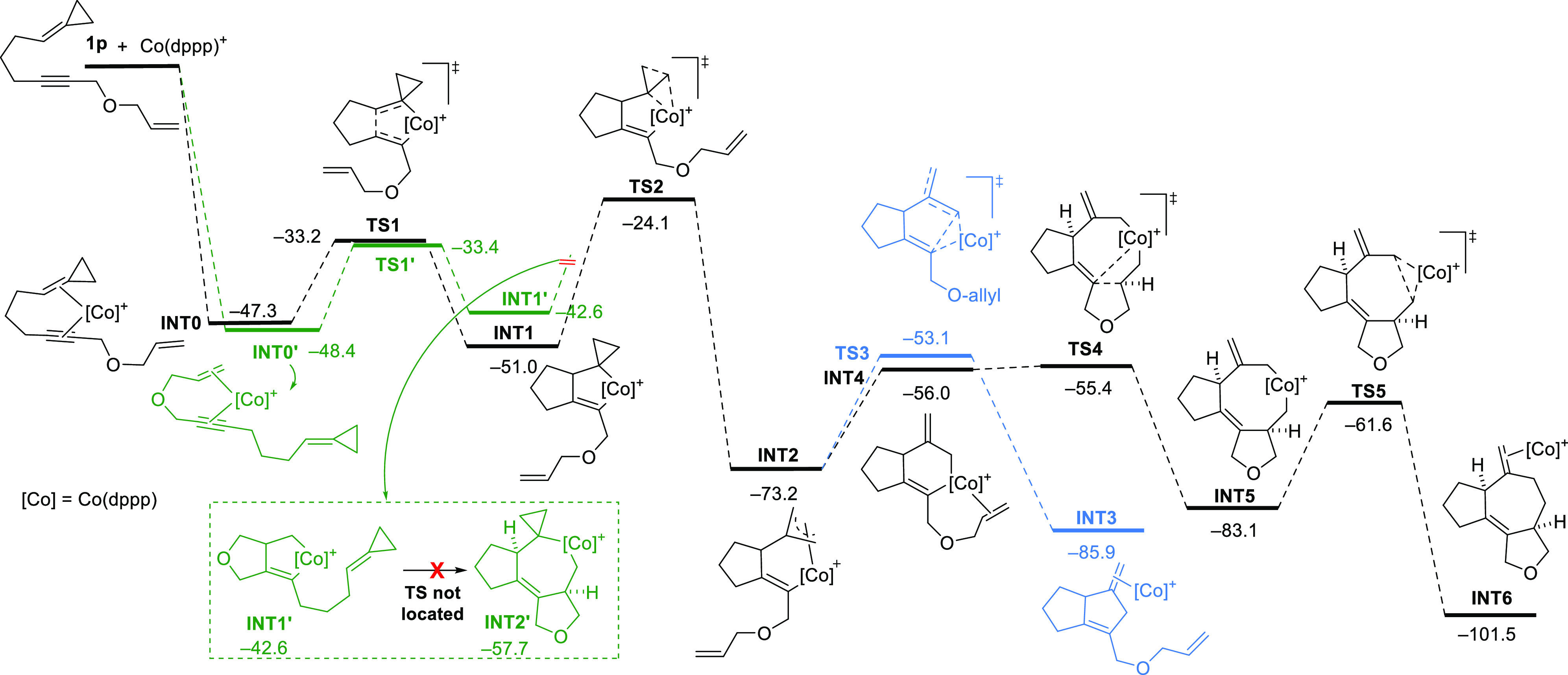
Computed reaction profile for reaction model of ACP **1p** and [Co(dppp)]^+^. Relative free energies (Δ*G*, at 298 K) are given in kcal/mol. All data were computed at the PCM(DCE)-B3LYP-D3/def2-SVP level.

Regarding **INT1′**, we also studied the coordination and migratory insertion of the ACP unit. However, all attempts to locate the corresponding transition state for this step were unfruitful (from **INT1′** to **INT2′**, see Figure 1 inlet and Figure S2), suggesting that the presence of the fused-cyclopropyl ring hinders the migratory insertion step. In any case, the evaluation of a cyclopropyl-to π-allyl rearrangement from a hypothetical intermediate **INT2′**, to yield the corresponding π-allyl intermediate **INT5′** led to an unfeasible energy barrier of more than 40 kcal/mol (**TS3′**, Figure S2), which makes this alternative very unlikely.^10^ Therefore, the tandem annulation promoted by the cobalt catalyst proceeds through a key spiro cobaltacyclopentene intermediate **INT1** followed by a rearrangement to the π-allyl isomer **INT2**. Very likely, the shorter M–C distances when M is Co instead of Rh render the subsequent migratory insertion more sensitive to the presence of substituents at the terminal position of the alkene, while internal substitutions are tolerated.

Indeed, the energy barrier of the alkene migratory insertion calculated for a precursor that bears a methyl substituent at the internal position (analog to **1m**, Scheme 3) is essentially identical, and the subsequent reductive elimination is not affected (Figure S3). This would explain the different scopes with respect to the substitution at the alkene partner, depending on the transition metal used.

In summary, we have developed the first cobalt-catalyzed (3 + 2 + 2) formal cycloaddition of ACPs. The reaction is promoted by a low-valent cobalt catalyst generated in situ from CoBr_2_, a bisphosphine ligand and a reducing agent, is highly chemo- and diastereoselective, and presents a rather good scope, complementary to that previously described with precious metals like Rh or Pd. Therefore, contrary to Pd catalysts, which frequently afford mixtures of (3 + 2) and (3 + 2 + 2) cycloadducts,^5a^ or to Rh catalysts, which tend to afford mixtures of (3 + 2 + 2) stereoisomers,^5d^ the cobalt catalyst is fully selective toward a single seven-membered stereoisomer (**3**). DFT calculations supported a mechanistic scenario that differs from those proposed for Pd and Rh complexes, based on the key formation of a spiro cobaltacyclopentene intermediate. Our results reinforce the promise of first-row, earth-abundant metals as catalysts for annulation reactions and unveil new reactivity paths with ACPs.

## Data Availability

The data underlying this study are available in the published article and its Supporting Information.

## Supporting Information Available

Full experimental procedures, optimization of the catalyst, and characterization of all new compounds, including ^1^H and ^13^C NMR spectra (PDF)
