# Supplementary material for: Cobalt(I)-Catalyzed (3 + 2 + 2) Cycloaddition between Alkylidenecyclopropanes, Alkynes, and Alkenes
Source: Org Lett. 2023 Nov 10;25(46):8372–6. doi: 10.1021/acs.orglett.3c03511 (PMC10723761; doi:10.1021/acs.orglett.3c03511)
Supplement: Supplementary file 1 — ol3c03511_si_001.pdf [file ol3c03511_si_001.pdf]

SUPPORTING INFORMATION

Cobalt (I)-catalyzed (3+2+2) Cycloaddition between Alkylidenecyclopropanes, Alkynes and Alkenes

Eduardo Da Concepción,[†] Carlos Lázaro-Milla,[†,§] Israel Fernández,[§] José L. Mascareñas,*[†] Fernando López*[‡,†]

† Centro Singular de Investigación en Química Biolóxica e Materiais Moleculares (CiQUS) and Departamento de Química Orgánica. Universidade de Santiago de Compostela, 15782, Santiago de Compostela, Spain

§ Departamento de Química Orgánica I, Facultad de Ciencias Químicas, Universidad Complutense de Madrid, 28040, Madrid, Spain

‡ Misión Biológica de Galicia, Consejo Superior de Investigaciones Científicas (CSIC), 36080, Pontevedra, Spain

Email: joseluis.mascarenas@usc.es

Email: fernando.lopez@csic.es

Table of contents

1. General Procedures S3
2. Synthesis of ACP cycloaddition precursors
   General procedure A: alkylation approach
   General procedure B: assembly through Mitsunobu reaction
   General procedure C: assembly through Tsuji-Trost reaction
   General procedure D: assembly through tosylate substitution S4
3. Preliminary investigations with ACP precursors **1a** and **1q** S11
4. General procedure for the intramolecular (3+2+2) cycloaddition S12
5. Synthetic manipulations of cycloadduct S17
6. Computational details S19
7. Cartesian coordinates S20
8. NMR spectra S57
9. References S85

1. General Procedures

All reactions were conducted in dry solvents under Ar atmosphere unless otherwise stated. The abbreviation "rt" refers to reactions carried out at 20-25 °C. Reaction mixtures were stirred using Teflon-coated magnetic stir bars. Reaction temperatures were maintained using Thermowatch-controlled silicone oil baths. Dry solvents were obtained from a solvent purification system (Mbraun, SPS-5) or freshly distilled under Ar from an appropriate drying agent before use (toluene from Na / benzophenone, CH_2Cl_2 and Et_3N were distilled form CaH_2). Pd_2dba_3 and all other reagents for the synthesis of precursors were purchased from Aldrich. All the reagents used for the cycloaddition reaction were purchased from Aldrich with the exception of $CoBr_2$, that was purchased from Alfa Aesar. Previous to use, Zn powder was purified according to the literature. Thin-layer chromatography (TLC) was performed on silica gel plates and components were visualized by observation under UV light, or by treating the plates with either *p*-anisaldehyde, ninhydrin, potassium permanganate or cerium nitrate solutions, followed by heating. Flash chromatography was carried out on silica gel unless otherwise stated. Dryings were performed with anhydrous Na_2SO_4 or $MgSO_4$. Concentration refers to the removal of volatile solvents via distillation using a Buchi rotary evaporator, followed by residual solvent removal under high vacuum. In reactions carried out in sealed tubes, the mixture must be at rt before sealing, to avoid overpressure at high temperatures.

^{1}H and ^{13}C-NMR spectra were recorded in $CDCl_3$ at 300 MHz (Varian) for cycloaddition precursors and cycloadducts or Bruker 500 MHz for some cycloadducts. Carbon types were determined from DEPT-NMR experiments. NMR spectra were analysed using MestreNova© processing software (www.mestrelab.com). 1,3,5-Trimethoxybenzene was used as internal standard for analyzing the reaction crudes by NMR.

^{1}H-NMR spectral data are reported as follows: chemical shift (δ ppm), integration, multiplicity (s = singlet, d = doublet, t = triplet, q = quartet, dd = double doublet, td = triple doublet, m = multiplet, br = broad). Data for ^{13}C are reported in terms of chemical shift relative to the residual solvent peak. Mass spectra were acquired using chemical ionization (CI) or electron impact techniques (EI) and were recorded at the CACTUS facility of the USC. The structure of the cycloadducts was determined by standard 1D-NMR experiments in agreement with previous literature reports.

2. Synthesis of cycloaddition precursors

General procedure A: alkylation approach (illustrated for **1a**)

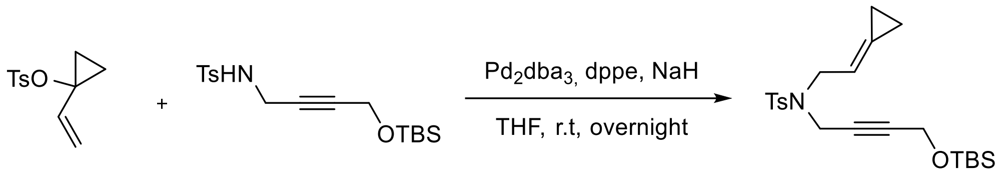

S1

N-(4-((tert-butyldimethylsilyl)oxy)but-2-yn-1-yl)-4-methylbenzenesulfonamide (4.75 mmol) was added slowly to a suspension of NaH (200 mg, 5.00 mmol) in THF (24 mL), cooled at 0°C. After stirring for 15 min, a solution of 1-vinylcyclopropyl 4-methylbenzenesulfonate (1.13 g, 4.75 mmol), $Pd_2(dba)_3$ (87.0 mg, 0.095 mmol) and dppe (76.0 mg, 0.19 mmol) in THF (24 mL), previously stirred for 20 min, was added "via cannula". The reaction mixture was stirred overnight at rt, poured into water, and extracted with Et_2O (3 x 20 mL). The organic phases were dried, filtered and concentrated to give a crude oily residue that was purified by flash chromatography (10 % Et_2O/hexanes) to yield **S₁** as pale-yellow oil (1.68 g, 84 % yield). Its NMR data is in accordance with that previously reported.[1] **^{1}H NMR (300 MHz, $CDCl_3$)** *δ* (ppm): 7.74 (d, *J* = 8.4 Hz, 2H), 7.28 (d, *J* = 8.5 Hz, 2H), 5.70 (t, *J* = 1.9 Hz, 1H), 4.09 (s, 2H), 4.03 (s, 2H), 3.95 (d, J = 6.9 Hz, 2H), 2.42 (s, 3H), 0.86 (s, 9H), 0.03 (s, 6H).

***N*-(2-Cyclopropylideneethyl)-4-methyl-N-(4-(pent-2-yn-1-yloxy)but-2-yn-1-yl)benzenesulfonamide (1a)**

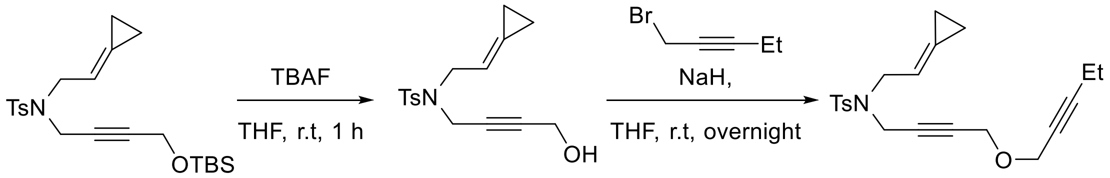

S1 **S2** **1a**

To a solution of **S_1** (2.8 g, 6.7 mmol) in THF (33 ml) was slowly added TBAF (7.3 ml, 1.0 M in THF). The reaction mixture was stirred at rt until full consumption of **S_1** (1 h) was observed by TLC. The reaction was quenched with water, extracted with Et_2O (3x) and concentrated under vacuum. Finally, flash column chromatography (10% EtOAc/hexanes) afforded **S_2** as a yellowish oil (2.0 g, 75% yield). Its NMR data is in accordance with that previously reported.[2] **^{1}H NMR (300 MHz, $CDCl_3$)** *δ* (ppm): 7.68 (d, *J* = 8.3 Hz, 2H), 7.26 (d, *J* = 8.0 Hz, 2H), 5.62 (ddt, *J* = 6.9, 4.3, 2.2 Hz, 1H), 4.16 – 3.93 (m, 3H), 3.94 – 3.81 (m, 5H), 2.36 (s, 4H), 1.98 (s, 1H), 1.20 (t, *J* = 7.1 Hz, 1H), 1.06 – 0.92 (m, 4H). **^{13}C-NMR (75 MHz, $CDCl_3$)** *δ* (ppm):143.5 (C), 136.0 (C), 129.3 (CH), 128.7 (C), 127.7

(CH), 111.8 (CH), 83.7 (C), 78.2 (C), 50.4 (CH_2), 47.9 (CH_2), 36.0 (CH_2), 21.4 (CH_3), 2.5 (CH_2), 1.8 (CH_2). To a suspension of NaH (39.5 mg, 0.987 mmol) in THF (10 ml), previously stirred for 10 min, a solution of **S_2** (250 mg, 0.858 mmol) in THF (2 ml) was added at 0 °C. After stirring the reaction mixture for 15 min, 1-bromopent-2-yne (519 mg, 4.29 mmol) was added and the reaction mixture was stirred overnight. Then, the reaction was quenched with water and extracted with Et_2O (x3). The combined organic layers were dried (Na_2SO_4) and concentrated under reduced pressure. The residue was purified by flash column chromatography (10% Et_2O/Hexane) to obtain **1a** as a colourless oil (200 mg, 65 % yield). **^{1}H-NMR (300 MHz, $CDCl_3$)** δ (ppm): 7.72 (d, J = 8.2 Hz, 2H), 7.28 (d, J = 8.2 Hz, 2H), 5.68 (tq, J = 6.8, 2.0 Hz, 1H), 4.08 (s, 2H), 4.00 – 3.87 (m, 5H), 2.40 (s, 3H), 2.21 (qt, J = 7.5, 2.2 Hz, 2H), 2.13 (s, 1H), 1.12 (t, J = 7.5 Hz, 3H), 1.07 (s, 3H). **^{13}C-NMR (75 MHz, $CDCl_3$)** δ (ppm): 143.3 (C), 136.2 (C), 129.4 (C), 128.6 (C), 127.7 (C), 111.9 (CH), 88.8 (C), 80.6 (C), 79.5 (C), 74.3 (C), 56.9 (CH_2), 56.1 (CH_2), 47.9 (CH_2), 36.0 (CH_2), 21.4 (CH_3), 13.7 (CH_3), 12.3 (CH_2), 2.5 (CH_2), 1.8 (CH_2). **HRMS** (APCI-TOF): calculated for $C_{21}H_{26}NO_3S$ $[M + H]^+$: m/z 372.1628, found 372.1641.

(*Z*)-(2-((4-(But-2-yn-1-yloxy)but-2-en-1-yl)oxy)ethylidene)cyclopropane (1q)

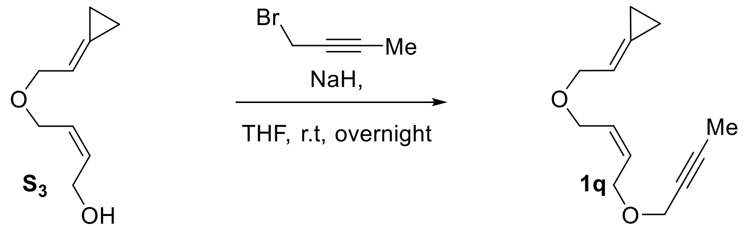

S_3 was obtained following the methodology described for **S_1**, using (*Z*)-4-((*tert*-butyldimethylsilyl)oxy)but-2-en-1-ol, with subsequent silyl ether TBAF-promoted deprotection (conditions for obtaining **S_2**).[3] To a suspension of NaH (77.8 mg, 1.94 mmol) in THF (10 ml), previously stirred for 10 min, a solution of **S_3** (150 mg, 0.97 mmol) in THF (3 ml) was added at 0 °C. After stirring the reaction mixture for 15 min, 1-bromobut-2-yne (388.1 mg, 2.92 mmol) was added and the reaction mixture was stirred overnight. Then, the reaction was quenched with water and extracted with Et_2O (x3). The combined organic layers were dried (Na_2SO_4) and concentrated under reduced pressure. The residue was purified by flash column chromatography (5% EtOAc/Hexane) to obtain **1q** as a colourless oil (168 mg, 83 % yield). **^{1}H-NMR (300 MHz, $CDCl_3$)** δ (ppm): 5.93 (m, 1H), 5.83 – 5.63 (m, 2H), 4.23 – 3.99 (m, 8H), 1.85 (s, 3H), 1.09 (s, 4H). **^{13}C-NMR (75 MHz, $CDCl_3$)** δ (ppm): 130.2 (CH), 128.5 (CH), 126.8 (C), 114.7 (CH), 82.4 (C), 75.0 (C), 70.3 (CH_2), 65.5 (CH_2), 65.0 (CH_2), 57.7 (CH_2), 3.5 (CH_3), 2.2 (CH_2), 1.7 (CH_2). **HRMS** (APCI-TOF): calculated for $C_{13}H_{19}O_2$ $[M + H]^+$: m/z 207.1380, found 207.1387.

N-(4-(Allyloxy)but-2-yn-1-yl)-N-(2-cyclopropylideneethyl)-4-methylbenzenesulfo-namide (1b).[2]

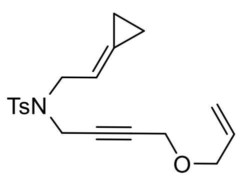

1b

Following the same procedure as for **1a** but using allyl bromide in the last step. Purified through flash column chromatography (25% Et_2O/Hexane) to obtain 500 mg (62% yield). White solid.[2] **^{1}H-NMR (300 MHz, $CDCl_3$)** *δ* (ppm): δ 7.70 (d, *J* = 8.3 Hz, 2H), 7.25 (d, *J* = 7.9 Hz, 2H), 5.86 – 5.70 (m, 1H), 5.66 (tt, *J* = 6.8, 2.0 Hz, 1H), 5.23 – 5.05 (m, 2H), 4.07 (d, *J* = 1.9 Hz, 2H), 3.92 (d, *J* = 7.0 Hz, 2H), 3.84 (s, 2H), 3.79 (dd, *J* = 5.6, 1.4 Hz, 2H), 2.37 (s, 3H), 1.04 (t, *J* = 1.9 Hz, 4H). **^{13}C-NMR (75 MHz, $CDCl_3$)** *δ* (ppm): 143.3 (C), 136.1 (C), 133.8 (C), 129.3 (CH), 128.6 (C), 127.7 (CH), 117.5 (CH_2), 111.9 (CH), 81.2 (C), 79.1 (C), 70.2 (CH_2), 57.0 (CH_2), 47.9 (CH_2), 36.0 (CH_2), 21.4 (CH_3), 2.5 (CH_2), 1.8 (CH_2). **HRMS** (APCI-TOF): calculated for $C_{19}H_{24}NO_3S$ $[M + H]^+$: *m/z* 346.1471, found 346.1470.

N-(2-Cyclopropylideneethyl)-4-methyl-N-(4-((2-methylallyl)oxy)but-2-yn-1yl)benze nesulfonamide (1m)

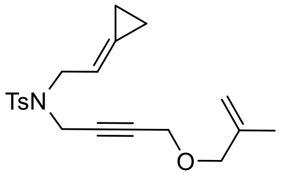

1m

Following the same procedure as for **1a** but using 3-bromo-2-methylpropene in the last step. Purified through flash column chromatography (25% Et_2O/Hexane) to obtain 250 mg (43% yield). Colourless oil.**^{1}H-NMR (300 MHz, $CDCl_3$)** *δ* (ppm): 7.73 (d, *J* = 8.3 Hz, 2H), 7.28 (d, *J* = 8.0 Hz, 2H), 5.70 (ddt, *J* = 6.8, 4.3, 2.1 Hz, 1H), 4.89 (s, 2H), 4.11 (s, 2H), 3.96 (d, *J* = 7.1 Hz, 2H), 3.86 (d, *J* = 2.0 Hz, 2H), 3.74 (s, 2H), 2.40 (s, 3H), 1.69 (s, 3H), 1.16 – 0.97 (m, 4H). **^{13}C-NMR (75 MHz, $CDCl_3$)** *δ* (ppm): 143.3 (C), 141.3 (C), 136.3 (C), 129.4 (CH_2), 128.7 (C), 127.8 (CH), 112.8 (CH), 112.0 (CH), 81.3 (C), 79.2 (C), 73.4 (CH_2), 56.9 (CH_2), 48.0 (CH_2), 36.1 (CH_2), 21.5 (CH_3), 19.4 (CH_3), 2.6 (CH_2), 1.9 (CH_2). **HRMS** (APCI-TOF): calculated for $C_{20}H_{26}NO_3S$ $[M + H]^+$: *m/z* 360.1628, found 360.1630.

General procedure B: assembly through a Mitsunobu reaction

Diethyl 2-allyl-2-(4-((N-(2-cyclopropylideneethyl)-4-methylphenyl)sulfonamido)but-2-yn-1-yl)malonate (1c)

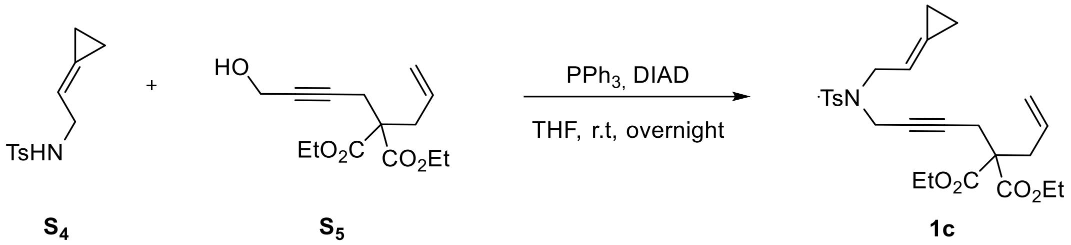

To a solution of alkylidenecyclopropyl *tert*-butyl tosylcarbamate (**S_4**, 200 mg, 0.843 mmol) and PPh_3 (265.8 mg, 1.01 mmol) in THF (2 mL) was added diethyl 2-allyl-2-(4-

hydroxybut-2-yn-1-yl)malonate (**S5**, 249 mg, 0.928 mmol) and the resulting mixture was stirred for 5 min. Then, the reaction was cooled down to 0 °C and diisopropyl azodicarboxylate (0.2 mL, 1.01 mmol) was added dropwise. After stirring at room temperature for 18 h, the reaction was quenched with water. The mixture was extracted with EtOAc and the combined organic phases were dried over anhydrous Na_2SO_4 and concentrated in vacuo. The residue was purified by flash column chromatography (10 % EtOAc / Hexane) to afford 300 mg of **1c** as a colourless oil (73 % yield). **^{1}H-NMR (300 MHz, $CDCl_3$)** δ (ppm): 7.68 (d, *J* = 8.3 Hz, 2H), 7.26 (d, *J* = 8.7 Hz, 2H), 5.65 (tt, *J* = 6.8, 1.9 Hz, 1H), 5.46 (ddt, *J* = 16.4, 10.2, 7.5 Hz, 1H), 5.08 – 4.89 (m, 2H), 4.11 (qd, *J* = 7.1, 1.5 Hz, 4H), 4.00 (d, *J* = 2.1 Hz, 2H), 3.89 (d, *J* = 6.8 Hz, 2H), 2.60 – 2.46 (m, 4H), 2.38 (s, 3H), 1.18 (t, *J* = 7.2 Hz, 6H), 1.04 (s, 4H). **^{13}C-NMR (75 MHz, $CDCl_3$)** δ (ppm): 169.4 (CO), 143.2 (C), 136.5 (C), 131.7 (CH), 129.4 (CH), 127.5 (CH), 119.4 (CH_2), 112.1 (CH), 79.9 (C), 76.2 (C), 61.5 (CH_2), 56.4 (C), 47.5 (CH_2), 36.3 (CH_2), 35.9 (CH_2), 22.6 (CH_2), 21.4 (CH_3), 13.9 (CH), 2.5 (CH_2), 1.8 (CH_2). **HRMS** (APCI-TOF): calculated for $C_{26}H_{34}NO_6S$ [M+H]: *m/z* 488.2101, found 488.2112.

N-(2-Cyclopropylideneethyl)-4-methyl-N-(oct-7-en-2-yn-1-yl)benzenesulfonamide (1d)

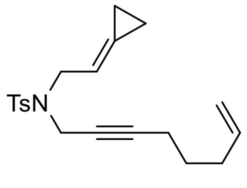

1d

Prepared following the same procedure as used for **1c** using oct-7-en-2-yn-1-ol. Purified through flash column chromatography (5% EtOAc/Hexane) to obtain 174 mg (60% yield). Colourless oil. **^{1}H-NMR (300 MHz, $CDCl_3$)** δ (ppm): 7.71 (d, *J* = 8.3 Hz, 2H), 7.25 (d, *J* = 8.4 Hz, 2H), 5.69 (ddt, *J* = 8.8, 4.6, 2.1 Hz, 2H), 4.99 – 4.93 (m, 1H), 4.91 (d, J = 1.7 Hz, 1H), 4.01 (s, 2H), 3.94 (d, *J* = 5.3 Hz, 2H), 2.38 (s, 3H), 1.98 – 1.82 (m, 4H), 1.31 (pd, *J* = 7.2, 2.1 Hz, 2H), 1.05 (s, 4H). **^{13}C-NMR (75 MHz, $CDCl_3$)** δ (ppm): 143.1 (C), 137.5 (CH), 136.4 (C), 129.2 (CH), 128.2 (C), 127.7 (CH), 115.1 (CH_2), 112.2 (CH), 85.4 (C), 72.8 (C), 47.6 (CH_2), 36.2 (CH_2), 32.6 (CH_2), 27.5 (CH_2), 21.4 (CH_3), 17.8 (CH_2), 2.5 (CH_2), 1.8 (CH_2). **HRMS** (APCI-TOF): calculated for $C_{20}H_{26}NO_2S$ $[M+H]^+$ *m/z* 344.1679, found 344.1682.

N-Allyl-N-(4-((N-(2-cyclopropylideneethyl)-4-methylphenyl)sulfonamido)but-2-yn-1-yl)-4-methylbenzenesulfonamide (1j)

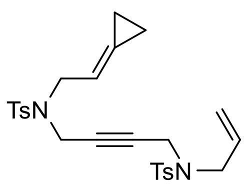

1j

Prepared following the same procedure as used for **1c** using N-allyl-N-(4-hydroxybut-2-yn-1-yl)-4-methylbenzenesulfonamide. Purified through flash column chromatography (10% to 20% EtOAc/Hexane) to obtain 267 mg (53% yield). Colourless solid. **^{1}H-NMR (300 MHz, $CDCl_3$)** δ (ppm): 7.61 (d, *J* = 8.5 Hz, 4H), 7.25 (d, *J* = 8.4 Hz, 4H), 5.64

– 5.49 (m, 2H), 5.18 – 4.92 (m, 2H), 3.82 (s, 4H), 3.77 (d, *J* = 7.0 Hz, 2H), 3.59 (d, *J* = 6.4 Hz, 2H), 2.37 (s, 6H), 1.10 – 0.99 (m, 2H), 0.98 – 0.87 (m, 2H). **^{13}C-NMR (75 MHz, $CDCl_3$)** *δ* (ppm): 143.7 (C), 143.5 (C), 136.3 (C), 136.0 (C), 131.8 (CH), 129.5 (CH), 129.4 (CH), 128.4 (C), 127.5 (CH), 127.5 (CH), 127.0 (C), 119.6 (CH_2), 111.8 (CH), 78.7 (C), 77.9 (C), 48.9 (CH_2), 47.8 (CH_2), 35. (CH_2)5, 35.7 (CH_2), 21.4 (CH_3), 2.5 (CH_2), 1.8 (CH_2). **HRMS** (APCI-TOF): calculated for $C_{26}H_{31}N_2O_4S_2$ $[M+H]^+$ *m/z* 499.1720, found 499.1727.

N-(4-(Allyloxy)but-2-yn-1-yl)-N-(2-cyclopropylidenepropyl)-4-nitrobenzenesulfonamide (1o)

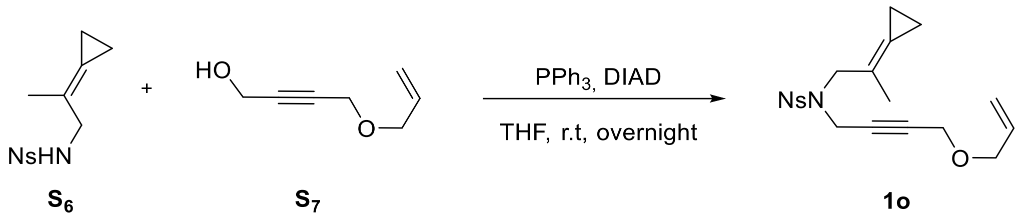

S6 **S7** **1o**

Carried out with alkylidenecyclopropyl nosylcarbamate (**S6**, 141 mg, 0.50 mmol) and 4-(allyloxy)but-2-yn-1-ol (**S7**) to afford of **1o** (colourless oil, 150 mg, 77 % yield, purified by flash column chromatography with 10 % EtOAc/Hexanes). **^{1}H-NMR (300 MHz, $CDCl_3$)** *δ* (ppm): 8.35 (d, *J* = 8.9 Hz, 2H), 8.06 (d, *J* = 8.8 Hz, 2H), 5.79 (ddt, *J* = 16.6, 11.1, 5.7 Hz, 1H), 5.22 (dd, *J* = 6.9, 1.6 Hz, 1H), 5.18 (s, 1H), 4.08 (s, 2H), 3.93 (s, 2H), 3.87 – 3.71 (m, 4H), 1.85 (d, *J* = 1.8 Hz, 3H), 1.07 (s, 4H). **^{13}C-NMR (75 MHz, $CDCl_3$)** *δ* (ppm): 150.0 (C), 144.9 (C), 133.5 (CH), 128.9 (CH), 124.0 (CH), 123.7 (C), 117.7 (C), 117.5 (CH_2), 81.8 (C), 78.3 (C), 70.5 (CH_2), 56.8 (CH_2), 51.9 (CH_2), 35.7 (CH_2), 17.9 (CH_3), 2.7 (CH_2), 2.3 (CH_2). **HRMS** (APCI-TOF): calculated for $C_{19}H_{23}N_2O_5S$ $[M+H]^+$ *m/z* 391.1322, found 391.1349.

General procedure C: assembly through a Tsuji-Trost reaction

(2-((4-(Allyloxy)but-2-yn-1-yl)oxy)ethylidene)cyclopropane (1i)[4]

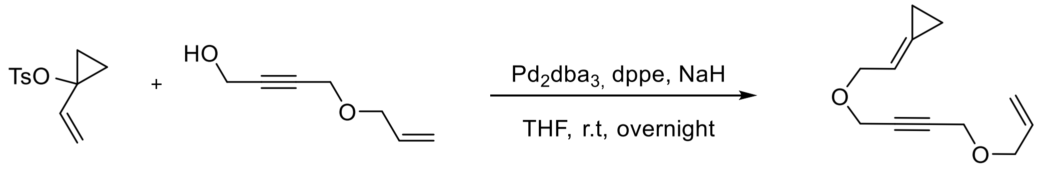

S8 **1i**

4-(Allyloxy)but-2-yn-1-ol (**S8**, 300 mg, 2.38 mmol) was added slowly to a suspension of NaH (96 mg, 4.00 mmol) in THF (15 mL), cooled at 0°C. After stirring for 15 min, a solution of 1-vinylcyclopropyl 4-methylbenzenesulfonate (566 mg, 2.38 mmol), $Pd_2(dba)_3$ (22 mg, 0.024 mmol) and dppe (19 mg, 0.048 mmol) in THF (5 mL), (previously stirred for 20 min), was added "via cannula". The reaction mixture was stirred overnight at rt, poured into water and extracted with Et_2O (3 x 20 mL). The organic phases were dried, filtered and concentrated to give a crude oily residue that was purified by flash

chromatography (10 % Et_2O/hexanes) to yield 232 mg of **1i** as pale yellow oil (51 % yield).[4] **^{1}H-NMR (300 MHz, $CDCl_3$)** δ (ppm): δ 6.01-5.68 (m, 2H), 5.21 (d, *J* = 17.1 Hz, 1H), 5.12 (d, *J* = 10.4 Hz, 1H), 4.22-4.04 (m, 6H), 4.03- 3.89 (m, 2H), 1.02 (s, 4H); **^{13}C-NMR (75 MHz, $CDCl_3$)** δ (ppm): 133.6 (CH), 127.3 (C), 117.2 (CH_2), 113.7 (CH), 82.2 (C), 81.6 (C), 70.0 (CH_2), 69.3 (CH_2), 56.9 (CH_2), 56.6 (CH_2), 1.9 (CH_2), 1.4 (CH_2). **HRMS** (APCI-TOF): calculated for $C_{12}H_{17}O_2$ [M + H]$^+$: *m/z* 193.1223, found 193.1224.

Dimethyl 2-(4-(allyloxy)but-2-yn-1-yl)-2-(2-cyclopropylideneethyl)malonate (1e)[4]

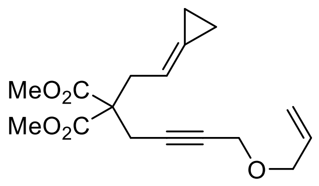

1e

Prepared following the same procedure used for **1i** but using dimethyl 2-(4-(allyloxy)but-2-yn-1-yl)malonate. Purified through flash column chromatography (15% Et_2O/Hexane) to obtain 230 mg (77% yield). Colourless oil. **^{1}H-NMR (300 MHz, $CDCl_3$)** δ (ppm): 5.86 (ddt, *J* = 16.5, 11.3, 5.9 Hz, 1H), 5.54 (t, *J* = 7.7 Hz, 1H), 5.27 (d, *J* = 17.1 Hz, 1H), 5.17 (d, *J* = 10.3 Hz, 1H), 4.09 – 4.05 (m, 2H), 3.99 (d, *J* = 5.9 Hz, 2H), 3.69 (s, 6H), 2.90 (d, *J* = 7.5 Hz, 2H), 2.80 (d, *J* = 2.5 Hz, 2H), 1.11 – 0.92 (m, 4H). **^{13}C NMR** (75 MHz, $CDCl_3$) δ 170.4 (CO), 134.0 (CH), 127.3 (C), 117.6 (CH_2), 111.2 (CH), 81.4 (C), 78.8 (C), 70.1 (CH_2), 57.3 (C), 57.2 (CH_2), 52.6 (CH_3), 34.7 (CH_2), 23.0 (CH_2), 2.9 (CH_2), 1.8 (CH_2). **HRMS** (APCI-TOF): calculated for $C_{17}H_{23}O_5$ [M + H]$^+$: *m/z* 307.1540 found 307.1545.

Tetraethyl 1-cyclopropylideneundec-10-en-5-yne-3,3,8,8-tetracarboxylate (1f)[4]

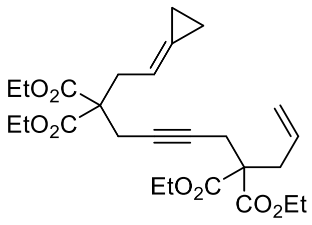

1f

Prepared following the same procedure used for **1i** but using tetraethyl non-8-en-3-yne-1,1,6,6-tetracarboxylate. Purified through flash column chromatography (15% Et_2O/Hexane) to obtain 210 mg (71% yield). Colourless oil.[2] **^{1}H-NMR (300 MHz, $CDCl_3$)** δ (ppm): 5.62 (dd, *J* = 16.2, 8.3 Hz, 2H), 5.18 (d, *J* = 17.1 Hz, 1H), 5.11 (d, *J* = 10.1 Hz, 1H), 4.19 (q, *J* = 7.3 Hz, 8H), 2.89 (d, *J* = 7.5 Hz, 2H), 2.75 (s, 6H), 1.24 (t, *J* = 7.1 Hz, 12H), 1.04 (s, 4H). **^{13}C-NMR (75 MHz, $CDCl_3$)** δ (ppm): 170.1 (CO), 169.8 (CO), 132.0 (CH), 126.9 (C), 119.6 (CH_2), 111.7 (CH), 78.1 (C), 77.5 (C), 61.5 (CH_2), 61.4 (CH_2), 57.2 (C), 56.8 (C), 36.3 (CH_2), 34.5 (CH_2), 22.8 (CH_2), 14.1 (CH_3), 2.9 (CH_2), 2.0 (CH_2). **HRMS** (APCI-TOF): calculated for $C_{26}H_{37}O_8$ [M + H]$^+$: *m/z* 477.2488 , found 477.2486.

N-Allyl-N-(4-(2-cyclopropylideneethoxy) but-2-yn-1-yl)-4-methylbenzenesulfonamide (1g)

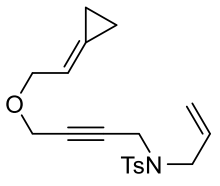

1g

Prepared following the same procedure as used for **1i** using N-allyl-N-(4-hydroxybut-2-yn-1-yl)-4-methylbenzenesulfonamide. Purified through flash column chromatography (10% to 20% EtOAc/Hexane) to obtain 276 mg (74% yield). Colourless oil. **^{1}H-NMR (300 MHz, $CDCl_3$)** δ (ppm): 7.71 (d, *J* = 6.2 Hz, 2H), 7.27 (d, *J* = 6.3 Hz, 2H), 5.89 – 5.80 (m, 1H), 5.70 (dtd, *J* = 16.6, 7.3, 6.5, 3.0 Hz, 1H), 5.31 – 5.12 (m, 2H), 4.11 (s, 2H), 3.98 (d, *J* = 6.0 Hz, 2H), 3.86 (s, 2H), 3.80 (d, *J* = 6.5 Hz, 2H), 2.38 (s, 3H), 1.11 – 0.94 (m, 4H). **^{13}C-NMR (75 MHz, $CDCl_3$)** δ (ppm): 143.4 (C), 136.1 (C), 132.0 (CH), 129.4 (CH), 127.7 (CH), 119.7 (CH_2), 113.9 (CH), 81.8 (C), 78.7 (C), 69.5 (CH_2), 56.7 (CH_2), 49.1 (CH_2), 36.1 (CH_2), 21.4 (CH_3), 2.3 (CH_2), 1.7 (CH_2). **HRMS** (APCI-TOF): calculated for $C_{19}H_{24}NO_3S$ $[M+H]^+$ *m/z* 346.1471 found 346.1474.

Diethyl 2-allyl-2-(4-(2-cyclopropylideneethoxy)but-2-yn-1-yl)malonate (1h)

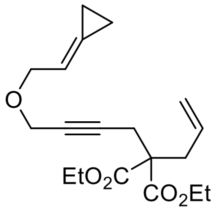

1h

Prepared following the same procedure as used for **1i** using diethyl 2-allyl-2-(4-hydroxybut-2-yn-1-yl)malonate. Purified through flash column chromatography (15% Et_2O/Hexane) to obtain 220 mg (82% yield). Colourless oil. **^{1}H-NMR (300 MHz, $CDCl_3$)** δ (ppm): 5.97 – 5.86 (m, 1H), 5.65 (tt, *J* = 17.1, 7.9 Hz, 1H), 5.25 – 5.07 (m, 2H), 4.30 – 4.13 (m, 8H), 2.84-2.70 (m, 4H), 1.31 – 1.19 (m, 6H), 1.11 (s, 4H). **^{13}C-NMR (75 MHz, $CDCl_3$)** δ (ppm): 169.8 (CO), 131.9 (CH), 127.8 (C), 119.7 (CH_2), 114.2 (CH), 81.0 (C), 79.4 (C), 69.5 (CH_2), 61.6 (CH_2), 57.2 (CH_2), 56.8 (C), 36.6 (CH_2), 23.0 (CH_2), 14.1 (CH_3), 2.4 (CH_2), 1.8 (CH_2). **HRMS** (APCI-TOF): calculated for $C_{19}H_{27}O_5$ $[M+H]^+$ *m/z* 335.1853 found 335.1884.

General procedure D: assembly through tosylate substitution

Diethyl 2-(2-cyclopropylideneethyl)-2-(4-((2-methylallyl)oxy)but-2-yn-1-yl)malonate (1n)

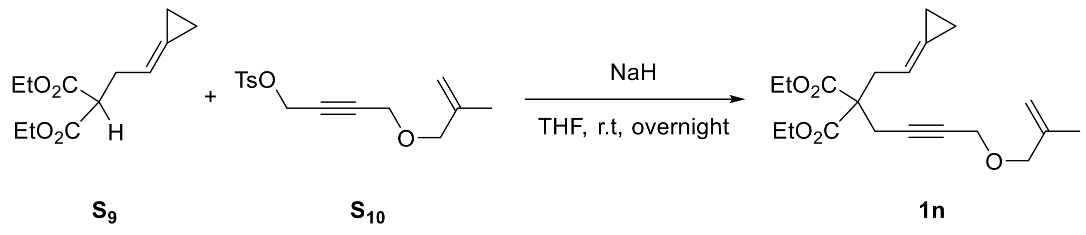

To a suspension of NaH (21.2 mg, 0.53 mmol) in THF (2 ml), previously stirred for 10 min, a solution of **S_9** (100 mg, 0.44 mmol) in THF (2 ml) was added at 0 °C. After stirring the reaction mixture for 15 min, a solution of **S_{10}** (195 mg, 0.66 mmol) in THF (2 mL) was added and the reaction mixture was stirred overnight at rt. Then, the reaction was quenched with water and extracted with Et_2O (x3). The combined organic layers were dried (Na_2SO_4) and concentrated under reduced pressure. The residue was purified by flash column cromatography (10% AcOEt/Hexane) to obtain **3n** as a colourless oil (143 mg, 93 % yield). **^{1}H-NMR (300 MHz, $CDCl_3$)** δ (ppm): 5.57 (tt, J = 7.6, 5.6, 2.2 Hz, 1H), 4.95 (s, 1H), 4.88 (s, 1H), 4.16 (q, J = 7.1 Hz, 4H), 4.06 (d, J = 2.3 Hz, 2H), 3.90 (s, 2H), 2.91 (d, J = 7.5 Hz, 2H), 2.81 (s, 2H), 1.71 (s, 3H), 1.31 – 1.16 (m, 6H), 1.03 (s, 4H). **^{13}C-NMR (75 MHz, $CDCl_3$)** δ (ppm): 169.9 (CO), 141.5 (C), 127.0 (C), 112.7 (CH_2), 111.4 (CH), 81.4 (C), 78.8 (C), 73.1 (CH_2), 61.4 (CH_2), 57.1 (CH_2), 57.0 (C) 34.6 (CH_2), 22.9 (CH_2), 19.4 (CH_3), 13.9 (CH_3), 2.8 (CH_2), 1.8 (CH_2). **HRMS** (APCI-TOF): calculated for $C_{20}H_{29}O_5$ $[M+H]^+$ m/z 349.2010 found 349.2014.

3. Preliminary investigations with ACP precursors 1a and 1q

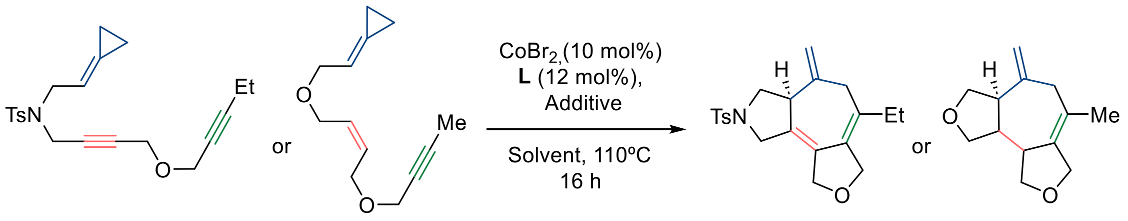

Entry	ACP	Additive	L	Solvent	Yield (%)
1	**1a**	In (50 mol%) / $InBr_3$ (20 mol%)	Binap	1,2-DCE	0 [b]
2	**1a**	In (50 mol%) / $InBr_3$ (20 mol%)	dppp	1,2-DCE	0 [b]
3	**1a**	Zn (50 mol%) / $ZnBr_2$ (20 mol%)	Binap	1,2-DCE	0 [b]
4	**1a**	In (50 mol%) / NaBArF (20 mol%)	Binap	1,2-DCE	0 [b]
5	**1a**	In (50 mol%) / $InBr_3$ (20 mol%)	Binap	MeCN	0 [b]
6	**1a**	In (50 mol%) / $InBr_3$ (20 mol%)	dppp	MeCN	0 [b]
7	**1q**	Zn (50mol%) / $ZnBr_2$ (20 mol%)	dppp	1,2-DCE	0 [c]

Conditions: **1a**, $CoBr_2$ (10 mol%), **L** (12 mol%) and additive were heated in the appropriate solvent for 16 h at 110 ºC, unless otherwise noted. Null conversions were observed in all cases. [b] Diyne **1b** was mainly recovered. [c] Full conversion of **1q** was observed to give a complex mixture of products.

4. General procedure for the intramolecular (3+2+2) cycloaddition (exemplified for **1b**).

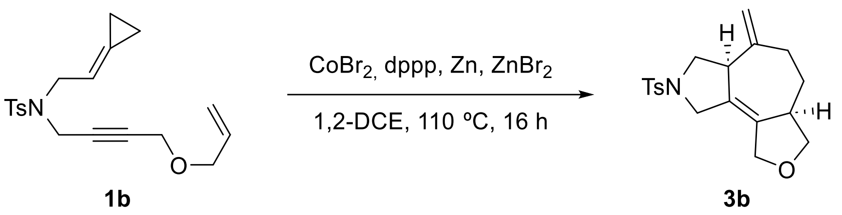

$CoBr_2$ (2.2 mg, 0.01 mmol), dppp (4.9 mg, 0.012 mmol), Zn (3.3 mg, 0.05 mmol) and $ZnBr_2$ (4.5 mg, 0.02 mmol) were successively added to a dried Schlenk tube under Argon. Then, **1b** (34.5 mg, 0.100 mmol) in 1,2-dichloroethane (1 mL) was added under Ar atmosphere and the resulting mixture was stirred for 16 h at 110 °C before it was filtered through a short pad of Florisil® and concentrated under vacuum. The residue was purified by flash column chromatography (25 % Et_2O / hexanes) to give 22 mg of **3b** (63% yield) as a white solid.[2] **^{1}H-NMR (300 MHz, $CDCl_3$)** δ (ppm): 7.72 (d, J = 8.2 Hz, 2H), 7.35 (d, J = 8.3 Hz, 2H), 4.77 (s, 1H), 4.66 (s, 1H), 4.26 – 4.02 (m, 3H), 3.73 (d, J = 13.4 Hz, 1H), 3.60-3.40 (m, 2H), 3.39- 3.31 (m, 2H), 3.15 (t, J = 8.5 Hz, 1H), 2.89 – 2.72 (m, 1H), 2.58 – 2.44 (m, 1H), 2.42 (s, 3H), 2.26 – 2.01 (m, 2H), 1.92 – 1.78 (m, 1H). **^{13}C-NMR (75 MHz, $CDCl_3$)** δ (ppm): 145.6 (C), 143.9 (C), 135.5 (C), 131.8 (C), 129.7 (CH), 128.0 (CH), 126.8 (C), 109.2 (CH_2), 75.0 (CH_2), 70.8 (CH_2), 51.6 (CH_2), 51.0 (CH_2), 48.0 (CH), 43.9 (CH), 37.8 (CH_2), 30.9 (CH_2), 21.5 (CH_3). **HRMS** (CI): calculated for $C_{19}H_{23}NO_3S$ [M]: *m/z* 345.1399, found 345.1412.

<u>**1 mmol scale experiment**</u>: $CoBr_2$ (22 mg, 0.1 mmol), dppp (49 mg, 0.12 mmol), Zn (33 mg, 0.5 mmol) and $ZnBr_2$ (45 mg, 0.2 mmol) were successively added to a dried Schlenk tube under Argon. Then, **1b** (345 mg, 1 mmol) in 1,2-dichloroethane (10 mL) was added under Ar atmosphere and the resulting mixture was stirred for 16 h at 110 °C before it was filtered through a short pad of Florisil® and concentrated under vacuum. The residue was purified by flash column chromatography (25 % Et_2O / hexanes) to give 209 mg of **3b** (61% yield) as a white solid.

Diethyl (3aS,6aS)-4-methylene-2-tosyl-1,2,3,3a,4,5,6,6a,7,9-decahydro-8H-azuleno[4,5-c]pyrrole-8,8-dicarboxylate (3c)

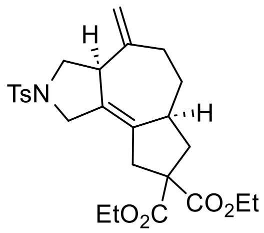

3c

Purified through flash column chromatography (25% EtOAc/Hexane) to obtain 37 mg (76% yield) of **3c** as a colourless oil. **^{1}H-NMR (500 MHz, $CDCl_3$)** δ (ppm): 7.72 (d, J = 8.3 Hz, 2H), 7.35 (d, J = 7.9 Hz, 2H), 4.72 (s, 1H), 4.62 (s, 1H), 4.24 – 4.11 (m, 4H), 3.81 (d, J = 13.5 Hz, 1H), 3.59 – 3.50 (m, 1H), 3.48 (dd, J = 9.3, 7.5 Hz, 1H), 3.30 (s, 1H), 3.20 (dd, J = 9.3, 8.0 Hz, 1H), 2.85 – 2.72 (m, 2H), 2.71 – 2.65 (m, 1H), 2.60 – 2.48 (m, 2H), 2.44 (s, 3H), 2.08 (td, J = 12.6, 3.0 Hz, 1H), 1.89 (ddt, J = 12.7, 6.2, 3.2 Hz, 1H), 1.76 (dd, J = 12.9, 10.6 Hz, 1H), 1.40 (s, 1H), 1.24 (td, J = 7.1, 4.7 Hz, 6H), 1.13 (dtd, J = 14.2, 11.9, 2.8 Hz, 1H). **^{13}C-NMR (126 MHz, $CDCl_3$)** δ (ppm): 171.5 (CO), 171.4 (CO), 146.3 (C), 143.9 (C), 135.6 (C), 131.9 (C) 129.8 (CH), 128.3 (CH), 108.3 (CH_2), 61.7 (CH_2), 59.0 (C), 52.1 (CH_2), 52.0 (CH_2), 47.7 (CH), 43.3 (CH), 41.8 (CH_2), 39.8 (CH_2), 39.3 (CH_2), 34.3 (CH_2), 21.7 (CH_3), 14.1 (CH_3). **HRMS** (APCI-TOF): calculated for $C_{26}H_{34}NO_6S$ [M+H]: m/z 488.2101, found 488.2112.

(3aS,6aR)-4-Methylene-2-tosyl-2,3,3a,4,5,6,6a,7,8,9-decahydro-1H-azuleno[4,5-c]pyrrole (3d)

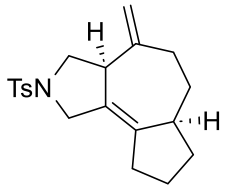

3d

Purified through flash column chromatography (5% Et_2O/Hexane) to obtain 22 mg (64% yield) of **3d** as a pale yellow oil. **^{1}H-NMR (300 MHz, $CDCl_3$)** δ (ppm): 7.73 (d, J = 8.3 Hz, 2H), 7.34 (d, J = 7.7 Hz, 2H), 4.71 (s, 1H), 4.63 (s, 1H), 3.81 – 3.70 (m, 1H), 3.58 (dt, J = 13.4, 2.6 Hz, 1H), 3.47 (dd, J = 9.1, 7.5 Hz, 1H), 3.34 (q, J = 5.4 Hz, 1H), 3.18 (dd, J = 9.1, 7.7 Hz, 1H), 2.57 – 2.48 (m, 2H), 2.43 (s, 3H), 2.17 – 2.01 (m, 3H), 1.94 – 1.80 (m, 3H), 1.71 – 1.64 (m, 1H), 1.47 (dddd, J = 16.7, 8.4, 3.9, 1.8 Hz, 1H), 1.25 (dtt, J = 6.2, 3.9, 2.4 Hz, 1H), 1.15 – 1.08 (m, 1H). **^{13}C-NMR (75 MHz, $CDCl_3$)** δ (ppm): 146.9 (C), 143.6 (C), 139.8 (C), 129.6 (CH), 128.1 (CH), 127.4 (C), 107.9 (CH_2), 52.1 (CH_2), 51.8 (CH_2), 47.7 (CH), 44.1 (CH), 38.8 (CH_2), 35.1 (CH_2), 34.5 (CH_2), 32.7 (CH_2), 25.0 (CH_2), 21.5 (CH_2). **HRMS** (APCI-TOF): calculated for $C_{20}H_{26}NO_2S$ [M+H]$^+$ m/z 344.1679 found 344.1682.

Dimethyl (3aR,6aS)-6-methylene-1,3a,4,5,6,6a,7,9-octahydroazuleno[4,5-c]furan-8,8(3H)-dicarboxylate (3e)

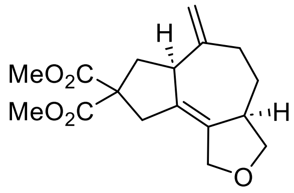

3e

Purified through flash column chromatography (25% Et_2O/Hexane) to obtain 22 mg (73% yield) of **3e** as a colourless oil. **^{1}H-NMR (300 MHz, $CDCl_3$)** δ (ppm): 4.78 (s, 1H), 4.74 (s, 1H), 4.34 (d, J = 13.3 Hz, 1H), 4.20 – 4.07 (m, 2H), 3.74 (d, J = 2.6 Hz, 6H), 3.37 (t, J = 8.6 Hz, 1H), 3.30 – 3.21 (m, 1H), 2.90-2.70 (m, 2H), 2.63 (ddd, J = 13.2, 6.2, 2.5 Hz, 1H), 2.52 (ddd, J = 12.8, 7.0, 1.6 Hz, 1H), 2.32 (t, J = 12.4 Hz, 1H), 2.19 – 2.06 (m, 1H), 1.87 (ddd, J = 13.3, 6.4, 3.2 Hz, 1H), 1.32 – 1.09 (m, 2H) . **^{13}C-NMR (75 MHz, $CDCl_3$)** δ (ppm): 171.8 (C), 147.9 (C), 135.2 (C), 129.7 (C), 108.2 (CH_2), 75.4 (CH_2), 71.6 (CH_2), 58.6 (C), 52.8 (CH_3), 48.0 (CH), 44.1 (CH), 39.1 (CH_2), 38.6 (CH_2), 38.0 (CH_2), 30.8 (CH_2). **HRMS** (APCI-TOF): calculated for $C_{17}H_{23}O_5$ [M+H]: *m/z* 307.1467 found 307.1468.

Tetraethyl (3aS,6aS)-4-methylene-1,3a,4,5,6,6a,7,9-octahydro-2H-cyclopenta[e]azulene-2,2,8,8(3H)-tetracarboxylate (3f)[4]

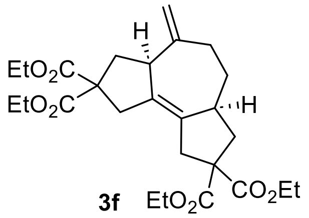

3f

Purified through flash column chromatography (25% EtOAc/Hexane) to obtain 37 mg (77% yield). Colourless oil. **^{1}H NMR (300 MHz, $CDCl_3$)** δ (ppm): 4.72 (s, 1H), 4.70 (s, 1H), 4.23-4.14 (m, 8H), 3.18 (m, 1H), 2.94-2.90 (m, 2H), 2.81-2.77 (m, 2H), 2.67 (m, 1H), 2.62-2.53 (m, 2H), 2.52-2.38 (m, 1H), 2.33-2.28 (m, 1H), 2.08 (dt, J = 12.6, 2.6 Hz, 1H), 1.95- 1.86 (m, 1H), 1.80 (dd, J = 12.6, 10.7 Hz, 1H), 1.38-1.14 (m, 12H), 1.13 (d, J = 9.8 Hz, 1H). **^{13}C-NMR (75 MHz, $CDCl_3$)** δ (ppm): 171.6 (CO), 148.6 (C), 135.0 (C), 132.7 (C), 107.3 (CH_2), 61.5 (CH_2), 61.4 (CH_2), 58.8 (C), 58.5 (C), 47.5 (CH), 43.1 (CH), 42.0 (CH_2), 40.5 (CH_2), 39.8 (CH_2), 39.6 (CH_2), 37.9 (CH_2), 34.3 (CH_2), 14.0 (CH_3). **HRMS** (APCI-TOF): calculated for $C_{26}H_{37}O_8$ [M+H]: *m/z* 477.2488, found 477.2489.

(3aS,6aR)-4-Methylene-8-tosyl-1,3,3a,4,5,6,6a,7,8,9decahydrofuro[3',4':3,4]cyclohepta[1,2-c]pyrrole (3g)

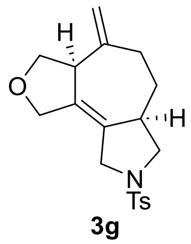

3g

Purified through flash column chromatography (10% EtOAc/Hexane) to obtain 30 mg (88% yield) of **3g**. Colourless oil. **^{1}H-NMR (300 MHz, $CDCl_3$)** δ (ppm): 7.69 (d, J = 8.2 Hz, 2H), 7.33 (d, J = 8.0 Hz, 2H), 4.75 (s, 1H), 4.63 (s, 1H), 4.27 – 4.11 (m, 2H), 4.06 (dd, J = 8.8, 7.4 Hz, 1H), 3.84 (t, J = 8.8 Hz, 1H), 3.75 (d, J = 13.4 Hz, 1H), 3.61 (dd, J = 9.3, 7.8 Hz, 1H),

3.46 (ddq, *J* = 13.8, 4.3, 2.2 Hz, 1H), 3.33 (s, 1H), 2.90 (s, 1H), 2.66 (dd, *J* = 9.3, 8.4 Hz, 1H), 2.54 (ddd, *J* = 13.3, 6.4, 3.1 Hz, 1H), 2.44 (s, 3H), 2.15 (td, *J* = 13.4, 12.4, 3.2 Hz, 1H), 1.88 (ddt, *J* = 13.3, 6.7, 3.5 Hz, 1H), 1.22 (tdd, *J* = 14.6, 9.8, 3.1 Hz, 1H). **^{13}C-NMR (75 MHz, $CDCl_3$)** δ (ppm): 145.5 (C), 143.8 (C), 133.8 (C), 131.9 (C), 129.6 (CH), 128.5 (C), 128.0 (CH), 108.8 (CH_2), 72.0 (CH_2), 70.6 (CH_2), 54.5 (CH_2), 51.4 (CH_2), 49.3 (CH), 42.8 (CH), 38.0 (CH_2), 32.1 (CH_2), 21.5 (CH_3). **HRMS** (APCI-TOF): calculated for $C_{19}H_{24}NO_3S$ $[M+H]^+$ *m/z* 346.1471 found 346.1470.

Diethyl (3aS,6aS)-4-methylene-1,3a,4,5,6,6a,7,9-octahydroazuleno[4,5-c]furan-8,8(3H)-dicarboxylate (3h)

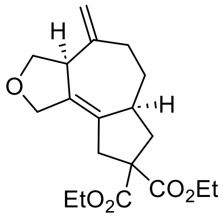

3h

Purified through flash column chromatography (25% Et_2O/Hexane) to obtain 21 mg (64% yield) of **3h**. Colorless oil. **^{1}H-NMR (300 MHz, $CDCl_3$)** δ (ppm): 4.74 (s, 1H), 4.64 (s, 1H), 4.32 (d, *J* = 13.1 Hz, 1H), 4.19 (qt, *J* = 7.1, 3.7 Hz, 5H), 4.08 (dd, *J* = 8.8, 7.4 Hz, 1H), 3.95 (t, *J* = 8.5 Hz, 1H), 3.31 (s, 1H), 2.77 (t, *J* = 2.8 Hz, 3H), 2.69 – 2.51 (m, 2H), 2.16 (td, *J* = 12.6, 2.8 Hz, 1H), 2.01 – 1.90 (m, 1H), 1.90 – 1.72 (m, 1H), 1.24 (td, *J* = 7.1, 2.1 Hz, 7H). **^{13}C-NMR (75 MHz, $CDCl_3$)** δ (ppm): 171.5 (CO), 171.4 (CO), 146.7 (C), 133.5 (C), 132.2 (C), 107.4 (CH_2), 72.1 (CH_2), 71.2 (CH_2), 61.5 (CH_2), 48.9 (CH), 43.3 (CH), 41.6 (CH_2), 39.7 (CH_2), 39.4 (CH_2), 34.4 (CH_2), 14.0 (CH_3). **HRMS** (APCI-TOF): calculated for $C_{19}H_{27}O_5$ $[M+H]^+$ *m/z* 335.1853 found 335.1872.

(3aR,6aS)-4-Methylene-1,3a,4,5,6,6a,7,9-octahydro-3H-2,8-dioxacyclopenta[e]azulene (3i)[4]

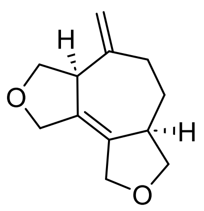

3i

Purified through flash column chromatography (15% Et_2O/Hexane) to obtain 12 mg (58 % yield) of **3i**. Colourless oil. **^{1}H-NMR (300 MHz, $CDCl_3$)** δ (ppm): 4.79 (s, 1H), 4.67 (s, 1H), 4.25-4.06 (m, 6H), 3.93 (t, *J* = 8.6 Hz, 1H), 3.39 (t, *J* = 8.5 Hz, 2H), 2.89-2-86 (m, 1H), 2.62 (ddd, *J* = 13.1, 6.04, 2.7 Hz, 1H), 2.26-2.14 (m, 1H), 1.96-1.86 (m, 1H), 1.45-1.30 (m, 1H); **^{13}C-NMR (75 MHz, $CDCl_3$)** δ (ppm): δ 146.2 (C), 132.3 (C), 130.6 (C), 108.3 (CH_2), 75.0 (CH_2), 71.8 (CH_2), 70.6 (CH_2), 70.4 (CH_2), 49.4 (CH), 44.2 (CH), 38.5 (CH_2), 31.2 (CH_2). **HRMS** (APCI-TOF): calculated for $C_{12}H_{17}O_2$ $[M + H]^+$: *m/z* 193.1223 , found 193.1227.

(3aS,6aR)-4-Methylene-2,8-ditosyl-2,3,3a,4,5,6,6a,7,8,9-decahydro-1H-cyclohepta [1,2-c:3,4-c']dipyrrole (3j)

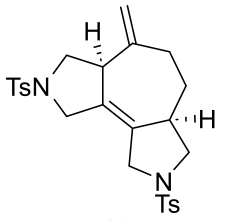
3j

Purified through flash column chromatography (20% EtOAc/Hexane) to obtain 23 mg (46% yield) of **3j**. Colourless solid. **^{1}H-NMR (300 MHz, $CDCl_3$)** δ (ppm): 7.69 (dd, J = 10.7, 8.3 Hz, 4H), 7.34 (t, J = 7.9 Hz, 4H), 4.74 (s, 1H), 4.66 (s, 1H), 4.10 (t, J = 6.8 Hz, 1H), 3.75 – 3.60 (m, 2H), 3.49 (ddd, J = 9.2, 7.6, 4.6 Hz, 4H), 3.37 – 3.28 (m, 1H), 3.10 (dd, J = 9.3, 8.3 Hz, 1H), 2.83 (s, 1H), 2.68 (dd, J = 9.3, 7.6 Hz, 1H), 2.44 (m, 7H), 2.07 (ddd, J = 13.6, 10.3, 3.7 Hz, 1H), 1.81 (ddd, J = 10.2, 5.3, 3.2 Hz, 1H), 1.26 – 1.10 (m, 1H). **^{13}C-NMR (75 MHz, $CDCl_3$)** δ (ppm): 145.0 (C), 144.0 (C), 143.9 (C), 131.9 (C), 131.8 (C), 131.6 (C), 130.0 (C), 129.8 (CH), 129.7 (CH), 128.04 (CH), 128.02 (CH), 109.8 (CH_2), 54.4 (CH_2), 51.8 (CH_2), 51.5 (CH_2), 51.2 (CH_2), 48.1 (CH), 42.2 (CH), 37.0 (CH_2), 32.0 (CH_2), 21.5 (CH_3). **HRMS** (APCI-TOF): calculated for $C_{26}H_{31}N_2O_4S_2$ $[M+H]^+$ m/z 499.1720 found 499.1727.

(3aR,6aS)-3a-Methyl-6-methylene-8-tosyl-1,3,3a,4,5,6,6a,7,8,9-decahydrofuro [3',4':3,4]cyclohepta[1,2-c]pyrrole (3m)

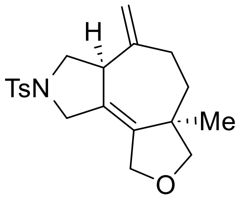
3m

Purified through flash column chromatography (25% Et_2O/Hexane) to obtain 20 mg (56% yield) of **3m** as a colorless oil.**^{1}H-NMR (300 MHz, $CDCl_3$)** δ δ 7.72 (d, J = 8.2 Hz, 2H), 7.35 (d, J = 8.4 Hz, 2H), 4.79 (s, 1H), 4.67 (s, 1H), 4.11 – 3.92 (m, 5H), 3.90 – 3.78 (m, 1H), 3.56 – 3.36 (m, 2H), 3.33-3.25 (m, 2H), 2.97 (d, d, J = 8.9 Hz 1H), 2.79 (s, 1H), 2.44 (s, 3H), 1.87 (m, 1H), 1.27 (s, 3H). **^{13}C-NMR (75 MHz, $CDCl_3$)** δ (ppm): 149.4 (C), 142.7 (C), 133.0 (C), 131.3 (C), 128.7 (CH), 126.9 (CH), 109.1 (CH_2), 74.0 (CH_2), 69.9 (CH_2), 58.1 (CH_2), 49.8 (CH_2), 40.6 (CH), 32.8 (CH_2), 30.1 (CH_2), 23.5 (CH_3), 20.5 (CH_3). **HRMS** (APCI-TOF): calculated for $C_{20}H_{26}NO_3S$ $[M+H]^+$: m/z 360.1628, found 360.1629.

Diethyl (3aR,6aS)-3a-methyl-6-methylene-1,3a,4,5,6,6a,7,9-octahydroazuleno[4,5-c]furan-8,8(3H)-dicarboxylate (3n)

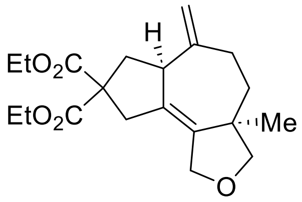
3n

Purified through flash column chromatography (15% EtOAc/Hexane) to obtain 20 mg (59% yield) of **3n** as a colourless oil. **^{1}H-NMR (300 MHz, $CDCl_3$)** δ (ppm): 4.87 (s, 1H), 4.79 (s, 1H), 4.31 – 4.11 (m, 4H), 3.75 – 3.53 (m, 4H), 3.23 (dd, J = 6.5, 1.4 Hz, 2H), 3.06 (s, 2H), 2.67 (t, J = 9.0 Hz, 1H), 2.55 –

2.38 (m, 2H), 1.88 – 1.78 (m, 2H), 1.25 (tq, *J* = 7.1, 2.0, 1.6 Hz, 6H), 1.12 (s, 3H). **^{13}C-NMR (75 MHz, $CDCl_3$)** δ (ppm): 171.9 (CO), 144.9 (C), 133.7 (C), 132.2 (C), 112.4 (CH_2), 82.3 (CH_2), 74.4 (CH_2), 61.5 (CH_2), 56.8 (C), 51.6 (CH), 48.1 (CH_2), 44.5 (C), 43.7 (CH_2), 35.8 (CH_2), 31.3 (CH_2), 26.1 (CH_3), 14.0 (CH_3). **HRMS** (APCI-TOF): calculated for $C_{20}H_{29}O_5$ $[M+H]^+$ *m/z* 349.2010 found 349.2016.

(3aR,6aS)-6a-Methyl-6-methylene-8-((4-nitrophenyl)sulfonyl)-1,3,3a,4,5,6,6a,7,8,9-decahydrofuro[3',4':3,4]cyclohepta[1,2-c]pyrrole (3o)

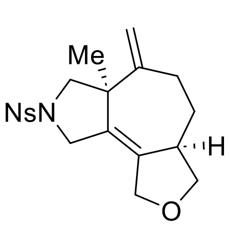

3o

Purified through flash column chromatography (25% Et_2O/Hexane) to obtain 18 mg (47% yield) of **3o** as a yellowish solid. **^{1}H-NMR (500 MHz, $CDCl_3$)** δ (ppm): 8.41 (d, *J* = 8.8 Hz, 2H), 8.03 (d, *J* = 8.8 Hz, 2H), 4.83 (s, 1H), 4.69 (s, 1H), 4.18 – 4.06 (m, 2H), 4.06 – 3.95 (m, 2H), 3.46 (dd, *J* = 13.2, 2.3 Hz, 1H), 3.39 (t, *J* = 8.8 Hz, 2H), 3.12 (d, *J* = 9.0 Hz, 1H), 2.85 (d, *J* = 9.5 Hz, 1H), 2.46 – 2.34 (m, 1H), 2.29 (ddd, *J* = 13.4, 7.6, 4.1 Hz, 1H), 1.90 (ddt, *J* = 12.7, 7.7, 4.6 Hz, 1H), 1.34 (s, 3H), 1.27 (d, *J* = 6.3 Hz, 1H). **^{13}C-NMR (126 MHz, $CDCl_3$)** δ (ppm): 149.3 (C), 148.9 (C), 140.7 (C), 133.9 (C), 127.9 (C), 127.8 (CH), 123.4 (CH), 109.6 (CH_2), 73.9 (CH_2), 69.8 (CH_2), 58.2 (CH_2), 50.0 (CH_2), 49.3 (C), 40.6 (CH), 32.6 (CH_2), 30.0 (CH_2), 23.4 (CH_3). **HRMS** (APCI-TOF): calculated for $C_{19}H_{23}N_2O_5S$ $[M+H]^+$ *m/z* 391.1322 found 391.1340.

4. Synthetic manipulations of cycloadduct 3b

(3a*R*,6*S*,6a*S*)-6-Methyl-8-tosyl-1,3,3a,4,5,6,6a,7,8,9 decahydrofuro[3',4':3,4]cyclohepta[1,2-c]pyrrole (6b)

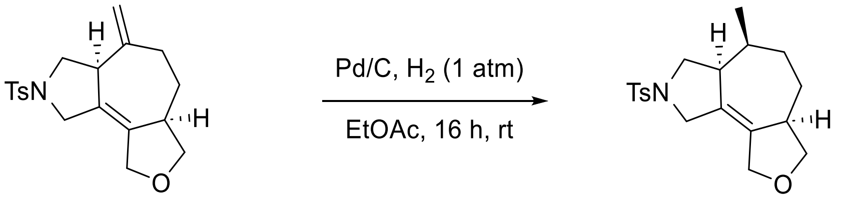

3b **6b**

To a solution of **3b** (34.4 mg, 0.1 mmol) in 2.5 mL of EtOAc was added Pd/C (10%, 10.6 mg). The reaction mixture was bubbled with a ballon of H_2 and stirred under hydrogen atmosphere for 16h at room temperature. After completion of the reaction, the mixture was quenched by adding 10 mL of H_2O and extracted with EtOAc (3×10 mL). The combined organic phase was washed with H_2O (3×10 mL), dried over anhydrous Na_2SO_4, concentrated in vacuo and purified by flash silica gel chromatography to afford 25 mg

of **6b** as a colourless oil (73% yield) as a single diastereoisomer.[5] **^{1}H-NMR (300 MHz, $CDCl_3$)** δ (ppm): 7.70 (d, J = 8.2 Hz, 2H), 7.33 (d, J = 7.9 Hz, 2H), 4.17 (d, J = 7.3 Hz, 1H), 4.11 (t, J = 8.3 Hz, 1H), 3.69 – 3.49 (m, 2H), 3.48 – 3.31 (m, 2H), 3.02 (q, J = 6.0 Hz, 2H), 2.65 (s, 1H), 2.44 (s, 3H), 1.94 – 1.68 (m, 3H), 1.41 (td, J = 7.3, 5.9, 3.2 Hz, 2H), 0.87 (d, J = 6.4 Hz, 1H), 0.78 (d, J = 6.7 Hz, 3H). **^{13}C-NMR (75 MHz, $CDCl_3$)** δ (ppm): 143.7 (C), 136.3 (C), 132.1 (C), 129.6 (CH), 128.0 (CH), 126.7 (C), 75.2 (CH_2), 70.5 (CH_2), 52.5 (CH_2), 52.1 (CH_2), 47.1 (CH), 44.7 (CH), 36.0 (CH_2), 31.7 (CH), 23.4 (CH_2), 21.5 (CH_3), 11.9 (CH_3). **HRMS** (APCI-TOF): calculated for $C_{19}H_{26}NO_3S$ $[M+H]^+$ *m/z* 348.4730 found 348.4731.

(3aR,6aS)-8-Tosyl-3,3a,4,5,6a,7,8,9-octahydrofuro[3',4':3,4]cyclohepta[1,2-c]pyrrol-6(1H)-one (5b)

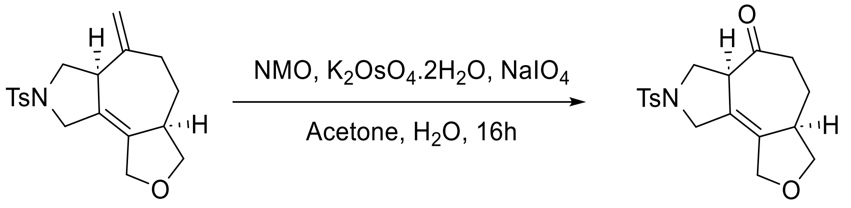

3b **5b**

To a solution of the diene **3b** (17.0 mg, 0.05 mmol) in acetone (0.6 mL) at rt NMO (25 mg, 0.215 mmol), H_2O (0.2 mL) and $K_2OsO_4\cdot 2H_2O$ (2.6 mg, 0.007 mmol) were sequentially added. The reaction mixture was then stirred at rt until complete conversion by TLC. At this moment, $NaIO_4$ (46 mg, 0.215 mmol) was added in a single portion. After stirring the resultant mixture at rt for 1h, the reaction mixture was filtered to remove any solids and the filtrate was diluted with EtOAc and H_2O. The filtrates were transfer to a separatory funnel and the layers were separated. The aqueous phase was extracted with EtOAc (x2). The combined organic layers were then washed with H_2O and brine before being dried ($MgSO_4$), filtered, and concentrated. The resultant residue was purified via column chromatography (silica gel, hexanes/EtOAc 50%) to afford **5b** as a colourless oil (11.5 mg, 63% yield).**^{1}H-NMR (300 MHz, $CDCl_3$)** δ (ppm): 7.73 (d, J = 8.0 Hz, 2H), 7.40 (d, J = 7.9 Hz, 2H), 4.17 (dd, J = 9.0, 6.0 Hz, 1H), 4.09 (s, 1H), 3.74 (d, J = 7.4 Hz, 3H), 3.66 (d, J = 11.3 Hz, 1H), 3.58 – 3.44 (m, 3H), 3.38 – 3.20 (m, 2H), 3.12 (s, 1H), 2.67 (d, J = 13.0 Hz, 1H), 2.48 (m, 4H). **^{13}C-NMR (75 MHz, $CDCl_3$)** δ (ppm): 206.7 (CO), 144.6 (C), 130.9 (C), 129.9 (CH), 128.2 (CH), 86.5 (C), 83.9 (C), 75.2 (CH_2), 73.2 (CH_2), 60.0 (CH), 59.7 (CH_2), 48.7 (CH), 48.3 (CH_2), 38.6 (CH_2), 23.2 (CH_2), 21.6 (CH_3). **HRMS** (APCI-TOF): calculated for $C_{18}H_{22}NO_4S$ $[M+H]^+$ *m/z* 347.1191 found 347.1195.

6. Computational details

All the calculations reported in this paper were performed with the Gaussian 09 suite of programs.[6] Electron correlation was partially taken into account using the hybrid functional usually denoted as B3LYP[7] in conjunction with the D3 dispersion correction suggested by Grimme et al.[8] using the standard double-[illegible] quality def2-SVP[9] basis set for all atoms. The Polarization Continuum Model (PCM)[10] was used to model the effects of the solvent (dichloroethane). This level is denoted PCM(dichloroethane)-B3LYP-D3/def2-SVP. Geometries were fully optimized in solution without any geometry or symmetry constraints. Reactants, intermediates, and products were characterized by frequency calculations,[11] and have positive definite Hessian matrices. Transition structures (TS's) show only one negative eigenvalue in their diagonalized force constant matrices, and their associated eigenvectors were confirmed to correspond to the motion along the reaction coordinate under consideration using the Intrinsic Reaction Coordinate (IRC) method.[12] This computational level was selected to enable a direct comparison with with the analogous process involving the cobalt(I)-catalyzed intramolecular (3+2) cycloadditions of ynilidenecyclopropanes, reported recently by us.[13] (see ref. 7a). In this previous study on the [3+2] cycloaddition we found that, although spin crossovers cannot be fully discarded, the transformation mainly occurs on the singlet hypersurface. For this reason, we only considered singlet species in the present mechanistic study.

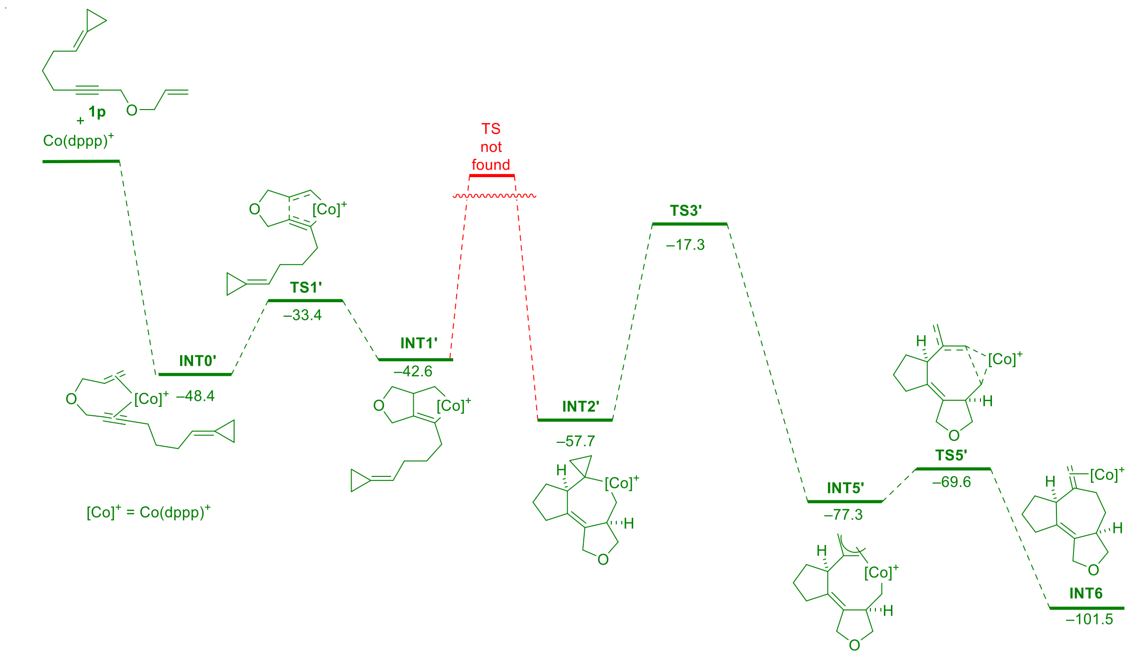

Figure S2. Pathway via the cobaltacyclic intermediate **INT1'**

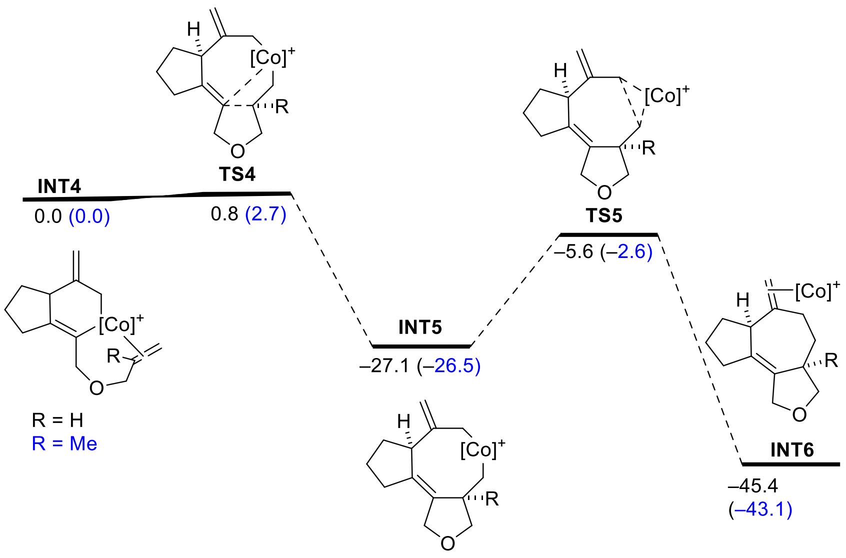

Figure S3. Comparison of the migratory insertion /reductive elimination steps for **1p** (R = H) and **1r** (R = Me, blue values).

7. Cartesian coordinates (in Å) and free energies (in a.u.)

All calculations have been performed at the PCM(dichloroethane)-B3LYP-D3/def2-SVP level.

1p, G = -580.660116

C	4.075019000	-0.538973000	-0.399891000
C	4.849832000	0.527482000	-0.236212000
C	5.944068000	1.373715000	-0.726007000
C	5.208599000	1.688586000	0.590195000
H	6.973837000	1.001075000	-0.660327000
H	5.762047000	2.032381000	-1.584267000
H	4.217005000	-1.162029000	-1.293050000
H	5.748265000	1.524082000	1.531044000
H	4.536626000	2.555529000	0.607117000
C	2.978498000	-0.953143000	0.543739000
H	3.174262000	-1.980274000	0.906238000
H	2.984769000	-0.298677000	1.432019000
C	1.589379000	-0.924575000	-0.111109000
H	1.587187000	-1.562150000	-1.012027000
H	1.363156000	0.099082000	-0.453683000
C	0.480911000	-1.399008000	0.846954000
H	0.482613000	-0.767470000	1.753740000
H	0.708231000	-2.422853000	1.195289000
C	-0.857278000	-1.380884000	0.256056000
C	-1.955149000	-1.360760000	-0.262311000
C	-3.295370000	-1.329683000	-0.869952000
H	-3.212837000	-1.500277000	-1.955722000
H	-3.903283000	-2.161629000	-0.457923000
O	-3.971357000	-0.096375000	-0.717621000
C	-4.367174000	0.195543000	0.603571000
H	-3.491718000	0.201771000	1.287287000
H	-5.045666000	-0.596055000	0.991904000

C	-5.057951000	1.523989000	0.677193000
H	-5.403003000	1.797338000	1.681909000
C	-5.262517000	2.347800000	-0.353665000
H	-4.922131000	2.085308000	-1.358509000
H	-5.773706000	3.305322000	-0.221429000

Co(dppp)$^{+}$, G = -3108.467343

Co	0.079705000	-0.965814000	-1.005874000
P	-1.451586000	-0.205069000	0.264441000
P	1.636652000	-0.343948000	0.294050000
C	1.428660000	-0.888601000	2.072454000
H	1.987338000	-1.829799000	2.178089000
H	1.922430000	-0.146040000	2.717020000
C	-0.994847000	0.017994000	2.041620000
H	-1.910419000	0.033857000	2.650470000
H	-0.535252000	1.014641000	2.138086000
C	-0.033508000	-1.092203000	2.503427000
H	-0.393271000	-2.064384000	2.127607000
H	-0.063675000	-1.159149000	3.602588000
C	3.228758000	-1.101519000	-0.217713000
C	4.426105000	-0.393841000	-0.402950000
C	3.194792000	-2.483480000	-0.486464000
C	5.577184000	-1.066281000	-0.828099000
H	4.467551000	0.680788000	-0.215417000
C	4.346054000	-3.151626000	-0.907316000
H	2.252988000	-3.029899000	-0.370247000
C	5.541149000	-2.442176000	-1.077047000
H	6.507289000	-0.509321000	-0.965586000
H	4.309945000	-4.224897000	-1.109272000
H	6.443276000	-2.961852000	-1.408961000
C	1.922666000	1.469762000	0.393486000
C	2.748677000	2.069983000	1.360995000
C	1.263216000	2.283542000	-0.542047000
C	2.905665000	3.458176000	1.387882000
H	3.280911000	1.460655000	2.095445000
C	1.416012000	3.672196000	-0.510745000
H	0.614157000	1.814665000	-1.283776000
C	2.237182000	4.261134000	0.455487000
H	3.550996000	3.915442000	2.141840000
H	0.885778000	4.292181000	-1.237594000
H	2.356467000	5.346970000	0.485647000
C	-2.265127000	1.358032000	-0.248789000
C	-2.987397000	2.170702000	0.639918000
C	-2.155556000	1.737046000	-1.597555000
C	-3.586618000	3.347726000	0.181627000
H	-3.089353000	1.893657000	1.691500000
C	-2.763047000	2.908849000	-2.055251000
H	-1.582451000	1.109129000	-2.286587000
C	-3.476375000	3.717585000	-1.163730000
H	-4.143343000	3.978655000	0.878798000
H	-2.672723000	3.194816000	-3.105912000
H	-3.946265000	4.638754000	-1.516880000
C	-2.795548000	-1.459468000	0.235174000
C	-3.329150000	-2.092190000	1.368367000
C	-3.257961000	-1.833502000	-1.040211000
C	-4.315241000	-3.074903000	1.223989000
H	-2.987912000	-1.831287000	2.371616000
C	-4.244566000	-2.810412000	-1.181780000
H	-2.845477000	-1.345730000	-1.929022000
C	-4.775368000	-3.434571000	-0.046409000

H	-4.726044000	-3.560234000	2.112693000
H	-4.599305000	-3.085777000	-2.177914000
H	-5.546488000	-4.201323000	-0.152935000

INT0, G = -3689.202859

C	-0.218336000	3.411316000	1.557511000
C	-0.521880000	2.215849000	0.740962000
C	1.329753000	0.704399000	2.267759000
C	0.961289000	1.865115000	3.159929000
C	0.993882000	3.235908000	2.478534000
C	-1.198457000	1.655225000	-0.200974000
C	-2.317584000	1.928815000	-1.142174000
H	-2.650385000	1.014597000	-1.662641000
C	0.680354000	-0.523536000	2.281390000
C	0.975076000	-1.890866000	2.776692000
Co	0.054882000	0.402936000	0.548651000
P	1.709528000	0.603520000	-0.861504000
C	-0.283391000	-1.146447000	3.225597000
H	1.807341000	-1.980671000	3.483174000
H	0.823813000	-2.769767000	2.148498000
H	2.377134000	0.706332000	1.940037000
H	-0.342586000	-0.750208000	4.245213000
H	-1.230023000	-1.539078000	2.840284000
H	1.685757000	1.855620000	3.994923000
H	-0.033402000	1.691055000	3.602810000
H	1.923366000	3.337376000	1.896098000
H	0.998961000	4.039652000	3.230521000
H	-1.121772000	3.616166000	2.160718000
H	-0.089479000	4.280896000	0.891370000
H	-1.962094000	2.635390000	-1.923638000
P	-0.768761000	-1.471796000	-0.530070000
C	-0.150011000	-1.582824000	-2.277860000
H	0.857865000	-2.022032000	-2.241338000
H	-0.773356000	-2.299542000	-2.829788000
C	1.105412000	0.662846000	-2.621851000
H	1.972003000	0.387113000	-3.240524000
H	0.881593000	1.713859000	-2.848549000
C	-0.106479000	-0.217573000	-2.976868000
H	-0.089136000	-0.379009000	-4.065664000
H	-1.037318000	0.329864000	-2.777161000
C	-0.533966000	-3.191729000	0.092940000
C	0.609123000	-3.940781000	-0.239807000
C	-1.469026000	-3.747493000	0.985489000
C	0.818492000	-5.204536000	0.318848000
H	1.359646000	-3.546350000	-0.924396000
C	-1.254911000	-5.009642000	1.545008000
H	-2.373685000	-3.197141000	1.250242000
C	-0.108479000	-5.740771000	1.217956000
H	1.714734000	-5.768724000	0.049795000
H	-1.990513000	-5.422540000	2.239382000
H	0.059063000	-6.726826000	1.657611000
C	-2.606730000	-1.358489000	-0.658657000
C	-3.319315000	-1.859845000	-1.760815000
C	-3.317454000	-0.742844000	0.387068000
C	-4.709343000	-1.725037000	-1.824296000
H	-2.802867000	-2.353476000	-2.585503000
C	-4.706644000	-0.615095000	0.325344000
H	-2.786938000	-0.330214000	1.247092000
C	-5.405179000	-1.098242000	-0.785045000
H	-5.248861000	-2.107822000	-2.693901000

H	-5.239323000	-0.111237000	1.134348000
H	-6.490187000	-0.983192000	-0.842440000
C	2.602216000	2.191023000	-0.629219000
C	3.846389000	2.221359000	0.023600000
C	1.997416000	3.401606000	-1.008869000
C	4.472838000	3.442479000	0.288842000
H	4.333631000	1.293537000	0.328520000
C	2.629662000	4.618596000	-0.748684000
H	1.016320000	3.406509000	-1.487512000
C	3.867381000	4.642361000	-0.095738000
H	5.438854000	3.453013000	0.798831000
H	2.148257000	5.552010000	-1.049033000
H	4.357772000	5.595841000	0.113795000
C	3.041687000	-0.656090000	-0.873466000
C	3.061933000	-1.669141000	0.092385000
C	4.068554000	-0.599470000	-1.836206000
C	4.080950000	-2.627886000	0.092739000
H	2.274060000	-1.711727000	0.838814000
C	5.083310000	-1.556844000	-1.833705000
H	4.091886000	0.197749000	-2.582941000
C	5.090080000	-2.575173000	-0.870746000
H	4.076621000	-3.417570000	0.847538000
H	5.875273000	-1.506875000	-2.584584000
H	5.886077000	-3.323662000	-0.873712000
O	-3.386346000	2.480074000	-0.403576000
C	-4.571914000	2.636862000	-1.149406000
H	-4.841096000	1.684239000	-1.655170000
H	-4.432853000	3.385336000	-1.959546000
C	-5.709201000	3.052001000	-0.265688000
H	-6.646974000	3.250343000	-0.798599000
C	-5.647549000	3.173711000	1.062866000
H	-4.717265000	2.976297000	1.601403000
H	-6.522158000	3.474421000	1.645969000

TS1, G = -3689.180414

C	1.926465000	-3.034632000	1.605287000
C	1.234830000	-1.902461000	0.890440000
C	-0.195907000	-1.515795000	2.146590000
C	0.650689000	-1.929657000	3.341677000
C	1.309423000	-3.257526000	2.990283000
C	1.511729000	-1.288442000	-0.254618000
C	2.659841000	-1.432254000	-1.184394000
H	2.779262000	-0.520694000	-1.798511000
C	-0.734546000	-0.154113000	2.060867000
C	-1.851516000	0.447123000	2.836263000
Co	0.024388000	-0.342015000	0.301162000
P	-1.753916000	-0.754205000	-0.886206000
C	-0.429793000	0.938656000	3.049567000
H	-2.319314000	-0.149816000	3.628570000
H	-2.535077000	1.138138000	2.335873000
H	-0.942721000	-2.291314000	1.919136000
H	0.072489000	0.673061000	3.985803000
H	-0.186141000	1.938496000	2.680239000
H	-0.003906000	-1.998485000	4.226607000
H	1.410582000	-1.159570000	3.548909000
H	0.545396000	-4.050994000	2.951970000
H	2.062767000	-3.562487000	3.731019000
H	2.991794000	-2.761451000	1.661313000
H	1.884903000	-3.933340000	0.966879000
H	2.431057000	-2.261020000	-1.891009000

P	0.420934000	1.589171000	-0.626334000
C	-0.309072000	1.750563000	-2.317221000
H	-1.341401000	2.098441000	-2.174597000
H	0.205763000	2.561402000	-2.850681000
C	-1.258821000	-0.658638000	-2.678941000
H	-2.190954000	-0.597635000	-3.261027000
H	-0.832186000	-1.650395000	-2.884823000
C	-0.248130000	0.430892000	-3.100127000
H	-0.412579000	0.646621000	-4.167169000
H	0.771492000	0.022167000	-3.031405000
C	-0.212842000	3.056971000	0.277147000
C	-1.593769000	3.139068000	0.532220000
C	0.635361000	4.068583000	0.754924000
C	-2.117393000	4.218164000	1.244989000
H	-2.265052000	2.352737000	0.188366000
C	0.106488000	5.145537000	1.474958000
H	1.709974000	4.020722000	0.571238000
C	-1.267311000	5.223020000	1.721863000
H	-3.192061000	4.266881000	1.435576000
H	0.775227000	5.926828000	1.844133000
H	-1.675845000	6.064114000	2.287145000
C	2.229285000	1.875829000	-0.790789000
C	2.826364000	2.386465000	-1.954072000
C	3.047884000	1.506946000	0.292633000
C	4.218131000	2.507731000	-2.036440000
H	2.223744000	2.686052000	-2.812561000
C	4.434246000	1.631724000	0.208904000
H	2.601053000	1.090761000	1.197900000
C	5.023726000	2.125345000	-0.960235000
H	4.671488000	2.895550000	-2.951689000
H	5.058009000	1.316874000	1.047671000
H	6.110912000	2.205496000	-1.032640000
C	-2.307202000	-2.510536000	-0.735205000
C	-3.663807000	-2.869632000	-0.822779000
C	-1.341230000	-3.525173000	-0.608625000
C	-4.042199000	-4.214760000	-0.766910000
H	-4.434497000	-2.107643000	-0.941798000
C	-1.723691000	-4.866987000	-0.556836000
H	-0.283061000	-3.269026000	-0.551859000
C	-3.076560000	-5.215609000	-0.629389000
H	-5.100566000	-4.477647000	-0.834311000
H	-0.959904000	-5.641584000	-0.455698000
H	-3.376715000	-6.265136000	-0.582252000
C	-3.300285000	0.213155000	-0.718953000
C	-4.026801000	0.096567000	0.481266000
C	-3.765244000	1.095234000	-1.709601000
C	-5.170127000	0.866980000	0.697324000
H	-3.703648000	-0.610181000	1.246112000
C	-4.906692000	1.871847000	-1.487282000
H	-3.250008000	1.191452000	-2.665690000
C	-5.605827000	1.767347000	-0.281597000
H	-5.720887000	0.764655000	1.635121000
H	-5.249678000	2.558592000	-2.264373000
H	-6.495249000	2.377660000	-0.108768000
O	3.845970000	-1.686525000	-0.460153000
C	5.016123000	-1.675934000	-1.241718000
H	5.065720000	-0.748540000	-1.853370000
H	5.017732000	-2.519556000	-1.965687000
C	6.238615000	-1.748883000	-0.376913000
H	7.181034000	-1.837225000	-0.930837000
C	6.243180000	-1.699962000	0.957917000

H	5.310697000	-1.605870000	1.520331000
H	7.177165000	-1.751487000	1.523961000

INT1, G = -3689.208798

C	1.076199000	4.204724000	0.434813000
C	1.003297000	2.689901000	0.406978000
C	1.916375000	2.125669000	1.460476000
C	1.997336000	3.267828000	2.493159000
C	2.075079000	4.507335000	1.579116000
C	0.213578000	1.847159000	-0.288648000
C	-0.693075000	2.305120000	-1.415740000
H	-1.385533000	1.504711000	-1.721902000
C	1.419445000	0.743372000	1.800891000
C	2.145449000	-0.151509000	2.746948000
Co	0.145741000	0.128223000	0.512051000
P	1.598451000	-0.647125000	-0.851618000
C	0.786207000	0.404645000	3.141404000
H	3.035210000	0.254346000	3.241580000
H	2.200114000	-1.226795000	2.546657000
H	2.923253000	2.028929000	1.012216000
H	0.721978000	1.186684000	3.904259000
H	-0.031864000	-0.324392000	3.198141000
H	2.849296000	3.177810000	3.184105000
H	1.071989000	3.299523000	3.093246000
H	3.097202000	4.593925000	1.173708000
H	1.849612000	5.447412000	2.105103000
H	0.078191000	4.618485000	0.645756000
H	1.379009000	4.626099000	-0.538391000
H	-0.084535000	2.564289000	-2.300136000
P	-1.655980000	-1.099320000	-0.473486000
C	-1.090493000	-2.188285000	-1.869546000
H	-0.558231000	-2.997084000	-1.348744000
H	-1.970579000	-2.646037000	-2.345476000
C	0.942291000	-0.605669000	-2.581974000
H	1.799921000	-0.724068000	-3.260599000
H	0.584996000	0.423697000	-2.713675000
C	-0.166000000	-1.612466000	-2.964691000
H	0.306057000	-2.483661000	-3.443529000
H	-0.771461000	-1.136325000	-3.750371000
C	-2.200083000	-2.312284000	0.805286000
C	-2.547925000	-3.639727000	0.499412000
C	-2.270531000	-1.884115000	2.143372000
C	-2.942417000	-4.517221000	1.513171000
H	-2.520007000	-4.005320000	-0.527784000
C	-2.674820000	-2.760543000	3.154460000
H	-2.022006000	-0.853217000	2.404782000
C	-3.004655000	-4.082252000	2.841495000
H	-3.205925000	-5.547163000	1.260997000
H	-2.725296000	-2.408483000	4.187351000
H	-3.312355000	-4.772559000	3.630451000
C	-3.225061000	-0.317471000	-1.024523000
C	-3.459554000	-0.033996000	-2.380731000
C	-4.171749000	0.088895000	-0.068001000
C	-4.615008000	0.650976000	-2.769932000
H	-2.744757000	-0.337647000	-3.147132000
C	-5.327206000	0.767484000	-0.461495000
H	-4.013564000	-0.121857000	0.991504000
C	-5.549918000	1.055594000	-1.812746000
H	-4.782637000	0.866304000	-3.827842000
H	-6.054470000	1.076757000	0.292861000

H	-6.451046000	1.592295000	-2.118385000
C	3.195749000	0.233180000	-0.934915000
C	4.251877000	-0.158471000	-0.095392000
C	3.338876000	1.374391000	-1.741642000
C	5.436204000	0.582281000	-0.065681000
H	4.149935000	-1.037894000	0.542999000
C	4.525609000	2.110457000	-1.709391000
H	2.524266000	1.710029000	-2.385126000
C	5.574284000	1.718346000	-0.869806000
H	6.251660000	0.271428000	0.591497000
H	4.628404000	2.997163000	-2.339098000
H	6.499421000	2.298982000	-0.842717000
C	2.027690000	-2.397297000	-0.521304000
C	1.392204000	-3.089279000	0.521366000
C	2.949690000	-3.072867000	-1.339547000
C	1.667752000	-4.441075000	0.744974000
H	0.660323000	-2.585110000	1.154605000
C	3.224363000	-4.423432000	-1.113493000
H	3.457341000	-2.546698000	-2.151912000
C	2.584381000	-5.108367000	-0.072834000
H	1.162035000	-4.969471000	1.556100000
H	3.940513000	-4.944441000	-1.753131000
H	2.801552000	-6.165252000	0.099014000
O	-1.417139000	3.481211000	-1.102991000
C	-2.454043000	3.285644000	-0.152657000
H	-2.080959000	2.729590000	0.729982000
H	-3.261031000	2.675776000	-0.605146000
C	-2.986288000	4.623908000	0.258920000
H	-3.327813000	5.265112000	-0.563103000
C	-3.065303000	5.050720000	1.522283000
H	-2.719667000	4.430766000	2.356924000
H	-3.482290000	6.030507000	1.772457000

TS2, G = -3689.165864

C	-1.508436000	-1.815003000	3.293101000
C	-0.339333000	-1.448226000	2.384029000
C	0.471231000	-2.726590000	2.121072000
C	-0.548402000	-3.859757000	2.342882000
C	-1.363744000	-3.334984000	3.538909000
C	-0.023496000	-0.264030000	1.778180000
C	-0.479936000	1.092436000	2.200012000
H	-0.726532000	1.708988000	1.318867000
C	1.215286000	-2.598566000	0.805687000
C	2.275610000	-3.425955000	0.491210000
Co	0.368864000	-0.996449000	0.059039000
P	1.989665000	0.009825000	-0.965875000
C	0.545699000	-2.922090000	-0.571044000
H	2.520681000	-4.294685000	1.118304000
H	2.933773000	-3.244853000	-0.360234000
H	1.234471000	-2.798992000	2.920028000
H	-0.249426000	-3.675472000	-0.526066000
H	1.149430000	-3.020982000	-1.476895000
H	-0.075350000	-4.835190000	2.529439000
H	-1.197939000	-3.966089000	1.458614000
H	-0.796694000	-3.510810000	4.467950000
H	-2.333123000	-3.844051000	3.648561000
H	-2.460241000	-1.568217000	2.796211000
H	-1.496156000	-1.220267000	4.217203000
H	0.394466000	1.588693000	2.676233000
P	-1.193368000	-0.202167000	-1.369605000

C	-0.493832000	0.178743000	-3.049273000
H	-1.302106000	0.125227000	-3.791827000
H	-0.174314000	1.229807000	-3.031395000
C	2.030466000	-0.463552000	-2.765220000
H	2.658326000	-1.364837000	-2.822757000
H	2.574567000	0.316009000	-3.318149000
C	0.665797000	-0.753941000	-3.432281000
H	0.368798000	-1.794294000	-3.228383000
H	0.815922000	-0.696463000	-4.521644000
C	-2.463170000	-1.511834000	-1.620030000
C	-2.809461000	-2.288207000	-0.499427000
C	-3.117405000	-1.747124000	-2.838716000
C	-3.789989000	-3.277669000	-0.591712000
H	-2.304927000	-2.110819000	0.452603000
C	-4.090375000	-2.748237000	-2.934254000
H	-2.882693000	-1.155172000	-3.725046000
C	-4.429955000	-3.513111000	-1.813881000
H	-4.049409000	-3.867412000	0.290788000
H	-4.587636000	-2.926787000	-3.890723000
H	-5.192597000	-4.291628000	-1.892724000
C	-2.204184000	1.296136000	-0.991249000
C	-3.550684000	1.193037000	-0.602290000
C	-1.619644000	2.573795000	-1.077245000
C	-4.302514000	2.343171000	-0.340064000
H	-4.027459000	0.215464000	-0.515417000
C	-2.375233000	3.719147000	-0.820004000
H	-0.569839000	2.691107000	-1.343990000
C	-3.721760000	3.609108000	-0.458272000
H	-5.350400000	2.244933000	-0.046331000
H	-1.902382000	4.700852000	-0.897710000
H	-4.311840000	4.506130000	-0.255987000
C	1.937881000	1.852092000	-0.926714000
C	1.882145000	2.457286000	0.339634000
C	1.926124000	2.666736000	-2.070183000
C	1.774975000	3.842072000	0.464701000
H	1.918143000	1.832929000	1.232115000
C	1.820470000	4.057036000	-1.944348000
H	1.988262000	2.239046000	-3.071195000
C	1.732584000	4.646669000	-0.680365000
H	1.712921000	4.292675000	1.457909000
H	1.800890000	4.677876000	-2.842996000
H	1.636887000	5.730842000	-0.586378000
C	3.676998000	-0.341365000	-0.325464000
C	4.805032000	-0.363506000	-1.162669000
C	3.838169000	-0.533668000	1.057060000
C	6.074777000	-0.586924000	-0.620594000
H	4.710176000	-0.202487000	-2.238345000
C	5.109629000	-0.744899000	1.595063000
H	2.957778000	-0.530932000	1.703918000
C	6.229434000	-0.776899000	0.756554000
H	6.946468000	-0.609269000	-1.279051000
H	5.224771000	-0.893371000	2.671439000
H	7.223214000	-0.950840000	1.176298000
O	-1.564087000	1.059347000	3.112364000
C	-1.805836000	2.301349000	3.756467000
H	-2.632356000	2.106377000	4.460656000
H	-0.922087000	2.611601000	4.351638000
C	-2.194374000	3.390976000	2.795738000
H	-3.016891000	3.138779000	2.117038000
C	-1.599053000	4.583524000	2.712018000
H	-0.758038000	4.850111000	3.362161000

H	-1.929510000	5.337897000	1.992400000

INT2, G = -3689.244185

C	1.289252000	0.402373000	-3.870408000
C	0.651040000	-0.342359000	-2.707035000
C	0.523331000	-1.802954000	-3.088271000
C	1.772174000	-2.016422000	-3.958571000
C	1.797695000	-0.727731000	-4.809069000
C	0.431166000	0.080516000	-1.454138000
C	0.611356000	1.527517000	-1.089983000
H	0.210785000	1.747392000	-0.089226000
C	0.302727000	-2.601003000	-1.822231000
C	-1.005516000	-2.674705000	-1.305307000
Co	0.093301000	-1.329093000	-0.172225000
P	-1.805810000	-0.653825000	0.967733000
C	1.351644000	-2.819056000	-0.907484000
H	-1.858665000	-2.345304000	-1.902922000
H	-1.243246000	-3.411355000	-0.528748000
H	-0.364935000	-1.928649000	-3.734224000
H	2.388050000	-2.648162000	-1.193525000
H	1.212149000	-3.550089000	-0.098606000
H	1.731076000	-2.935609000	-4.562610000
H	2.671757000	-2.071328000	-3.323127000
H	1.108045000	-0.842655000	-5.660962000
H	2.793706000	-0.517865000	-5.226660000
H	2.108596000	1.042831000	-3.502576000
H	0.577714000	1.077939000	-4.368231000
H	1.696799000	1.763435000	-1.047274000
P	1.578613000	-0.569488000	1.401736000
C	1.086989000	-1.444299000	2.982040000
H	1.813162000	-2.258021000	3.123193000
H	1.230257000	-0.735236000	3.811389000
C	-1.507643000	-1.097739000	2.767554000
H	-2.439928000	-1.533516000	3.152562000
H	-1.357937000	-0.168938000	3.333429000
C	-0.328797000	-2.053225000	3.027458000
H	-0.382829000	-2.927909000	2.358951000
H	-0.458604000	-2.464237000	4.041511000
C	3.340507000	-0.969116000	1.087240000
C	3.831571000	-0.760590000	-0.214868000
C	4.224503000	-1.381912000	2.096140000
C	5.180894000	-0.974239000	-0.504412000
H	3.153424000	-0.428893000	-1.004977000
C	5.572988000	-1.606908000	1.799459000
H	3.878116000	-1.522064000	3.122083000
C	6.053052000	-1.404843000	0.501763000
H	5.549486000	-0.808294000	-1.519396000
H	6.251558000	-1.935068000	2.590410000
H	7.107548000	-1.579301000	0.274991000
C	1.677090000	1.217462000	1.874067000
C	2.772353000	2.011783000	1.491876000
C	0.607135000	1.832958000	2.545016000
C	2.779782000	3.384710000	1.754395000
H	3.624888000	1.570091000	0.975688000
C	0.613001000	3.204615000	2.804752000
H	-0.256281000	1.255776000	2.862156000
C	1.698267000	3.988835000	2.402330000
H	3.638970000	3.984583000	1.445040000
H	-0.240815000	3.657099000	3.314165000
H	1.703483000	5.063743000	2.597409000

C	-2.440694000	1.064436000	0.953704000
C	-2.432392000	1.768385000	-0.261656000
C	-2.973802000	1.680392000	2.100084000
C	-2.918128000	3.077349000	-0.324444000
H	-2.027447000	1.315293000	-1.166682000
C	-3.454190000	2.990487000	2.034072000
H	-3.022528000	1.150163000	3.052781000
C	-3.421903000	3.693536000	0.824293000
H	-2.881539000	3.613840000	-1.275059000
H	-3.857590000	3.462167000	2.933251000
H	-3.792230000	4.720487000	0.778496000
C	-3.295366000	-1.658597000	0.539619000
C	-3.449421000	-2.955031000	1.062148000
C	-4.241198000	-1.180706000	-0.382169000
C	-4.531646000	-3.751372000	0.676940000
H	-2.725408000	-3.364744000	1.769848000
C	-5.321794000	-1.980534000	-0.765635000
H	-4.147031000	-0.178172000	-0.802780000
C	-5.471263000	-3.266918000	-0.238164000
H	-4.637589000	-4.755137000	1.095331000
H	-6.052134000	-1.591339000	-1.479142000
H	-6.317128000	-3.889724000	-0.538677000
O	-0.019192000	2.358021000	-2.048447000
C	0.160911000	3.730906000	-1.799928000
H	1.239498000	3.999184000	-1.833977000
H	-0.179705000	3.991942000	-0.774194000
C	-0.592517000	4.560474000	-2.794738000
H	-0.470572000	5.642934000	-2.667323000
C	-1.367371000	4.079233000	-3.771214000
H	-1.496161000	3.002272000	-3.907178000
H	-1.891126000	4.747216000	-4.460321000

TS3, G = -3689.212000

C	-1.819999000	0.117794000	3.365800000
C	-1.215750000	0.872554000	2.211835000
C	-2.212478000	1.933781000	1.746118000
C	-2.763724000	2.407517000	3.106052000
C	-2.956890000	1.069328000	3.861540000
C	0.070697000	1.005251000	1.809851000
C	1.265380000	0.456356000	2.538967000
H	2.197128000	0.637757000	1.977387000
C	-1.378667000	2.807820000	0.831975000
C	-1.661439000	3.007687000	-0.514122000
Co	-0.277608000	1.333163000	-0.079276000
P	-1.401686000	-0.016369000	-1.432621000
C	-0.003516000	2.949986000	1.278850000
H	-2.653681000	2.770554000	-0.896395000
H	-1.063831000	3.702082000	-1.110172000
H	-3.045900000	1.495150000	1.173340000
H	0.222465000	3.184987000	2.321222000
H	0.675376000	3.479415000	0.598188000
H	-3.693208000	2.990132000	3.019143000
H	-2.013267000	3.035808000	3.614640000
H	-3.939716000	0.643632000	3.605005000
H	-2.934671000	1.201412000	4.952928000
H	-1.056321000	-0.092627000	4.129449000
H	-2.216993000	-0.860864000	3.048670000
H	1.375432000	0.982664000	3.511639000
P	1.598734000	0.766819000	-1.137295000
C	1.407491000	1.212007000	-2.953350000

H	1.944518000	2.165196000	-3.065106000
H	1.958104000	0.470674000	-3.551871000
C	-0.972320000	0.202027000	-3.236114000
H	-1.904257000	0.334110000	-3.802628000
H	-0.528017000	-0.738858000	-3.591110000
C	-0.026542000	1.386459000	-3.475534000
H	-0.466985000	2.295251000	-3.037399000
H	0.035287000	1.566265000	-4.561387000
C	3.191650000	1.612237000	-0.723032000
C	3.326118000	2.500968000	0.351920000
C	4.315562000	1.346929000	-1.529773000
C	4.554197000	3.119943000	0.616088000
H	2.479588000	2.714218000	0.999723000
C	5.537588000	1.967983000	-1.269490000
H	4.240963000	0.643537000	-2.362467000
C	5.660248000	2.857438000	-0.194410000
H	4.640280000	3.807522000	1.460751000
H	6.399793000	1.753268000	-1.905482000
H	6.618679000	3.340475000	0.010614000
C	2.163226000	-0.993293000	-1.138029000
C	2.849569000	-1.469897000	-0.007898000
C	1.875488000	-1.891447000	-2.176885000
C	3.222922000	-2.810276000	0.089205000
H	3.108073000	-0.786589000	0.801014000
C	2.248049000	-3.235323000	-2.079983000
H	1.348678000	-1.562864000	-3.072393000
C	2.917091000	-3.700411000	-0.945570000
H	3.753177000	-3.160959000	0.977983000
H	2.004837000	-3.920514000	-2.895041000
H	3.200599000	-4.752579000	-0.867547000
C	-1.180797000	-1.806712000	-1.088713000
C	-0.545884000	-2.237001000	0.083768000
C	-1.672476000	-2.754577000	-2.003805000
C	-0.384772000	-3.604286000	0.327597000
H	-0.150527000	-1.516432000	0.799153000
C	-1.511348000	-4.118349000	-1.753835000
H	-2.186676000	-2.433872000	-2.913486000
C	-0.863032000	-4.544445000	-0.588186000
H	0.132891000	-3.925628000	1.232965000
H	-1.891716000	-4.850044000	-2.470839000
H	-0.728245000	-5.612101000	-0.397700000
C	-3.232552000	0.153016000	-1.315663000
C	-3.965404000	0.998277000	-2.165409000
C	-3.903176000	-0.522911000	-0.280539000
C	-5.343016000	1.158085000	-1.986555000
H	-3.471763000	1.547884000	-2.970040000
C	-5.278412000	-0.355522000	-0.100426000
H	-3.351463000	-1.184656000	0.390658000
C	-6.002217000	0.484432000	-0.953414000
H	-5.901181000	1.814489000	-2.658297000
H	-5.785547000	-0.887365000	0.708115000
H	-7.078275000	0.612588000	-0.814352000
O	1.059966000	-0.923533000	2.752929000
C	2.057270000	-1.551061000	3.528929000
H	2.303928000	-0.930985000	4.417053000
H	3.002083000	-1.644827000	2.954729000
C	1.605364000	-2.904667000	3.988460000
H	2.388522000	-3.505907000	4.465319000
C	0.362546000	-3.381005000	3.875354000
H	-0.426396000	-2.789240000	3.405244000
H	0.101936000	-4.376197000	4.245080000

INT3, G = -3689.264361

C	-0.079595000	3.811184000	1.715958000
C	-0.181029000	2.319943000	1.830417000
C	1.196231000	1.727488000	1.997774000
C	1.831049000	2.763995000	2.948960000
C	1.324029000	4.106107000	2.350475000
C	-1.164114000	1.453302000	2.128556000
C	-2.637215000	1.723529000	2.128715000
H	-3.199384000	0.767840000	2.071189000
C	0.908075000	0.299316000	2.402913000
C	1.770687000	-0.776143000	2.569788000
Co	0.514543000	-0.989254000	0.938638000
P	2.019747000	-0.857233000	-0.629169000
C	-0.579867000	0.122900000	2.542092000
H	2.846328000	-0.635931000	2.447862000
H	1.467561000	-1.653959000	3.154881000
H	1.764024000	1.756758000	1.051806000
H	-0.924929000	-0.250828000	3.522569000
H	-0.971936000	-0.731335000	1.859298000
H	2.928823000	2.701928000	2.992155000
H	1.438502000	2.617262000	3.968716000
H	2.021567000	4.444536000	1.568250000
H	1.278514000	4.902529000	3.107543000
H	-0.910527000	4.312007000	2.234336000
H	-0.119312000	4.139658000	0.664328000
H	-2.945712000	2.206167000	3.081690000
P	-0.876041000	-2.094308000	-0.428663000
C	-0.089939000	-3.561737000	-1.268604000
H	-0.491156000	-4.442137000	-0.746230000
H	-0.458638000	-3.618980000	-2.303897000
C	2.133039000	-2.333448000	-1.776333000
H	3.198280000	-2.526981000	-1.966831000
H	1.686722000	-2.037542000	-2.738037000
C	1.445237000	-3.588942000	-1.220930000
H	1.775731000	-3.768011000	-0.183552000
H	1.782757000	-4.460918000	-1.804549000
C	-2.325604000	-2.811748000	0.440975000
C	-2.141723000	-3.256485000	1.762362000
C	-3.579964000	-2.962361000	-0.171461000
C	-3.197796000	-3.847898000	2.460486000
H	-1.168973000	-3.134869000	2.249252000
C	-4.635496000	-3.549437000	0.532714000
H	-3.737477000	-2.616096000	-1.195229000
C	-4.446698000	-3.992891000	1.846429000
H	-3.046409000	-4.189642000	3.487157000
H	-5.610394000	-3.660658000	0.051826000
H	-5.275175000	-4.449233000	2.393572000
C	-1.583903000	-1.005506000	-1.724056000
C	-2.383855000	0.068264000	-1.289209000
C	-1.273879000	-1.114107000	-3.087545000
C	-2.877499000	1.000796000	-2.199862000
H	-2.609552000	0.198347000	-0.229655000
C	-1.763543000	-0.171787000	-3.999284000
H	-0.645198000	-1.924075000	-3.460538000
C	-2.566061000	0.883233000	-3.559651000
H	-3.479744000	1.836748000	-1.839592000
H	-1.508561000	-0.265212000	-5.057474000
H	-2.939777000	1.621842000	-4.272783000
C	1.705458000	0.563001000	-1.751062000

C	0.654333000	1.451770000	-1.481972000
C	2.499726000	0.761206000	-2.895139000
C	0.394373000	2.521355000	-2.343040000
H	0.020558000	1.303574000	-0.607614000
C	2.239608000	1.832259000	-3.752623000
H	3.328242000	0.083478000	-3.116794000
C	1.186419000	2.714196000	-3.477699000
H	-0.438577000	3.193534000	-2.126712000
H	2.861071000	1.979874000	-4.639196000
H	0.983068000	3.549085000	-4.152757000
C	3.747013000	-0.601112000	-0.057511000
C	4.484749000	-1.699777000	0.418281000
C	4.301079000	0.686215000	0.032494000
C	5.756891000	-1.513565000	0.964096000
H	4.066436000	-2.708907000	0.376139000
C	5.573168000	0.869790000	0.585030000
H	3.747987000	1.551342000	-0.338550000
C	6.303174000	-0.227501000	1.051154000
H	6.322148000	-2.375717000	1.326125000
H	5.994753000	1.876040000	0.645945000
H	7.297281000	-0.082497000	1.480738000
O	-2.947341000	2.558181000	1.033241000
C	-4.303227000	2.924028000	0.940964000
H	-4.665893000	3.345810000	1.903662000
H	-4.938251000	2.032312000	0.744865000
C	-4.507786000	3.934858000	-0.148078000
H	-5.553015000	4.224899000	-0.308250000
C	-3.534768000	4.475926000	-0.886783000
H	-2.490660000	4.191795000	-0.732111000
H	-3.755297000	5.212391000	-1.663967000

INT4, G = -3689.216749

C	2.140302000	-3.703831000	-1.392304000
C	0.990171000	-2.709133000	-1.552803000
C	-0.300759000	-3.497121000	-1.437578000
C	0.077866000	-4.638421000	-0.481070000
C	1.480266000	-5.036289000	-0.967154000
C	1.203594000	-1.382150000	-1.706674000
C	2.580331000	-0.900744000	-2.098499000
H	3.339815000	-1.273907000	-1.398495000
C	-1.537977000	-2.682601000	-1.164688000
C	-1.600276000	-1.398171000	-1.886853000
Co	-0.208733000	-0.238204000	-1.019191000
P	-1.869109000	0.718384000	0.366945000
C	-2.512222000	-3.120514000	-0.341889000
H	-1.297429000	-1.513058000	-2.937528000
H	-2.596749000	-0.940715000	-1.850757000
H	-0.447257000	-3.956090000	-2.439922000
H	-2.445536000	-4.073945000	0.185696000
H	-3.410987000	-2.527055000	-0.166726000
H	-0.637447000	-5.473697000	-0.500369000
H	0.115093000	-4.249220000	0.551926000
H	1.388837000	-5.708817000	-1.835283000
H	2.064764000	-5.568429000	-0.202126000
H	2.842487000	-3.340589000	-0.625513000
H	2.722112000	-3.793704000	-2.325997000
H	2.838565000	-1.301203000	-3.101222000
P	1.466950000	0.882997000	0.540321000
C	0.701197000	1.091047000	2.231246000
H	0.279293000	0.115601000	2.505752000

H	1.509218000	1.291701000	2.949858000
C	-1.326695000	2.326520000	1.127692000
H	-2.226740000	2.882174000	1.428748000
H	-0.854474000	2.905892000	0.319857000
C	-0.368978000	2.190208000	2.325757000
H	-0.958557000	2.005531000	3.237238000
H	0.122533000	3.166075000	2.463640000
C	2.822976000	-0.285125000	0.969125000
C	4.172738000	-0.045986000	0.664354000
C	2.467296000	-1.509999000	1.562040000
C	5.147375000	-1.000858000	0.970814000
H	4.465540000	0.875745000	0.160705000
C	3.442906000	-2.462163000	1.867998000
H	1.422055000	-1.743402000	1.775611000
C	4.787573000	-2.208829000	1.576385000
H	6.193196000	-0.799628000	0.726428000
H	3.147756000	-3.408719000	2.326935000
H	5.550525000	-2.954292000	1.812682000
C	2.248062000	2.548972000	0.388546000
C	3.222506000	2.963998000	1.316599000
C	1.793587000	3.475814000	-0.560482000
C	3.745200000	4.258216000	1.269890000
H	3.582393000	2.276226000	2.084546000
C	2.310790000	4.774666000	-0.605044000
H	1.021329000	3.197402000	-1.273827000
C	3.293354000	5.167336000	0.306434000
H	4.506212000	4.558671000	1.994124000
H	1.940707000	5.477488000	-1.355095000
H	3.702069000	6.180051000	0.272326000
C	-3.452585000	1.168547000	-0.448334000
C	-4.538967000	0.276497000	-0.457804000
C	-3.573142000	2.392411000	-1.131572000
C	-5.715625000	0.597301000	-1.139699000
H	-4.478380000	-0.667808000	0.084941000
C	-4.751967000	2.710949000	-1.811660000
H	-2.752719000	3.112271000	-1.138395000
C	-5.824517000	1.813678000	-1.820846000
H	-6.550971000	-0.106826000	-1.133287000
H	-4.830825000	3.667057000	-2.334432000
H	-6.744998000	2.064360000	-2.353322000
C	-2.374812000	-0.274568000	1.828908000
C	-1.690382000	-1.454740000	2.155943000
C	-3.399227000	0.183274000	2.676315000
C	-2.019206000	-2.167679000	3.312318000
H	-0.905261000	-1.829698000	1.499519000
C	-3.729909000	-0.532179000	3.829466000
H	-3.945023000	1.098634000	2.434984000
C	-3.039912000	-1.707860000	4.149244000
H	-1.478819000	-3.085816000	3.553935000
H	-4.527848000	-0.169303000	4.481518000
H	-3.299882000	-2.265072000	5.052492000
O	2.683992000	0.505550000	-2.100402000
C	1.786761000	1.060688000	-3.043561000
H	2.145984000	0.850154000	-4.068980000
H	1.805722000	2.146799000	-2.895286000
C	0.365674000	0.519020000	-2.937660000
H	0.108258000	-0.237458000	-3.682909000
C	-0.690921000	1.231521000	-2.362127000
H	-0.512556000	2.223067000	-1.938991000
H	-1.710431000	1.069726000	-2.717271000

TS4, G = -3689.215540

C	-2.477172000	3.461882000	-1.423305000
C	-1.230279000	2.591930000	-1.595719000
C	-0.019914000	3.506534000	-1.485488000
C	-0.537983000	4.679254000	-0.636735000
C	-1.958495000	4.891019000	-1.171901000
C	-1.338331000	1.249381000	-1.743324000
C	-2.713128000	0.673046000	-2.013309000
H	-3.388162000	0.835634000	-1.163767000
C	1.268957000	2.839869000	-1.077392000
C	1.531997000	1.550180000	-1.743969000
Co	0.208295000	0.262312000	-0.951982000
P	1.917991000	-0.673039000	0.318225000
C	2.120087000	3.401860000	-0.195351000
H	1.315202000	1.615811000	-2.820195000
H	2.570522000	1.221309000	-1.623483000
H	0.143919000	3.899051000	-2.512246000
H	1.918730000	4.360815000	0.286217000
H	3.055087000	2.906267000	0.075114000
H	0.095441000	5.574943000	-0.714349000
H	-0.566586000	4.376226000	0.424791000
H	-1.916127000	5.451957000	-2.119938000
H	-2.601570000	5.457916000	-0.482632000
H	-3.059691000	3.097212000	-0.561080000
H	-3.145196000	3.391966000	-2.298372000
H	-3.161177000	1.182663000	-2.891584000
P	-1.380884000	-0.947270000	0.627498000
C	-0.535246000	-1.273969000	2.256451000
H	-0.114751000	-0.313889000	2.583626000
H	-1.301096000	-1.543650000	2.997729000
C	1.444071000	-2.359917000	0.944963000
H	2.364599000	-2.928987000	1.139641000
H	0.934023000	-2.860117000	0.107117000
C	0.550919000	-2.358665000	2.198408000
H	1.183459000	-2.250438000	3.093077000
H	0.078647000	-3.351264000	2.267647000
C	-2.691613000	0.213786000	1.193362000
C	-4.066847000	-0.041870000	1.070868000
C	-2.272189000	1.453475000	1.710651000
C	-5.002250000	0.918652000	1.470294000
H	-4.415991000	-0.982833000	0.643178000
C	-3.208404000	2.409026000	2.111727000
H	-1.208639000	1.690171000	1.792342000
C	-4.577490000	2.144043000	1.992122000
H	-6.069005000	0.706564000	1.365855000
H	-2.865476000	3.367469000	2.508429000
H	-5.310236000	2.893675000	2.299890000
C	-2.185380000	-2.582834000	0.368406000
C	-3.018367000	-3.141855000	1.355796000
C	-1.889872000	-3.341480000	-0.772179000
C	-3.555838000	-4.420381000	1.188888000
H	-3.258452000	-2.580251000	2.261301000
C	-2.417829000	-4.625320000	-0.935878000
H	-1.239630000	-2.931030000	-1.539675000
C	-3.257240000	-5.165317000	0.042193000
H	-4.207492000	-4.837510000	1.960366000
H	-2.173206000	-5.201078000	-1.831647000
H	-3.675562000	-6.166687000	-0.085267000
C	3.501567000	-0.994781000	-0.556613000
C	4.547884000	-0.056469000	-0.510278000

C	3.664437000	-2.159488000	-1.327745000
C	5.730516000	-0.278786000	-1.220290000
H	4.450244000	0.847248000	0.094535000
C	4.850224000	-2.379705000	-2.034465000
H	2.871691000	-2.907804000	-1.382842000
C	5.884589000	-1.439990000	-1.984345000
H	6.535613000	0.458111000	-1.170974000
H	4.964588000	-3.291697000	-2.625061000
H	6.810816000	-1.613956000	-2.537130000
C	2.441672000	0.207777000	1.843618000
C	1.799006000	1.388839000	2.239950000
C	3.442968000	-0.339388000	2.666546000
C	2.143674000	2.016479000	3.440880000
H	1.039995000	1.833474000	1.597732000
C	3.785778000	0.287262000	3.866334000
H	3.959944000	-1.255577000	2.371220000
C	3.135560000	1.465041000	4.256170000
H	1.636007000	2.938013000	3.735018000
H	4.562837000	-0.145845000	4.500538000
H	3.405431000	1.952137000	5.196304000
O	-2.653626000	-0.713220000	-2.248757000
C	-1.719698000	-0.932288000	-3.280541000
H	-2.103586000	-0.526222000	-4.235910000
H	-1.602418000	-2.018368000	-3.395475000
C	-0.369711000	-0.275905000	-2.987990000
H	-0.083787000	0.507743000	-3.692648000
C	0.685710000	-1.019331000	-2.397037000
H	0.494150000	-2.058791000	-2.110427000
H	1.707293000	-0.841962000	-2.741890000

INT5, G = -3689.259907

C	3.258185000	-2.233314000	-1.122598000
C	1.979707000	-1.764642000	-1.768220000
C	1.074281000	-2.983296000	-1.943603000
C	2.038867000	-4.201160000	-1.865598000
C	3.464260000	-3.613642000	-1.766874000
C	1.740548000	-0.522986000	-2.239534000
C	2.761072000	0.604109000	-2.283702000
H	3.007362000	1.034620000	-1.302718000
C	-0.022590000	-2.901222000	-0.878862000
C	-1.193986000	-2.095802000	-1.223591000
Co	-0.234055000	-0.506962000	-0.504073000
P	-2.119326000	0.328970000	0.306458000
C	0.182465000	-3.346861000	0.391235000
H	-1.459856000	-2.052551000	-2.286572000
H	-2.076192000	-2.325686000	-0.611166000
H	0.586067000	-2.939521000	-2.927655000
H	1.110258000	-3.828362000	0.701828000
H	-0.613077000	-3.296688000	1.133487000
H	1.919903000	-4.851693000	-2.743765000
H	1.815165000	-4.820455000	-0.985773000
H	3.893664000	-3.485254000	-2.774078000
H	4.151335000	-4.253641000	-1.193435000
H	3.101825000	-2.343752000	-0.036751000
H	4.101039000	-1.540734000	-1.254426000
H	3.705002000	0.232885000	-2.729541000
P	1.076167000	0.824118000	1.026383000
C	0.048301000	1.062557000	2.556404000
H	-0.327275000	0.068366000	2.831757000
H	0.694709000	1.390287000	3.383116000

C	-1.827447000	2.025181000	0.995425000
H	-2.799003000	2.535106000	1.072603000
H	-1.237680000	2.564552000	0.237807000
C	-1.117447000	2.049845000	2.363096000
H	-1.856866000	1.857195000	3.155929000
H	-0.749680000	3.075774000	2.518841000
C	2.520771000	-0.127868000	1.648181000
C	3.842879000	0.253825000	1.369744000
C	2.289775000	-1.322677000	2.353283000
C	4.912213000	-0.547887000	1.783567000
H	4.049048000	1.182261000	0.833899000
C	3.358840000	-2.115445000	2.773934000
H	1.270772000	-1.655855000	2.559561000
C	4.674401000	-1.732438000	2.485353000
H	5.935419000	-0.240775000	1.554591000
H	3.163107000	-3.040540000	3.321268000
H	5.510529000	-2.357412000	2.807674000
C	1.709456000	2.532116000	0.762880000
C	2.324441000	3.214983000	1.831038000
C	1.544820000	3.192576000	-0.462952000
C	2.761477000	4.530341000	1.671275000
H	2.475976000	2.717500000	2.791782000
C	1.984472000	4.511963000	-0.620376000
H	1.112107000	2.679706000	-1.319285000
C	2.588981000	5.183612000	0.444144000
H	3.239458000	5.047392000	2.506740000
H	1.857438000	5.008554000	-1.585152000
H	2.931432000	6.213881000	0.320195000
C	-3.463751000	0.554431000	-0.920025000
C	-4.297653000	-0.531172000	-1.240758000
C	-3.641874000	1.776618000	-1.590003000
C	-5.289242000	-0.395632000	-2.214540000
H	-4.188362000	-1.484187000	-0.719824000
C	-4.637338000	1.908490000	-2.562747000
H	-3.005852000	2.635077000	-1.368550000
C	-5.460602000	0.823825000	-2.878949000
H	-5.933420000	-1.245759000	-2.450978000
H	-4.767256000	2.864900000	-3.074573000
H	-6.237895000	0.928974000	-3.639583000
C	-2.962079000	-0.536607000	1.693348000
C	-2.317871000	-1.553825000	2.409522000
C	-4.245987000	-0.123047000	2.093947000
C	-2.941379000	-2.159170000	3.504707000
H	-1.321283000	-1.875638000	2.111864000
C	-4.867134000	-0.725495000	3.189907000
H	-4.767467000	0.665994000	1.547186000
C	-4.217498000	-1.745422000	3.895908000
H	-2.426970000	-2.953742000	4.049978000
H	-5.864476000	-0.398087000	3.492772000
H	-4.708137000	-2.216583000	4.750917000
O	2.185149000	1.619999000	-3.092728000
C	1.221220000	1.008120000	-3.942013000
H	1.710769000	0.526519000	-4.812252000
H	0.535537000	1.787347000	-4.305040000
C	0.541932000	-0.052487000	-3.058619000
H	0.130395000	-0.876486000	-3.657746000
C	-0.532731000	0.519148000	-2.140052000
H	-0.371364000	1.588188000	-1.930722000
H	-1.531063000	0.392129000	-2.574713000

TS5, G = -3689.225649

C	3.429756000	-0.176953000	-2.073726000
C	1.985569000	0.219727000	-2.315060000
C	1.311299000	-0.874704000	-3.157359000
C	2.409472000	-1.944913000	-3.323391000
C	3.718781000	-1.157014000	-3.223072000
C	1.516639000	1.468892000	-2.080978000
C	2.321366000	2.585501000	-1.441005000
H	2.756519000	2.329301000	-0.465393000
C	-0.027460000	-1.349700000	-2.610979000
C	-1.077970000	-0.293228000	-2.469658000
Co	-0.128617000	0.164194000	-0.640865000
P	-2.003542000	-0.143380000	0.565016000
C	-0.325354000	-2.657172000	-2.461543000
H	-1.191595000	0.192789000	-3.445480000
H	-2.061646000	-0.690590000	-2.196707000
H	1.103233000	-0.428036000	-4.150103000
H	0.396995000	-3.449009000	-2.665642000
H	-1.326191000	-2.971109000	-2.161864000
H	2.299166000	-2.517140000	-4.255556000
H	2.365111000	-2.657179000	-2.484811000
H	3.909604000	-0.604264000	-4.158590000
H	4.592295000	-1.795092000	-3.023936000
H	3.541015000	-0.700334000	-1.117699000
H	4.106669000	0.689461000	-2.047980000
H	3.151058000	2.876837000	-2.119842000
P	1.074333000	-0.198127000	1.252326000
C	0.205284000	-1.175799000	2.578576000
H	-0.087187000	-2.121107000	2.101347000
H	0.945170000	-1.438281000	3.348111000
C	-1.820625000	0.461842000	2.310774000
H	-2.824793000	0.626258000	2.728726000
H	-1.331642000	1.444872000	2.241973000
C	-1.009491000	-0.478877000	3.220655000
H	-1.674923000	-1.261621000	3.616622000
H	-0.670632000	0.112510000	4.085049000
C	2.595720000	-1.209455000	1.059882000
C	3.881258000	-0.766330000	1.405121000
C	2.438619000	-2.483369000	0.483412000
C	4.991779000	-1.586996000	1.176955000
H	4.028802000	0.227200000	1.830682000
C	3.547820000	-3.301148000	0.262500000
H	1.449054000	-2.825784000	0.170234000
C	4.829005000	-2.852911000	0.607126000
H	5.989701000	-1.228337000	1.440228000
H	3.413518000	-4.285279000	-0.192451000
H	5.698882000	-3.488375000	0.425166000
C	1.550019000	1.314430000	2.188463000
C	2.182313000	1.229760000	3.443743000
C	1.188432000	2.575795000	1.692865000
C	2.459728000	2.387472000	4.173763000
H	2.465108000	0.259579000	3.858388000
C	1.470043000	3.734546000	2.423367000
H	0.692997000	2.667993000	0.726261000
C	2.105343000	3.643054000	3.664406000
H	2.952759000	2.309005000	5.145704000
H	1.191618000	4.708230000	2.013557000
H	2.323735000	4.547212000	4.237943000
C	-3.408564000	0.849821000	-0.076998000
C	-4.343568000	0.292451000	-0.966189000
C	-3.488294000	2.220672000	0.226352000

C	-5.340249000	1.089748000	-1.536293000
H	-4.307121000	-0.771786000	-1.209616000
C	-4.487022000	3.014322000	-0.343753000
H	-2.766833000	2.684948000	0.902039000
C	-5.414070000	2.451784000	-1.227538000
H	-6.062874000	0.641983000	-2.222677000
H	-4.538875000	4.077301000	-0.096516000
H	-6.193748000	3.073856000	-1.673539000
C	-2.704321000	-1.823512000	0.814349000
C	-1.939975000	-2.951070000	0.486623000
C	-3.962048000	-1.996379000	1.420433000
C	-2.420226000	-4.237177000	0.752662000
H	-0.966731000	-2.815740000	0.014759000
C	-4.441716000	-3.280894000	1.685186000
H	-4.571993000	-1.128284000	1.681972000
C	-3.671949000	-4.402859000	1.351978000
H	-1.814377000	-5.107536000	0.489770000
H	-5.420214000	-3.407497000	2.154604000
H	-4.050694000	-5.406305000	1.561016000
O	1.406447000	3.656875000	-1.277156000
C	0.529238000	3.612898000	-2.394497000
H	1.011107000	4.061276000	-3.285067000
H	-0.374268000	4.190365000	-2.152057000
C	0.249479000	2.113894000	-2.641814000
H	0.142895000	1.903555000	-3.717621000
C	-0.921621000	1.582494000	-1.852308000
H	-0.889871000	2.006979000	-0.821614000
H	-1.916238000	1.809664000	-2.247086000

INT6, G = -3689.289170

C	-2.396586000	0.462736000	-2.663042000
C	-1.229693000	-0.472661000	-2.388205000
C	-0.249748000	-0.373662000	-3.547591000
C	-0.724049000	0.845937000	-4.355762000
C	-2.247682000	0.792033000	-4.159809000
C	-1.175546000	-1.580410000	-1.551920000
C	-2.418859000	-2.157104000	-0.895680000
H	-2.837890000	-1.541487000	-0.092390000
C	1.106675000	-0.379461000	-2.879816000
C	1.832799000	-1.700533000	-2.863135000
Co	0.052935000	-0.002643000	-0.706242000
P	1.775896000	0.447895000	0.730554000
C	1.565768000	0.738154000	-2.249720000
H	1.785491000	-2.126153000	-3.879851000
H	2.893228000	-1.557002000	-2.608593000
H	-0.347773000	-1.270153000	-4.184681000
H	1.085026000	1.707046000	-2.403033000
H	2.579911000	0.765361000	-1.855223000
H	-0.407931000	0.804040000	-5.408155000
H	-0.330638000	1.781473000	-3.926414000
H	-2.675136000	-0.018854000	-4.773644000
H	-2.754780000	1.726901000	-4.441764000
H	-2.294672000	1.391032000	-2.085623000
H	-3.369277000	0.028873000	-2.385147000
H	-3.212371000	-2.260615000	-1.664271000
P	-1.368218000	0.539272000	0.960435000
C	-0.667865000	1.864370000	2.071921000
H	-0.282045000	2.645756000	1.402845000
H	-1.505972000	2.311050000	2.625826000
C	1.387855000	0.315390000	2.539592000

H	2.334751000	0.340396000	3.098566000
H	0.953503000	-0.681956000	2.690138000
C	0.426031000	1.402319000	3.052046000
H	1.005863000	2.291698000	3.343944000
H	-0.048235000	1.021532000	3.969335000
C	-2.948509000	1.344319000	0.461743000
C	-4.190305000	0.691582000	0.509496000
C	-2.889320000	2.656711000	-0.044431000
C	-5.345094000	1.331093000	0.046736000
H	-4.271193000	-0.316771000	0.918632000
C	-4.044016000	3.295144000	-0.500454000
H	-1.934891000	3.185207000	-0.103855000
C	-5.275296000	2.630939000	-0.462001000
H	-6.303575000	0.808358000	0.089280000
H	-3.980773000	4.313228000	-0.891787000
H	-6.178500000	3.128230000	-0.823450000
C	-1.836052000	-0.743463000	2.190753000
C	-2.725275000	-0.467062000	3.245186000
C	-1.210305000	-1.996071000	2.132505000
C	-2.984704000	-1.438040000	4.214770000
H	-3.221253000	0.504328000	3.310092000
C	-1.468510000	-2.966906000	3.103089000
H	-0.518310000	-2.204051000	1.317049000
C	-2.357571000	-2.688940000	4.144805000
H	-3.678239000	-1.217880000	5.029847000
H	-0.978530000	-3.941146000	3.038700000
H	-2.564722000	-3.446180000	4.904916000
C	3.161427000	-0.749453000	0.549216000
C	4.307446000	-0.457297000	-0.210118000
C	3.033517000	-2.030349000	1.118835000
C	5.300009000	-1.423870000	-0.397610000
H	4.443814000	0.533498000	-0.648023000
C	4.028985000	-2.992463000	0.933397000
H	2.148860000	-2.296900000	1.701163000
C	5.163279000	-2.693647000	0.171346000
H	6.185356000	-1.178799000	-0.989034000
H	3.913533000	-3.981274000	1.383378000
H	5.939890000	-3.447827000	0.023914000
C	2.553910000	2.115568000	0.656177000
C	1.911953000	3.152110000	-0.039731000
C	3.735507000	2.396607000	1.364736000
C	2.441778000	4.445794000	-0.035394000
H	0.988563000	2.948699000	-0.585415000
C	4.267225000	3.688320000	1.364269000
H	4.249690000	1.604520000	1.914416000
C	3.621653000	4.714707000	0.664512000
H	1.931042000	5.242725000	-0.580907000
H	5.188255000	3.894692000	1.914643000
H	4.039017000	5.724509000	0.666700000
O	-2.081980000	-3.434077000	-0.383561000
C	-0.784216000	-3.826953000	-0.805588000
H	-0.820676000	-4.855201000	-1.201634000
H	-0.094097000	-3.831276000	0.061334000
C	-0.309274000	-2.816555000	-1.859365000
H	-0.633040000	-3.167892000	-2.858929000
C	1.217077000	-2.687753000	-1.854103000
H	1.530382000	-2.386317000	-0.846619000
H	1.653274000	-3.686004000	-2.024004000

INT0′, G = -3689.204513

C	0.920878000	-3.067396000	1.544598000
C	0.848471000	-1.858318000	0.697868000
C	-1.210251000	-0.975140000	2.389852000
C	-0.364069000	-1.939919000	3.184620000
O	-0.105980000	-3.158096000	2.510967000
C	1.292805000	-1.129426000	-0.274362000
C	2.353354000	-1.008843000	-1.306182000
H	2.326580000	-0.016949000	-1.778583000
C	-1.025217000	0.394379000	2.400211000
H	-1.851758000	1.086743000	2.247310000
Co	-0.218289000	-0.316793000	0.563609000
P	-1.847203000	-0.941358000	-0.740773000
H	-0.158293000	0.821191000	2.918765000
H	-2.192210000	-1.374223000	2.114581000
H	-0.924637000	-2.216944000	4.093331000
H	0.575394000	-1.450255000	3.508908000
H	1.920476000	-3.086290000	2.029106000
H	0.841884000	-3.966894000	0.913124000
H	2.154557000	-1.744479000	-2.107000000
P	-0.117430000	1.757905000	-0.390548000
C	-0.899341000	1.795747000	-2.070920000
H	-1.986741000	1.878507000	-1.927991000
H	-0.589192000	2.708006000	-2.598616000
C	-1.394880000	-0.717740000	-2.533411000
H	-2.350561000	-0.718530000	-3.079725000
H	-0.850688000	-1.619913000	-2.843980000
C	-0.565847000	0.533877000	-2.883381000
H	-0.728890000	0.749876000	-3.950212000
H	0.504571000	0.306010000	-2.788721000
C	-0.826536000	3.214306000	0.490170000
C	-1.953690000	3.915822000	0.030544000
C	-0.237659000	3.601741000	1.708411000
C	-2.483622000	4.973707000	0.777995000
H	-2.438825000	3.648473000	-0.908408000
C	-0.765844000	4.659805000	2.449690000
H	0.646247000	3.080912000	2.083455000
C	-1.894679000	5.347114000	1.988550000
H	-3.361542000	5.506989000	0.405476000
H	-0.294168000	4.947682000	3.392276000
H	-2.310914000	6.172682000	2.570697000
C	1.638502000	2.265936000	-0.644026000
C	2.066419000	2.990578000	-1.768144000
C	2.584780000	1.902634000	0.330736000
C	3.414574000	3.330757000	-1.918671000
H	1.360150000	3.293607000	-2.542288000
C	3.928091000	2.256008000	0.185893000
H	2.283255000	1.307790000	1.195270000
C	4.348121000	2.964660000	-0.944101000
H	3.734650000	3.884044000	-2.804792000
H	4.651761000	1.955960000	0.947196000
H	5.401723000	3.225974000	-1.067055000
C	-2.154916000	-2.739973000	-0.540093000
C	-3.222858000	-3.201604000	0.248118000
C	-1.248193000	-3.670922000	-1.076241000
C	-3.383477000	-4.569270000	0.487048000
H	-3.938978000	-2.498423000	0.677415000
C	-1.417124000	-5.036679000	-0.842196000
H	-0.388318000	-3.339537000	-1.661725000
C	-2.484171000	-5.489572000	-0.058344000
H	-4.217666000	-4.914033000	1.102419000
H	-0.707303000	-5.749004000	-1.269093000

H	-2.611508000	-6.558346000	0.128843000
C	-3.507530000	-0.165142000	-0.619939000
C	-3.717561000	0.982374000	0.155263000
C	-4.577479000	-0.690390000	-1.370805000
C	-4.970527000	1.602822000	0.184239000
H	-2.899163000	1.412098000	0.726524000
C	-5.828911000	-0.073964000	-1.336195000
H	-4.439254000	-1.587734000	-1.978590000
C	-6.028334000	1.074307000	-0.558806000
H	-5.110772000	2.502486000	0.787654000
H	-6.652692000	-0.491416000	-1.919822000
H	-7.009089000	1.555230000	-0.536163000
C	3.750519000	-1.243700000	-0.707786000
C	4.867310000	-0.940530000	-1.705477000
H	4.732926000	0.086965000	-2.096494000
H	4.769186000	-1.597388000	-2.590268000
C	6.266616000	-1.058013000	-1.157353000
H	7.076359000	-0.937832000	-1.888578000
C	6.600750000	-1.270913000	0.111112000
C	6.190885000	-1.488301000	1.506391000
H	5.782340000	-0.642853000	2.073652000
H	5.769316000	-2.459828000	1.791833000
C	7.673044000	-1.434362000	1.102254000
H	8.241084000	-2.372683000	1.118382000
H	8.255755000	-0.555105000	1.403632000
H	3.868841000	-0.599848000	0.175423000
H	3.832812000	-2.281489000	-0.345312000

TS1′, G = −3689.180699

C	1.958324000	-2.459570000	2.062234000
C	1.002130000	-1.740436000	1.140256000
C	-0.400725000	-1.384328000	2.392818000
C	0.541412000	-1.547192000	3.578245000
O	1.392061000	-2.639594000	3.334809000
C	1.097736000	-1.393814000	-0.134049000
C	2.129406000	-1.624466000	-1.187985000
H	1.964862000	-0.941971000	-2.034844000
C	-1.086302000	-0.105772000	2.237183000
H	-2.172188000	-0.109203000	2.356071000
Co	-0.345027000	-0.370754000	0.420395000
P	-2.309942000	-0.439068000	-0.561697000
H	-0.602060000	0.742140000	2.743069000
H	-1.040667000	-2.270444000	2.280237000
H	-0.041662000	-1.763208000	4.485425000
H	1.109598000	-0.607735000	3.737376000
H	2.882070000	-1.848440000	2.121543000
H	2.229559000	-3.442939000	1.646541000
H	1.957451000	-2.637418000	-1.597303000
P	0.284970000	1.341422000	-0.730480000
C	0.102482000	1.144047000	-2.568120000
H	-0.062105000	2.144229000	-2.995707000
H	1.054760000	0.774492000	-2.969942000
C	-2.339536000	0.412381000	-2.211188000
H	-2.519306000	1.487787000	-2.063013000
H	-3.203335000	0.011226000	-2.760563000
C	-1.028614000	0.186950000	-2.977290000
H	-1.214202000	0.322106000	-4.053791000
H	-0.696637000	-0.857445000	-2.854170000
C	-0.562912000	2.929162000	-0.339164000
C	-1.554609000	2.989181000	0.648758000

C	-0.193402000	4.104460000	-1.020072000
C	-2.187737000	4.202805000	0.940027000
H	-1.837253000	2.087028000	1.188878000
C	-0.825516000	5.313230000	-0.725946000
H	0.597994000	4.085036000	-1.774069000
C	-1.827209000	5.363900000	0.252872000
H	-2.966911000	4.233175000	1.705215000
H	-0.533342000	6.220775000	-1.259618000
H	-2.321248000	6.311700000	0.479972000
C	2.048788000	1.735041000	-0.405509000
C	3.007717000	1.917557000	-1.413269000
C	2.442628000	1.821174000	0.942945000
C	4.344079000	2.160030000	-1.075753000
H	2.734469000	1.867032000	-2.468083000
C	3.774687000	2.070116000	1.274493000
H	1.706703000	1.683383000	1.739050000
C	4.730876000	2.231402000	0.263994000
H	5.085635000	2.283394000	-1.867906000
H	4.070013000	2.128447000	2.324650000
H	5.777664000	2.406389000	0.522485000
C	-2.710974000	-2.187707000	-1.001071000
C	-3.962620000	-2.465384000	-1.584359000
C	-1.808705000	-3.238937000	-0.784021000
C	-4.296092000	-3.770069000	-1.949478000
H	-4.688218000	-1.664062000	-1.744144000
C	-2.148668000	-4.546802000	-1.148445000
H	-0.835391000	-3.044413000	-0.334594000
C	-3.388866000	-4.814656000	-1.731929000
H	-5.269778000	-3.972789000	-2.401782000
H	-1.436914000	-5.356742000	-0.972315000
H	-3.652865000	-5.836300000	-2.015732000
C	-3.834996000	0.120398000	0.293483000
C	-4.418348000	-0.727826000	1.254335000
C	-4.397433000	1.388215000	0.073142000
C	-5.537843000	-0.313821000	1.978191000
H	-4.003180000	-1.722897000	1.432108000
C	-5.520299000	1.798639000	0.799064000
H	-3.968866000	2.074268000	-0.657513000
C	-6.090991000	0.952048000	1.753334000
H	-5.980774000	-0.983798000	2.718812000
H	-5.947502000	2.787042000	0.614250000
H	-6.967620000	1.275649000	2.319502000
C	3.577491000	-1.489366000	-0.692060000
C	4.591370000	-1.421491000	-1.831756000
H	4.316068000	-0.593351000	-2.512732000
H	4.526521000	-2.335358000	-2.453038000
C	6.019157000	-1.217064000	-1.395538000
H	6.761936000	-1.155467000	-2.201263000
C	6.444137000	-1.088580000	-0.142911000
H	3.664821000	-0.579393000	-0.081451000
H	3.828549000	-2.332132000	-0.029925000
C	6.140664000	-1.044498000	1.294218000
H	5.900501000	-1.980170000	1.813828000
H	5.619589000	-0.166723000	1.693904000
C	7.573288000	-0.859922000	0.768800000
H	8.292223000	-1.670070000	0.942169000
H	8.011015000	0.144765000	0.821045000

INT1′, G = -3689.195339

C	-2.614059000	3.228852000	0.068590000

C	-1.423416000	2.299876000	0.151788000
C	-0.580088000	2.767058000	1.304284000
C	-1.650663000	3.432767000	2.181507000
O	-2.553409000	4.032391000	1.251572000
C	-1.099048000	1.163941000	-0.499154000
C	-1.788234000	0.591913000	-1.714381000
H	-1.400155000	-0.412006000	-1.924629000
C	0.200727000	1.578946000	1.821440000
H	1.166869000	1.831802000	2.287348000
Co	0.170459000	0.169083000	0.485726000
P	1.819208000	0.928358000	-0.638817000
H	-0.408445000	0.994063000	2.548443000
H	0.104148000	3.554659000	0.942132000
H	-1.264701000	4.221604000	2.844042000
H	-2.168366000	2.671407000	2.800175000
H	-3.567356000	2.676649000	0.031408000
H	-2.569490000	3.890319000	-0.816194000
H	-1.530347000	1.203537000	-2.598979000
P	0.332713000	-2.043475000	-0.437646000
C	1.854681000	-2.221385000	-1.487259000
H	2.662851000	-2.168399000	-0.741848000
H	1.882550000	-3.233168000	-1.917405000
C	1.754444000	0.287298000	-2.373089000
H	2.387670000	0.935394000	-2.997215000
H	0.718289000	0.470650000	-2.689518000
C	2.133271000	-1.190554000	-2.601738000
H	3.213594000	-1.249111000	-2.804206000
H	1.641922000	-1.505367000	-3.534579000
C	0.710376000	-3.121502000	1.007967000
C	1.584531000	-4.220350000	0.922500000
C	0.106668000	-2.828804000	2.244390000
C	1.852678000	-4.998399000	2.051233000
H	2.063156000	-4.483443000	-0.022065000
C	0.371016000	-3.614282000	3.370873000
H	-0.582511000	-1.986734000	2.341676000
C	1.248392000	-4.697642000	3.277187000
H	2.536044000	-5.847156000	1.970916000
H	-0.107551000	-3.373154000	4.322886000
H	1.461466000	-5.308591000	4.157612000
C	-1.021063000	-2.923315000	-1.308731000
C	-1.088486000	-2.941432000	-2.712426000
C	-2.059131000	-3.511586000	-0.565608000
C	-2.177271000	-3.534369000	-3.358995000
H	-0.298365000	-2.489126000	-3.314165000
C	-3.143495000	-4.105303000	-1.215852000
H	-2.024256000	-3.510990000	0.526093000
C	-3.207759000	-4.115357000	-2.613506000
H	-2.216687000	-3.542434000	-4.450813000
H	-3.942433000	-4.560945000	-0.626064000
H	-4.058136000	-4.577572000	-3.120282000
C	1.951977000	2.740619000	-0.816684000
C	2.788222000	3.477020000	0.037758000
C	1.146669000	3.415612000	-1.750442000
C	2.815353000	4.872771000	-0.040124000
H	3.419858000	2.965880000	0.767342000
C	1.179970000	4.809185000	-1.825460000
H	0.477034000	2.864155000	-2.412586000
C	2.010822000	5.540483000	-0.968257000
H	3.467721000	5.438371000	0.629148000
H	0.550182000	5.326136000	-2.553144000
H	2.030601000	6.631349000	-1.025497000

C	3.411655000	0.375185000	0.067583000
C	3.453657000	-0.150164000	1.369616000
C	4.591136000	0.440314000	-0.694800000
C	4.659806000	-0.617607000	1.899613000
H	2.547934000	-0.193556000	1.979685000
C	5.793570000	-0.027296000	-0.161031000
H	4.576496000	0.859628000	-1.703621000
C	5.828068000	-0.559639000	1.133805000
H	4.684254000	-1.026915000	2.911936000
H	6.707298000	0.023981000	-0.757583000
H	6.770337000	-0.927398000	1.546881000
C	-3.325516000	0.504798000	-1.610286000
C	-3.821799000	-0.211740000	-0.338345000
H	-3.182741000	0.077906000	0.511224000
H	-3.699527000	-1.300045000	-0.471514000
C	-5.248893000	0.121848000	0.003787000
H	-6.033123000	-0.245089000	-0.671196000
C	-5.594762000	0.875572000	1.043457000
C	-5.152804000	1.639615000	2.220868000
H	-4.818964000	1.091147000	3.110613000
H	-4.644699000	2.601288000	2.075936000
C	-6.639975000	1.535741000	1.832981000
H	-7.292710000	0.924493000	2.468336000
H	-7.125406000	2.430970000	1.425347000
H	-3.704339000	-0.012654000	-2.507602000
H	-3.751261000	1.518720000	-1.649043000

INT2′, G = -3689.219397

C	-4.778430000	1.815600000	-1.017313000
C	-3.351478000	1.393650000	-1.299106000
C	-2.412463000	2.559692000	-0.987363000
C	-3.377737000	3.698128000	-0.555139000
C	-4.718313000	3.339943000	-1.206682000
C	-3.002486000	0.243806000	-1.880108000
C	-3.964920000	-0.888766000	-2.186417000
H	-4.517517000	-1.187896000	-1.276668000
C	-1.267803000	2.243728000	-0.024896000
C	-0.165074000	3.294440000	0.140449000
Co	0.070937000	0.917804000	-0.084887000
P	2.366923000	0.283248000	0.375156000
C	-1.097931000	2.964624000	1.284354000
H	-0.274638000	4.220808000	-0.432073000
H	0.894463000	3.028826000	0.288792000
H	-1.934659000	2.858182000	-1.937821000
H	-1.842635000	3.702584000	1.592778000
H	-0.643188000	2.448606000	2.138509000
H	-3.003493000	4.698606000	-0.820100000
H	-3.516895000	3.677353000	0.536640000
H	-4.702523000	3.582799000	-2.283192000
H	-5.570008000	3.872237000	-0.755841000
H	-5.052314000	1.581063000	0.027227000
H	-5.515574000	1.317298000	-1.664745000
H	-4.710590000	-0.619951000	-2.957154000
P	-0.655749000	-0.750528000	1.104650000
C	-0.060761000	-0.351563000	2.837606000
H	-0.781228000	0.367703000	3.253258000
H	-0.187382000	-1.278935000	3.414974000
C	2.448785000	-0.432767000	2.084071000
H	3.454930000	-0.282671000	2.500189000
H	2.302304000	-1.518707000	1.993872000

C	1.375611000	0.195257000	2.995145000
H	1.371144000	1.288322000	2.846044000
H	1.670058000	0.044721000	4.045067000
C	-2.451701000	-1.034310000	1.347849000
C	-3.069853000	-2.261268000	1.057904000
C	-3.230894000	0.025813000	1.844343000
C	-4.441389000	-2.427447000	1.276092000
H	-2.492767000	-3.096185000	0.659103000
C	-4.595905000	-0.148699000	2.072433000
H	-2.777574000	0.998799000	2.037985000
C	-5.205866000	-1.376511000	1.788013000
H	-4.910213000	-3.385679000	1.041032000
H	-5.187902000	0.680861000	2.465646000
H	-6.276437000	-1.509585000	1.960369000
C	0.064783000	-2.417897000	0.795369000
C	-0.058740000	-3.430728000	1.766204000
C	0.779422000	-2.690311000	-0.379850000
C	0.531830000	-4.678977000	1.563047000
H	-0.626197000	-3.257895000	2.683006000
C	1.376524000	-3.937794000	-0.579615000
H	0.885353000	-1.926367000	-1.144813000
C	1.256149000	-4.933529000	0.391876000
H	0.426609000	-5.455715000	2.323938000
H	1.944350000	-4.120013000	-1.494321000
H	1.724254000	-5.908889000	0.239528000
C	3.392309000	-0.797747000	-0.691321000
C	3.281629000	-0.666077000	-2.086971000
C	4.258783000	-1.767617000	-0.160758000
C	4.021717000	-1.492679000	-2.936007000
H	2.610548000	0.077506000	-2.521942000
C	4.992915000	-2.596402000	-1.013751000
H	4.369752000	-1.892689000	0.917241000
C	4.874860000	-2.463696000	-2.400812000
H	3.925124000	-1.381578000	-4.018498000
H	5.659896000	-3.350392000	-0.589067000
H	5.447242000	-3.116047000	-3.064517000
C	3.316177000	1.863268000	0.458635000
C	3.916294000	2.352642000	1.629021000
C	3.377416000	2.637239000	-0.716067000
C	4.573981000	3.588544000	1.620041000
H	3.879974000	1.787545000	2.561185000
C	4.045026000	3.862453000	-0.724556000
H	2.891161000	2.287586000	-1.630520000
C	4.644029000	4.342916000	0.446415000
H	5.034059000	3.958913000	2.539300000
H	4.089765000	4.448295000	-1.645652000
H	5.159201000	5.306362000	0.443255000
O	-3.175389000	-1.969922000	-2.679569000
C	-1.821501000	-1.729231000	-2.334866000
H	-1.172581000	-2.274307000	-3.037848000
H	-1.605607000	-2.099369000	-1.318143000
C	-1.654299000	-0.205027000	-2.405249000
H	-1.669810000	0.040154000	-3.489804000
C	-0.347357000	0.362825000	-1.876287000
H	0.471320000	-0.310687000	-2.181476000
H	-0.139626000	1.353117000	-2.327737000

TS3′, G = –3689.155007

C	-4.639216000	2.337482000	-0.589093000
C	-3.339340000	1.767354000	-1.116212000

C	-2.275349000	2.869906000	-1.105102000
C	-3.044594000	4.140388000	-0.635580000
C	-4.521282000	3.834561000	-0.920989000
C	-3.184104000	0.544696000	-1.635010000
C	-4.280046000	-0.501059000	-1.714188000
H	-4.661597000	-0.752746000	-0.709414000
C	-1.077999000	2.582180000	-0.246327000
C	0.331563000	2.876168000	-0.357172000
Co	0.123969000	0.992809000	-0.172240000
P	2.304227000	0.346867000	0.431714000
C	-0.829160000	2.546471000	1.168586000
H	0.965998000	2.248311000	-1.078304000
H	0.885111000	3.785595000	-0.103900000
H	-1.904569000	3.016887000	-2.133473000
H	-1.261887000	1.806689000	1.847077000
H	-0.392762000	3.403653000	1.695431000
H	-2.678617000	5.055940000	-1.123623000
H	-2.901751000	4.267664000	0.450631000
H	-4.743243000	3.994509000	-1.989948000
H	-5.209307000	4.467287000	-0.339464000
H	-4.700740000	2.202282000	0.507439000
H	-5.532286000	1.859054000	-1.018925000
H	-5.141373000	-0.160029000	-2.317367000
P	-0.636782000	-0.776223000	1.002234000
C	-0.147238000	-0.436880000	2.781161000
H	-0.949248000	0.184355000	3.204328000
H	-0.211350000	-1.395687000	3.312988000
C	2.373461000	-0.309316000	2.175513000
H	3.345630000	-0.028881000	2.605406000
H	2.333439000	-1.407926000	2.128478000
C	1.214658000	0.229485000	3.030114000
H	1.116830000	1.316479000	2.892909000
H	1.465379000	0.085846000	4.092928000
C	-2.427280000	-1.170885000	1.249619000
C	-2.941069000	-2.461131000	1.039504000
C	-3.294929000	-0.167223000	1.716454000
C	-4.283866000	-2.744501000	1.309530000
H	-2.298963000	-3.259851000	0.667141000
C	-4.632033000	-0.453478000	1.994427000
H	-2.943286000	0.854635000	1.859214000
C	-5.131105000	-1.746064000	1.795000000
H	-4.664076000	-3.754027000	1.137770000
H	-5.288566000	0.339878000	2.358730000
H	-6.178972000	-1.968922000	2.008775000
C	0.131050000	-2.422817000	0.657218000
C	0.222612000	-3.408809000	1.659743000
C	0.625504000	-2.724678000	-0.619949000
C	0.804747000	-4.648260000	1.388152000
H	-0.171018000	-3.231123000	2.661120000
C	1.206541000	-3.965574000	-0.893666000
H	0.575982000	-1.982874000	-1.408865000
C	1.301784000	-4.930107000	0.110430000
H	0.866788000	-5.398076000	2.180254000
H	1.599592000	-4.163395000	-1.892714000
H	1.762067000	-5.898843000	-0.097812000
C	3.183030000	-0.861467000	-0.622580000
C	3.012055000	-0.765716000	-2.015086000
C	4.006004000	-1.871017000	-0.099874000
C	3.639278000	-1.675181000	-2.868983000
H	2.381159000	0.020081000	-2.436889000
C	4.626375000	-2.784370000	-0.956735000

H	4.167113000	-1.962269000	0.975360000
C	4.441526000	-2.692245000	-2.339537000
H	3.495000000	-1.593589000	-3.948762000
H	5.255774000	-3.573591000	-0.538983000
H	4.923908000	-3.411664000	-3.005462000
C	3.438996000	1.802261000	0.473554000
C	3.152787000	2.865257000	1.351765000
C	4.565871000	1.895963000	-0.359824000
C	3.981052000	3.988873000	1.401321000
H	2.278867000	2.826476000	2.003672000
C	5.386636000	3.028102000	-0.314722000
H	4.814109000	1.084692000	-1.045535000
C	5.098961000	4.075694000	0.564352000
H	3.747139000	4.801514000	2.093245000
H	6.259446000	3.085193000	-0.969731000
H	5.743108000	4.957585000	0.598432000
O	-3.712684000	-1.645957000	-2.344922000
C	-2.300592000	-1.551811000	-2.279587000
H	-1.869307000	-2.112556000	-3.123652000
H	-1.915446000	-2.003429000	-1.349487000
C	-1.976187000	-0.052602000	-2.333747000
H	-2.089526000	0.240846000	-3.399199000
C	-0.552771000	0.328691000	-1.937742000
H	0.130166000	-0.470981000	-2.260517000
H	-0.260093000	1.217136000	-2.517078000

TS5′, G = −3689.238418

C	-4.068772000	-0.384946000	2.072105000
C	-2.582766000	-0.685253000	2.221085000
C	-2.316796000	-2.093065000	1.667481000
C	-3.597148000	-2.407144000	0.867704000
C	-4.707322000	-1.723940000	1.668550000
C	-1.741630000	0.178305000	2.810378000
C	-2.182534000	1.548385000	3.296702000
H	-2.848565000	2.074771000	2.595769000
C	-1.006026000	-2.335711000	0.929781000
C	0.226116000	-2.258952000	1.654928000
Co	0.093682000	-0.776662000	0.010390000
P	2.138887000	-0.354586000	-0.785229000
C	-0.885302000	-2.538212000	-0.465009000
H	0.196300000	-2.292682000	2.741023000
H	1.112507000	-2.747876000	1.241117000
H	-2.295983000	-2.774928000	2.541434000
H	-1.781119000	-2.575334000	-1.082146000
H	-0.048085000	-3.136653000	-0.844208000
H	-3.744886000	-3.487349000	0.720669000
H	-3.541716000	-1.939358000	-0.125897000
H	-4.946530000	-2.315246000	2.568710000
H	-5.636394000	-1.599483000	1.092654000
H	-4.216568000	0.364073000	1.275654000
H	-4.501911000	0.041243000	2.990796000
H	-2.713264000	1.470449000	4.270582000
P	-0.868121000	0.308383000	-1.647002000
C	-0.156437000	0.040182000	-3.348387000
H	-0.957596000	-0.380822000	-3.969250000
H	0.082215000	1.018858000	-3.787341000
C	2.286378000	-0.309898000	-2.644388000
H	3.208485000	-0.847307000	-2.912575000
H	2.443094000	0.745947000	-2.915520000
C	1.074956000	-0.870727000	-3.396920000

H	0.808671000	-1.873903000	-3.025923000
H	1.354884000	-1.000705000	-4.454940000
C	-2.681216000	0.061437000	-1.900446000
C	-3.630312000	0.908396000	-1.306024000
C	-3.135338000	-1.042668000	-2.647209000
C	-4.997958000	0.648814000	-1.441836000
H	-3.313427000	1.779863000	-0.732823000
C	-4.501409000	-1.298891000	-2.782298000
H	-2.427609000	-1.722047000	-3.127259000
C	-5.438233000	-0.456933000	-2.173434000
H	-5.720720000	1.317655000	-0.968784000
H	-4.833059000	-2.163170000	-3.362324000
H	-6.506875000	-0.660125000	-2.273919000
C	-0.699223000	2.096680000	-1.235056000
C	-0.237853000	3.081756000	-2.121114000
C	-0.986379000	2.462371000	0.093768000
C	-0.054919000	4.396819000	-1.679413000
H	-0.004401000	2.843588000	-3.159374000
C	-0.804275000	3.773461000	0.535200000
H	-1.334532000	1.706895000	0.797440000
C	-0.332112000	4.745365000	-0.353842000
H	0.314548000	5.150680000	-2.378533000
H	-1.003445000	4.008196000	1.582447000
H	-0.174357000	5.771396000	-0.013117000
C	3.210399000	1.047859000	-0.273836000
C	2.620393000	2.244190000	0.163761000
C	4.611359000	0.976271000	-0.387899000
C	3.414292000	3.348011000	0.490688000
H	1.537979000	2.329699000	0.241953000
C	5.401753000	2.080180000	-0.060244000
H	5.090137000	0.055628000	-0.729008000
C	4.805160000	3.267467000	0.381836000
H	2.937594000	4.271061000	0.828889000
H	6.488625000	2.013414000	-0.151299000
H	5.426503000	4.128843000	0.639075000
C	3.069354000	-1.859517000	-0.284475000
C	2.967736000	-3.036920000	-1.046617000
C	3.757318000	-1.900799000	0.941719000
C	3.543738000	-4.227365000	-0.592885000
H	2.436188000	-3.041493000	-2.000102000
C	4.332385000	-3.091600000	1.391674000
H	3.854833000	-0.998742000	1.549050000
C	4.224426000	-4.259310000	0.628157000
H	3.457818000	-5.132894000	-1.198082000
H	4.868549000	-3.105583000	2.343598000
H	4.672147000	-5.190569000	0.982926000
O	-0.984525000	2.295394000	3.431431000
C	-0.002849000	1.389442000	3.906743000
H	-0.103170000	1.249195000	5.001324000
H	0.991492000	1.810524000	3.697898000
C	-0.266396000	0.041695000	3.175333000
H	-0.143341000	-0.780542000	3.898691000
C	0.695975000	-0.141930000	2.009494000
H	0.711537000	0.790137000	1.425657000
H	1.717517000	-0.355353000	2.342037000

INT4 (R = Me), G = -3728.482035

C	2.553885000	-3.559169000	-1.310530000
C	1.293344000	-2.701273000	-1.397707000
C	0.104520000	-3.613456000	-1.151663000

C	0.685645000	-4.695134000	-0.228531000
C	2.074585000	-4.950379000	-0.835830000
C	1.325367000	-1.362030000	-1.570363000
C	2.637703000	-0.714565000	-1.939349000
H	3.364424000	-0.804187000	-1.119500000
C	-1.171615000	-2.910822000	-0.761059000
C	-1.470947000	-1.706394000	-1.561225000
Co	-0.190195000	-0.345577000	-0.880792000
P	-1.945696000	0.646926000	0.424163000
C	-1.986912000	-3.383329000	0.204701000
H	-1.237320000	-1.883847000	-2.614441000
H	-2.513858000	-1.376657000	-1.475644000
H	-0.104193000	-4.104201000	-2.127362000
H	-1.757667000	-4.287352000	0.772979000
H	-2.919049000	-2.872699000	0.454482000
H	0.063100000	-5.600929000	-0.178181000
H	0.775568000	-4.291773000	0.795474000
H	1.977331000	-5.632234000	-1.696362000
H	2.773653000	-5.417934000	-0.126540000
H	3.259907000	-3.112655000	-0.591660000
H	3.083846000	-3.596695000	-2.278504000
H	3.080290000	-1.219120000	-2.822615000
P	1.404526000	0.994334000	0.675933000
C	0.563519000	1.250072000	2.320899000
H	0.182851000	0.262843000	2.612751000
H	1.319668000	1.514177000	3.073732000
C	-1.458029000	2.325599000	1.061118000
H	-2.373958000	2.896799000	1.272438000
H	-0.945101000	2.838578000	0.232217000
C	-0.562247000	2.295821000	2.312045000
H	-1.189627000	2.125165000	3.200944000
H	-0.124674000	3.300258000	2.425475000
C	2.785939000	-0.094325000	1.215667000
C	4.138789000	0.278616000	1.180125000
C	2.445942000	-1.393239000	1.635750000
C	5.130327000	-0.628032000	1.570994000
H	4.427594000	1.271635000	0.832427000
C	3.436840000	-2.293392000	2.032228000
H	1.403822000	-1.720197000	1.624630000
C	4.783460000	-1.912624000	2.000195000
H	6.180062000	-0.326666000	1.534802000
H	3.156432000	-3.300277000	2.350682000
H	5.560313000	-2.619076000	2.302187000
C	2.136583000	2.670003000	0.425265000
C	2.809982000	3.315424000	1.480937000
C	1.973921000	3.359384000	-0.784988000
C	3.308868000	4.610210000	1.322937000
H	2.958679000	2.806845000	2.435693000
C	2.464705000	4.659295000	-0.941949000
H	1.465873000	2.882525000	-1.617583000
C	3.134643000	5.287641000	0.110775000
H	3.833242000	5.092085000	2.151646000
H	2.323511000	5.178740000	-1.892749000
H	3.520372000	6.302760000	-0.010791000
C	-3.512277000	0.973561000	-0.478161000
C	-4.520805000	-0.006447000	-0.526141000
C	-3.697195000	2.172774000	-1.189649000
C	-5.684528000	0.208401000	-1.268452000
H	-4.411852000	-0.936847000	0.034770000
C	-4.863703000	2.384569000	-1.930974000
H	-2.935755000	2.954647000	-1.173261000

C	-5.858610000	1.403384000	-1.974466000
H	-6.459725000	-0.561158000	-1.290170000
H	-4.993664000	3.323519000	-2.474293000
H	-6.769796000	1.570991000	-2.553555000
C	-2.504187000	-0.203330000	1.956565000
C	-1.771959000	-1.279891000	2.478089000
C	-3.619127000	0.275314000	2.669019000
C	-2.142005000	-1.871784000	3.689271000
H	-0.918783000	-1.673076000	1.926748000
C	-3.990223000	-0.318222000	3.877497000
H	-4.203189000	1.112465000	2.279599000
C	-3.251885000	-1.392187000	4.389939000
H	-1.563173000	-2.711851000	4.079997000
H	-4.858401000	0.060329000	4.422159000
H	-3.544420000	-1.854752000	5.335708000
O	2.508624000	0.669977000	-2.209505000
C	1.665550000	0.866493000	-3.312384000
H	2.082009000	0.354770000	-4.199818000
H	1.652784000	1.946226000	-3.530087000
C	0.215072000	0.406025000	-3.123858000
C	-0.667771000	1.097051000	-2.297202000
H	-0.354525000	2.044010000	-1.851244000
H	-1.735271000	1.012361000	-2.513708000
C	-0.291140000	-0.456143000	-4.244778000
H	-1.364696000	-0.665555000	-4.178712000
H	-0.119933000	0.105851000	-5.180898000
H	0.272986000	-1.396799000	-4.326428000

TS4 (R = Me), G = -3728.477672

C	2.781256000	-3.450508000	-1.125939000
C	1.467099000	-2.684641000	-1.275320000
C	0.331306000	-3.672377000	-1.052001000
C	0.987083000	-4.787843000	-0.220027000
C	2.382203000	-4.910010000	-0.841384000
C	1.445245000	-1.346107000	-1.485134000
C	2.765827000	-0.664204000	-1.767626000
H	3.433445000	-0.701498000	-0.897723000
C	-0.960015000	-3.072966000	-0.550175000
C	-1.411606000	-1.874086000	-1.282557000
Co	-0.172063000	-0.406783000	-0.740476000
P	-1.973187000	0.601659000	0.370485000
C	-1.661867000	-3.626213000	0.460893000
H	-1.294022000	-2.019432000	-2.360396000
H	-2.464528000	-1.643469000	-1.086230000
H	0.105843000	-4.102856000	-2.051552000
H	-1.331636000	-4.527495000	0.981104000
H	-2.606822000	-3.188689000	0.790367000
H	0.414236000	-5.726731000	-0.237106000
H	1.066705000	-4.460336000	0.831499000
H	2.319325000	-5.476870000	-1.785185000
H	3.103143000	-5.429418000	-0.192779000
H	3.354129000	-3.027536000	-0.282681000
H	3.421571000	-3.339666000	-2.017312000
H	3.288709000	-1.175529000	-2.601871000
P	1.302048000	1.102204000	0.671492000
C	0.429513000	1.444147000	2.284750000
H	0.080051000	0.468370000	2.647763000
H	1.166551000	1.796523000	3.020507000
C	-1.591057000	2.342798000	0.903691000
H	-2.542002000	2.876163000	1.047540000

H	-1.080148000	2.824364000	0.055227000
C	-0.732634000	2.442749000	2.176292000
H	-1.377941000	2.312519000	3.059091000
H	-0.335530000	3.468832000	2.225967000
C	2.723067000	0.124867000	1.311289000
C	4.060299000	0.549375000	1.273745000
C	2.427745000	-1.157359000	1.809215000
C	5.079186000	-0.288971000	1.739668000
H	4.318075000	1.525341000	0.860269000
C	3.445496000	-1.990190000	2.278340000
H	1.399496000	-1.526232000	1.803926000
C	4.776173000	-1.556747000	2.245366000
H	6.116646000	0.051802000	1.700302000
H	3.198461000	-2.984738000	2.657330000
H	5.574766000	-2.209828000	2.605147000
C	1.932937000	2.790319000	0.294070000
C	2.637776000	3.528661000	1.263549000
C	1.632261000	3.404456000	-0.930042000
C	3.045634000	4.838794000	1.001258000
H	2.878137000	3.084615000	2.231836000
C	2.030934000	4.718688000	-1.190660000
H	1.075854000	2.858706000	-1.687937000
C	2.743552000	5.437467000	-0.227047000
H	3.598228000	5.395000000	1.762321000
H	1.783595000	5.178341000	-2.150497000
H	3.059916000	6.463619000	-0.429300000
C	-3.491632000	0.815402000	-0.642641000
C	-4.514992000	-0.149511000	-0.622508000
C	-3.615933000	1.918906000	-1.506164000
C	-5.633996000	-0.013386000	-1.448403000
H	-4.452503000	-1.005746000	0.052203000
C	-4.736989000	2.052051000	-2.330230000
H	-2.840211000	2.685426000	-1.547391000
C	-5.747251000	1.085660000	-2.305745000
H	-6.421886000	-0.769685000	-1.417455000
H	-4.819287000	2.916742000	-2.992998000
H	-6.623087000	1.191124000	-2.950381000
C	-2.583970000	-0.157398000	1.927123000
C	-1.857129000	-1.185608000	2.543523000
C	-3.733676000	0.347442000	2.562131000
C	-2.269353000	-1.707435000	3.773651000
H	-0.974518000	-1.595809000	2.052957000
C	-4.144891000	-0.174531000	3.790033000
H	-4.313643000	1.147578000	2.095950000
C	-3.412969000	-1.202492000	4.397911000
H	-1.696147000	-2.512447000	4.239267000
H	-5.039508000	0.223024000	4.275159000
H	-3.737292000	-1.609434000	5.358774000
O	2.580321000	0.696706000	-2.099525000
C	1.737637000	0.744893000	-3.218905000
H	2.249257000	0.300852000	-4.093413000
H	1.538373000	1.801507000	-3.448935000
C	0.398714000	0.011776000	-3.019094000
C	-0.664203000	0.683340000	-2.363608000
H	-0.527788000	1.740949000	-2.116777000
H	-1.680735000	0.435650000	-2.682204000
C	0.102490000	-0.982731000	-4.112717000
H	-0.876514000	-1.463868000	-4.014694000
H	0.081672000	-0.408400000	-5.056795000
H	0.884170000	-1.748645000	-4.204946000

INT5 (R = Me), G = -3728.522827

C	3.370014000	-1.724985000	1.659769000
C	2.005191000	-2.159788000	1.167448000
C	1.135128000	-2.444890000	2.398811000
C	2.133236000	-2.502015000	3.590808000
C	3.532811000	-2.542792000	2.950398000
C	1.690543000	-2.367539000	-0.132730000
C	2.681375000	-2.123102000	-1.262933000
H	3.046884000	-1.089525000	-1.320724000
C	0.092970000	-1.331140000	2.493010000
C	-1.219666000	-1.568897000	2.020848000
Co	-0.156181000	-0.525550000	0.487025000
P	-2.078261000	0.492975000	-0.088909000
C	0.532615000	-0.012925000	2.649306000
H	-1.557150000	-2.587665000	1.830461000
H	-2.011105000	-0.883386000	2.334659000
H	0.618197000	-3.403817000	2.289642000
H	1.580993000	0.192605000	2.854916000
H	-0.156572000	0.779575000	2.931800000
H	1.936090000	-3.367978000	4.238564000
H	2.030528000	-1.602691000	4.215955000
H	3.805453000	-3.580268000	2.695198000
H	4.317190000	-2.145790000	3.611712000
H	3.372234000	-0.647085000	1.892665000
H	4.172735000	-1.888261000	0.927157000
H	3.562675000	-2.783125000	-1.127298000
P	1.109684000	1.264783000	-0.351449000
C	0.103900000	2.797697000	-0.025800000
H	-0.267727000	2.706133000	1.004547000
H	0.765625000	3.675268000	-0.046445000
C	-1.785727000	1.702003000	-1.459499000
H	-2.756558000	1.954776000	-1.910152000
H	-1.200888000	1.169479000	-2.223354000
C	-1.063025000	2.984947000	-1.012858000
H	-1.790841000	3.669461000	-0.550338000
H	-0.691981000	3.484592000	-1.920513000
C	2.705894000	1.638541000	0.488681000
C	3.930795000	1.284844000	-0.101641000
C	2.715630000	2.252996000	1.753569000
C	5.135367000	1.509504000	0.571079000
H	3.956516000	0.844525000	-1.099772000
C	3.920886000	2.480916000	2.422044000
H	1.782749000	2.557022000	2.231182000
C	5.134046000	2.100682000	1.837490000
H	6.077744000	1.222891000	0.098490000
H	3.910651000	2.959828000	3.403992000
H	6.075253000	2.274753000	2.364172000
C	1.550858000	1.493820000	-2.126817000
C	2.111070000	2.716692000	-2.546083000
C	1.307035000	0.494347000	-3.076379000
C	2.409133000	2.930960000	-3.892076000
H	2.327442000	3.504472000	-1.820573000
C	1.613808000	0.708531000	-4.425346000
H	0.909903000	-0.470400000	-2.772254000
C	2.158855000	1.926340000	-4.836429000
H	2.841507000	3.884145000	-4.205790000
H	1.428783000	-0.087196000	-5.150725000
H	2.395652000	2.095112000	-5.889812000
C	-3.372925000	-0.630617000	-0.746826000
C	-4.187270000	-1.336678000	0.155756000

C	-3.526683000	-0.850279000	-2.125179000
C	-5.135418000	-2.247841000	-0.312884000
H	-4.093908000	-1.169706000	1.230606000
C	-4.480431000	-1.761008000	-2.590725000
H	-2.902255000	-0.326439000	-2.850299000
C	-5.283488000	-2.463384000	-1.687915000
H	-5.763983000	-2.788060000	0.398917000
H	-4.592107000	-1.921456000	-3.665627000
H	-6.026541000	-3.175796000	-2.054128000
C	-3.065933000	1.525853000	1.087697000
C	-2.608079000	1.916546000	2.351964000
C	-4.319099000	1.998203000	0.648018000
C	-3.376262000	2.756854000	3.165538000
H	-1.646715000	1.570539000	2.719459000
C	-5.084042000	2.839136000	1.457136000
H	-4.708417000	1.703863000	-0.329032000
C	-4.615251000	3.220714000	2.720008000
H	-2.999271000	3.044861000	4.149459000
H	-6.053101000	3.195510000	1.099910000
H	-5.217145000	3.876353000	3.353668000
O	2.002436000	-2.430320000	-2.465952000
C	1.028645000	-3.404143000	-2.148530000
H	1.493964000	-4.408302000	-2.071259000
H	0.272842000	-3.427345000	-2.947219000
C	0.439137000	-2.992749000	-0.775835000
C	-0.617732000	-1.884265000	-0.922776000
H	-0.589374000	-1.423040000	-1.921125000
H	-1.617332000	-2.302905000	-0.768743000
C	-0.115835000	-4.246386000	-0.090028000
H	0.688957000	-4.949470000	0.174842000
H	-0.687249000	-4.021974000	0.816717000
H	-0.806741000	-4.756239000	-0.780746000

TS5 (R = Me), G = -3728.486230

C	-3.388912000	-0.797924000	-1.864761000
C	-1.913084000	-1.136024000	-1.995144000
C	-1.339109000	-0.350860000	-3.185425000
C	-2.527504000	0.498066000	-3.683981000
C	-3.762032000	-0.307890000	-3.273461000
C	-1.352657000	-2.177338000	-1.330224000
C	-2.074923000	-2.969954000	-0.256606000
H	-2.582619000	-2.351632000	0.495851000
C	-0.052386000	0.402109000	-2.877541000
C	1.079190000	-0.417433000	-2.336277000
Co	0.142879000	-0.334428000	-0.459194000
P	1.981028000	0.489042000	0.559449000
C	0.134633000	1.682385000	-3.258727000
H	1.319575000	-1.183212000	-3.079002000
H	1.999038000	0.161164000	-2.194164000
H	-1.094503000	-1.086248000	-3.973276000
H	-0.644908000	2.263008000	-3.754318000
H	1.098332000	2.174812000	-3.120726000
H	-2.468089000	0.707544000	-4.761555000
H	-2.544422000	1.462537000	-3.155087000
H	-3.904375000	-1.167292000	-3.950452000
H	-4.687867000	0.285915000	-3.282393000
H	-3.546469000	0.012808000	-1.144706000
H	-3.989139000	-1.653920000	-1.524246000
H	-2.831170000	-3.634584000	-0.725883000
P	-1.128388000	0.699286000	1.131006000

C	-0.304186000	2.161558000	1.942197000
H	-0.011787000	2.826737000	1.118775000
H	-1.065004000	2.704000000	2.521383000
C	1.770597000	0.630842000	2.395047000
H	2.767391000	0.688855000	2.856627000
H	1.316009000	-0.317606000	2.717378000
C	0.903776000	1.823947000	2.836348000
H	1.534289000	2.724357000	2.900709000
H	0.550332000	1.614483000	3.857636000
C	-2.676537000	1.499848000	0.544805000
C	-3.954564000	1.188077000	1.034431000
C	-2.549698000	2.444085000	-0.490056000
C	-5.085372000	1.813411000	0.497473000
H	-4.079749000	0.440830000	1.819089000
C	-3.679460000	3.070333000	-1.018997000
H	-1.567873000	2.664501000	-0.916010000
C	-4.951729000	2.754456000	-0.527597000
H	-6.075932000	1.555677000	0.879763000
H	-3.566917000	3.796109000	-1.827859000
H	-5.837069000	3.237097000	-0.948160000
C	-1.577916000	-0.296487000	2.614505000
C	-2.241195000	0.291353000	3.709045000
C	-1.159747000	-1.632079000	2.709929000
C	-2.493926000	-0.451151000	4.864195000
H	-2.566479000	1.333162000	3.665654000
C	-1.415870000	-2.374802000	3.867654000
H	-0.636621000	-2.110614000	1.881513000
C	-2.082561000	-1.787395000	4.945617000
H	-3.011231000	0.016230000	5.705553000
H	-1.090657000	-3.416581000	3.919776000
H	-2.280474000	-2.366273000	5.851119000
C	3.414435000	-0.645388000	0.383190000
C	4.341329000	-0.481302000	-0.660245000
C	3.515967000	-1.773986000	1.215715000
C	5.353535000	-1.424191000	-0.862142000
H	4.284679000	0.390540000	-1.315577000
C	4.530457000	-2.713256000	1.012874000
H	2.799435000	-1.937654000	2.023390000
C	5.450286000	-2.541979000	-0.027172000
H	6.069692000	-1.281800000	-1.674949000
H	4.600178000	-3.583385000	1.669968000
H	6.241860000	-3.278204000	-0.185921000
C	2.648619000	2.139010000	0.107167000
C	1.861283000	3.016439000	-0.649598000
C	3.903025000	2.569185000	0.576351000
C	2.316462000	4.304646000	-0.946574000
H	0.889395000	2.681007000	-1.010922000
C	4.357463000	3.855984000	0.279782000
H	4.530634000	1.897754000	1.167387000
C	3.566227000	4.724787000	-0.482877000
H	1.693244000	4.976763000	-1.541189000
H	5.334058000	4.182700000	0.644987000
H	3.926595000	5.730165000	-0.713952000
O	-1.062304000	-3.734048000	0.378715000
C	-0.178478000	-4.141006000	-0.647423000
H	-0.596781000	-5.008356000	-1.194340000
H	0.778395000	-4.439018000	-0.195147000
C	-0.038028000	-2.927342000	-1.619494000
C	1.061072000	-2.005500000	-1.124011000
H	1.043402000	-2.016547000	-0.007146000
H	2.082928000	-2.264860000	-1.417804000

C	0.120664000	-3.445620000	-3.053308000
H	-0.754068000	-4.046740000	-3.343633000
H	0.236286000	-2.649769000	-3.798694000
H	1.014688000	-4.087141000	-3.115854000

INT6 (R = Me), G = -3728.549074

C	-2.727307000	-1.038167000	-2.304269000
C	-1.355438000	-1.500879000	-1.828058000
C	-0.454384000	-1.639224000	-3.044533000
C	-1.087689000	-0.672064000	-4.058613000
C	-2.588087000	-0.906477000	-3.836809000
C	-1.071382000	-2.103245000	-0.613495000
C	-2.170277000	-2.342910000	0.407391000
H	-2.579230000	-1.418531000	0.838137000
C	0.954964000	-1.399319000	-2.558392000
C	1.740828000	-2.617389000	-2.133038000
Co	0.022498000	-0.201802000	-0.628521000
P	1.785301000	0.839255000	0.410220000
C	1.446545000	-0.136507000	-2.443707000
H	1.542780000	-3.429054000	-2.851472000
H	2.819366000	-2.403449000	-2.170747000
H	-0.532991000	-2.660603000	-3.457771000
H	0.937651000	0.716401000	-2.896430000
H	2.485800000	0.025978000	-2.161206000
H	-0.750714000	-0.854458000	-5.089446000
H	-0.842874000	0.371071000	-3.799954000
H	-2.891952000	-1.844716000	-4.329397000
H	-3.212931000	-0.100121000	-4.248297000
H	-3.024909000	-0.088044000	-1.857927000
H	-3.498098000	-1.770939000	-2.010779000
H	-3.020355000	-2.868059000	-0.071642000
P	-1.346780000	1.054065000	0.645657000
C	-0.640569000	2.765568000	0.883719000
H	-0.294777000	3.085651000	-0.109430000
H	-1.470560000	3.432885000	1.156814000
C	1.426732000	1.660913000	2.033227000
H	2.386146000	1.946434000	2.489305000
H	0.976088000	0.898697000	2.681084000
C	0.494383000	2.878842000	1.918049000
H	1.087870000	3.772223000	1.668654000
H	0.059247000	3.058157000	2.912932000
C	-2.960661000	1.480118000	-0.124400000
C	-4.182005000	0.950928000	0.321360000
C	-2.945597000	2.299324000	-1.269511000
C	-5.364712000	1.229000000	-0.371577000
H	-4.219556000	0.314938000	1.207346000
C	-4.128910000	2.579210000	-1.955221000
H	-2.003342000	2.701159000	-1.649749000
C	-5.341586000	2.039705000	-1.510106000
H	-6.308347000	0.806925000	-0.017824000
H	-4.102859000	3.213394000	-2.844445000
H	-6.266765000	2.251578000	-2.051187000
C	-1.755049000	0.593184000	2.379429000
C	-2.589131000	1.400147000	3.174534000
C	-1.145173000	-0.531942000	2.950666000
C	-2.813106000	1.071815000	4.513314000
H	-3.070889000	2.284026000	2.749202000
C	-1.368674000	-0.860629000	4.290101000
H	-0.491221000	-1.149587000	2.338403000
C	-2.204906000	-0.059562000	5.072530000

H	-3.464568000	1.701703000	5.123774000
H	-0.891701000	-1.746010000	4.716525000
H	-2.384430000	-0.313992000	6.119952000
C	3.061463000	-0.400649000	0.870420000
C	4.131933000	-0.705182000	0.011493000
C	2.893605000	-1.161913000	2.042583000
C	5.008865000	-1.750251000	0.314709000
H	4.298331000	-0.121245000	-0.896061000
C	3.773814000	-2.203813000	2.343683000
H	2.065197000	-0.963215000	2.724549000
C	4.830370000	-2.505217000	1.478254000
H	5.835892000	-1.973327000	-0.363408000
H	3.627347000	-2.785899000	3.256457000
H	5.514659000	-3.324234000	1.711564000
C	2.699258000	2.196057000	-0.440193000
C	2.084612000	2.914243000	-1.478436000
C	3.966541000	2.602806000	0.013991000
C	2.725269000	4.013517000	-2.058012000
H	1.097524000	2.619786000	-1.839738000
C	4.608271000	3.697647000	-0.569789000
H	4.459624000	2.061917000	0.825197000
C	3.989021000	4.404989000	-1.606752000
H	2.234439000	4.562882000	-2.864748000
H	5.594838000	4.000670000	-0.211055000
H	4.491957000	5.261915000	-2.061327000
O	-1.628843000	-3.168611000	1.427262000
C	-0.276265000	-3.486541000	1.131353000
H	-0.075550000	-4.521572000	1.450062000
H	0.412643000	-2.821073000	1.687962000
C	-0.090544000	-3.284359000	-0.382570000
C	1.394611000	-3.068667000	-0.704523000
H	1.795494000	-2.320907000	-0.012277000
H	1.935758000	-4.004507000	-0.487738000
C	-0.646758000	-4.518127000	-1.132140000
H	-1.672971000	-4.750232000	-0.811641000
H	-0.659605000	-4.368736000	-2.220236000
H	-0.019215000	-5.398009000	-0.918140000

8. NMR spectra

^{1}H NMR (300 MHz, $CDCl_3$)

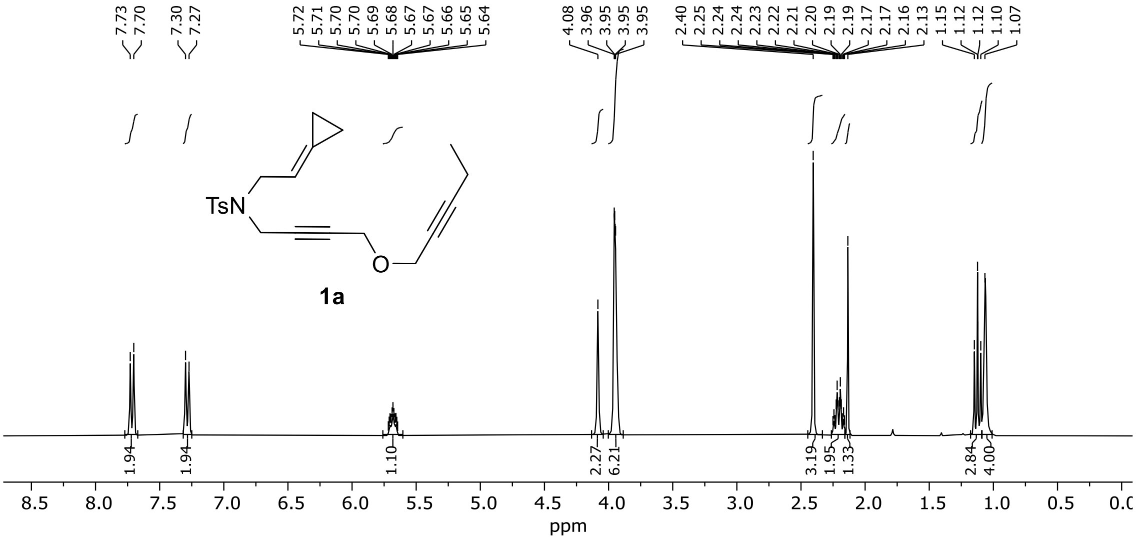

^{13}C NMR (75 MHz, $CDCl_3$)

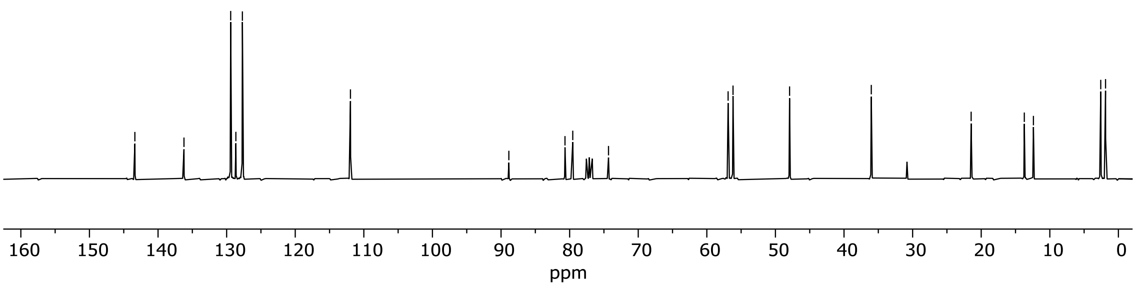

DEPT (75 MHz, $CDCl_3$)

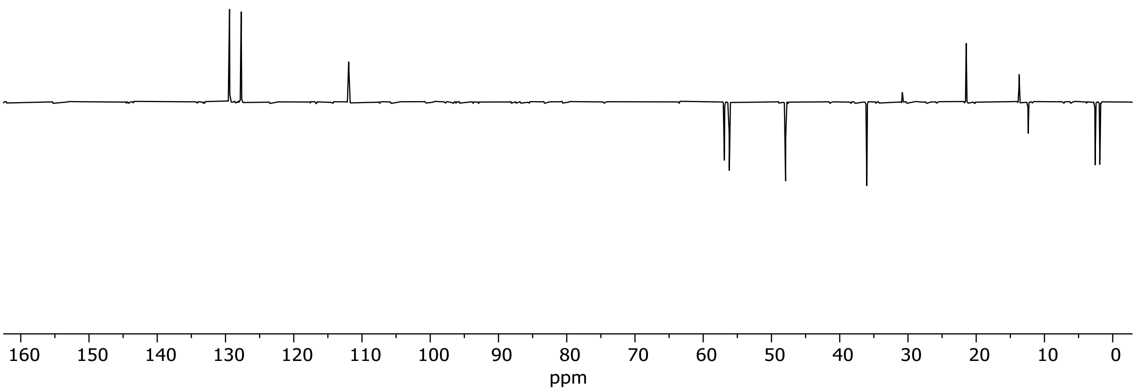

^{1}H NMR (300 MHz, $CDCl_3$)

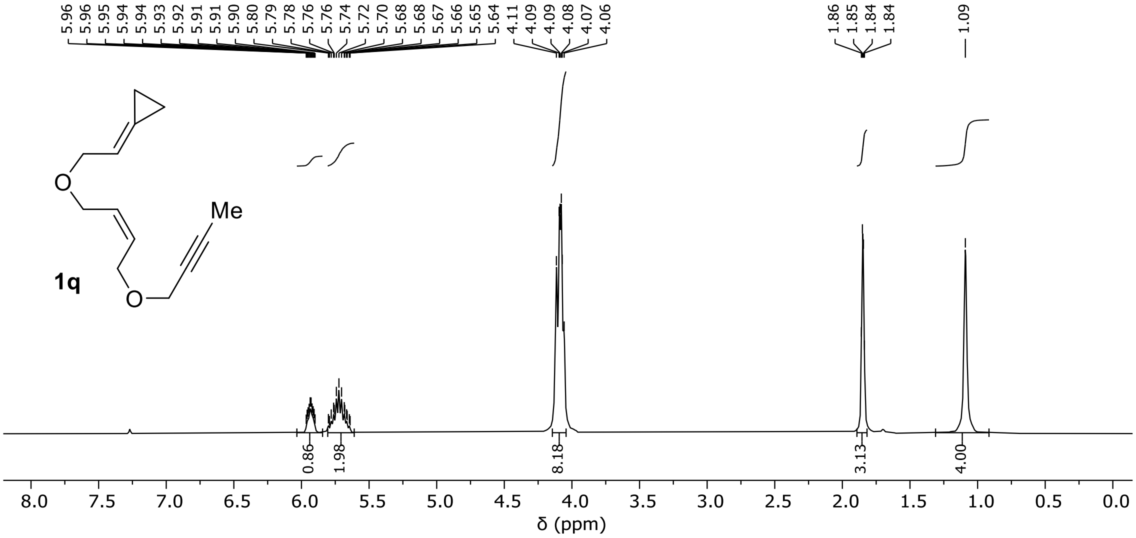

^{13}C NMR (75 MHz, $CDCl_3$)

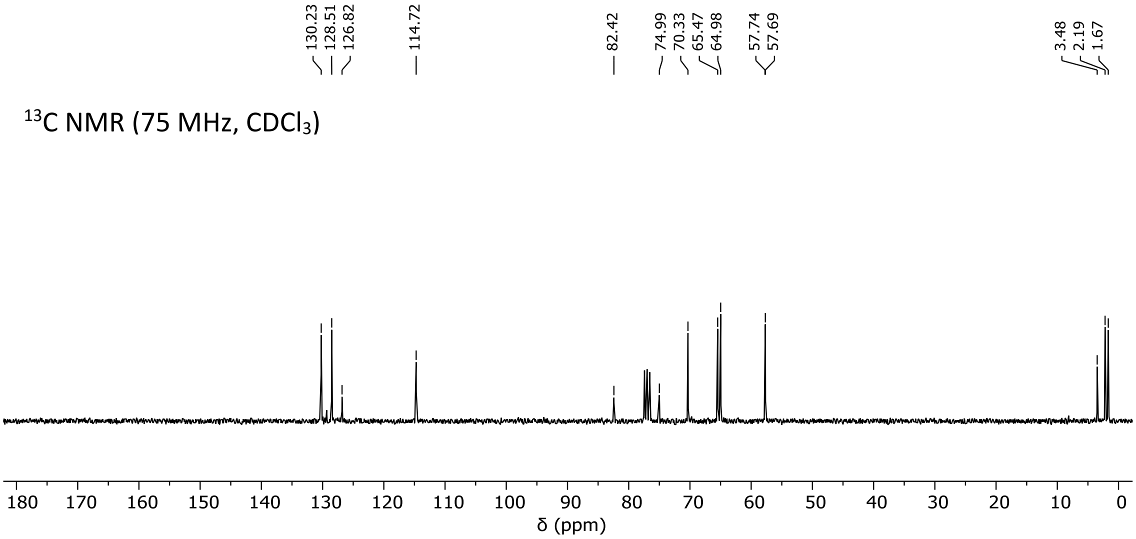

DEPT (75 MHz, $CDCl_3$)

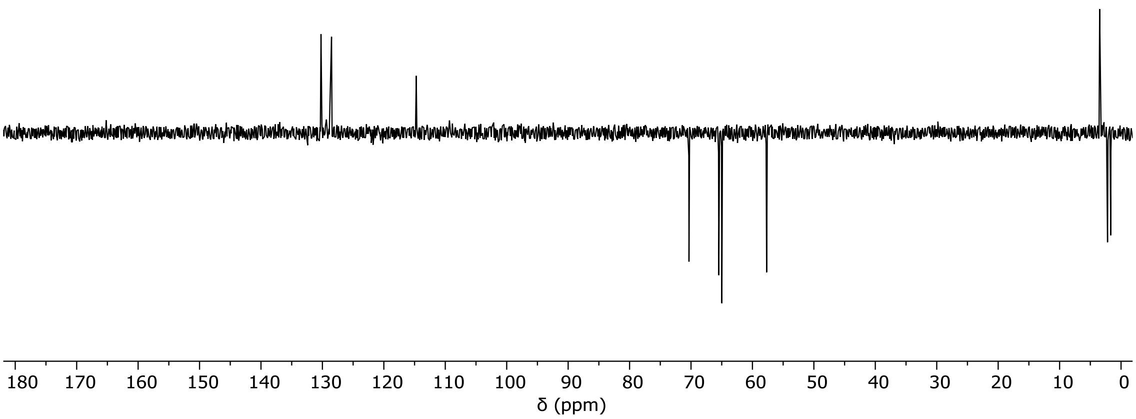

^{1}H NMR (300 MHz, $CDCl_3$)

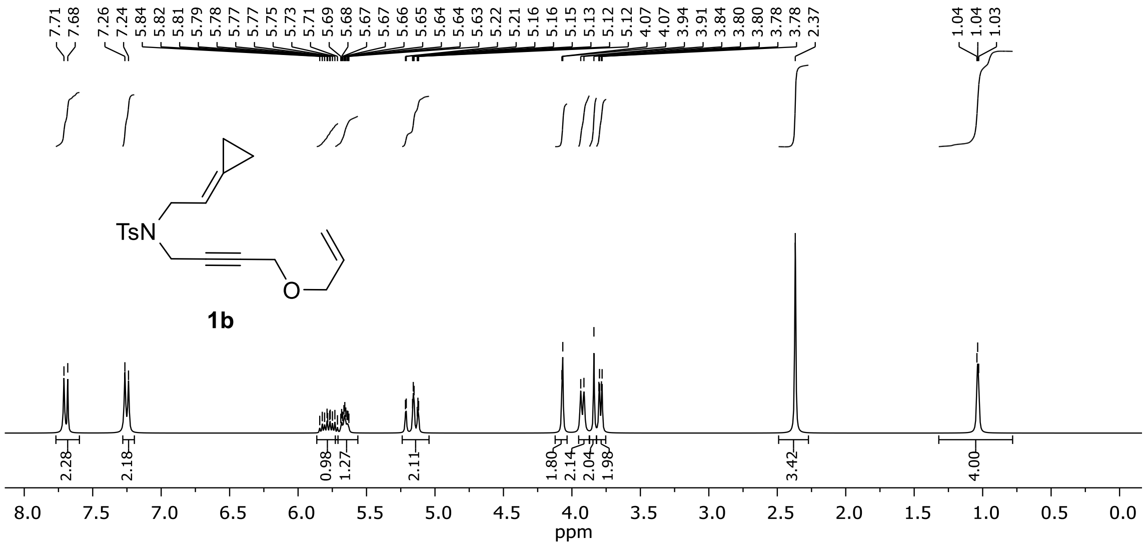

^{13}C NMR (75 MHz, $CDCl_3$)

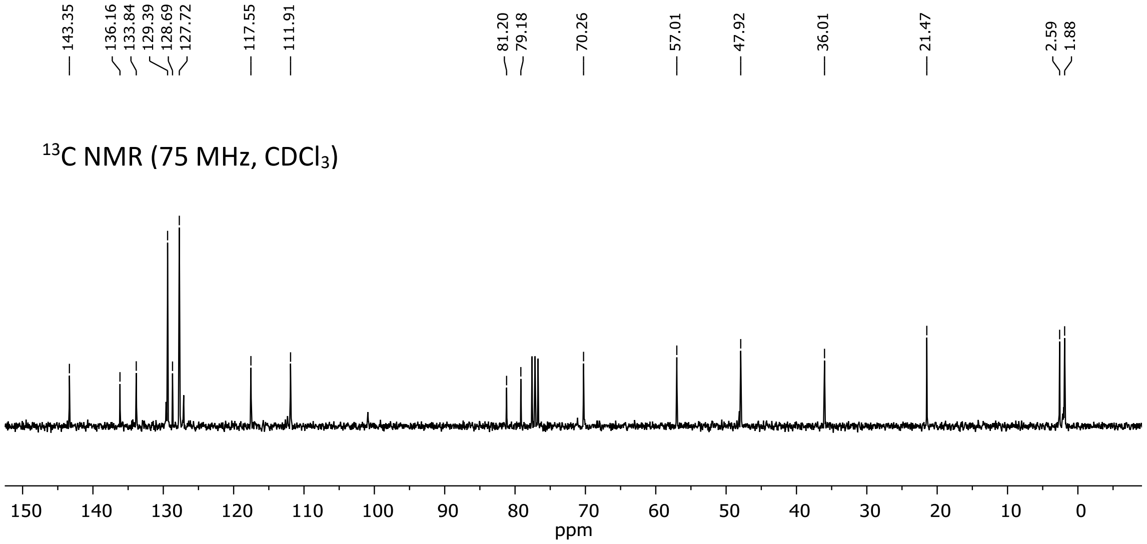

DEPT (75 MHz, $CDCl_3$)

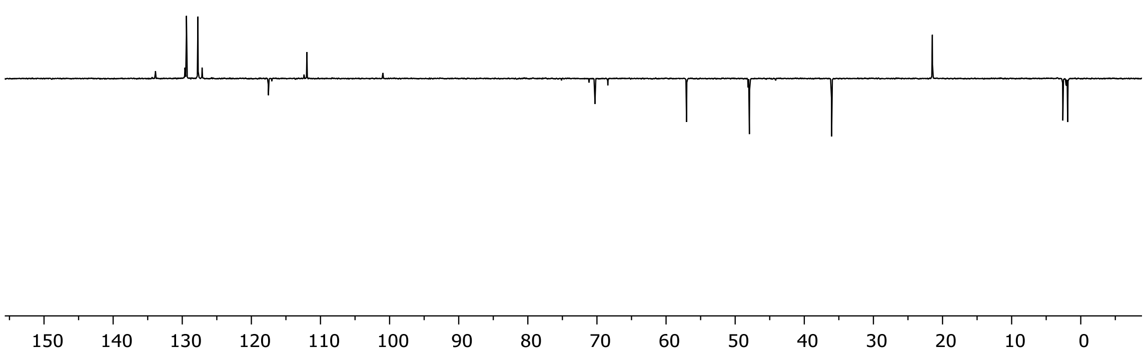

^{1}H NMR (300 MHz, $CDCl_3$)

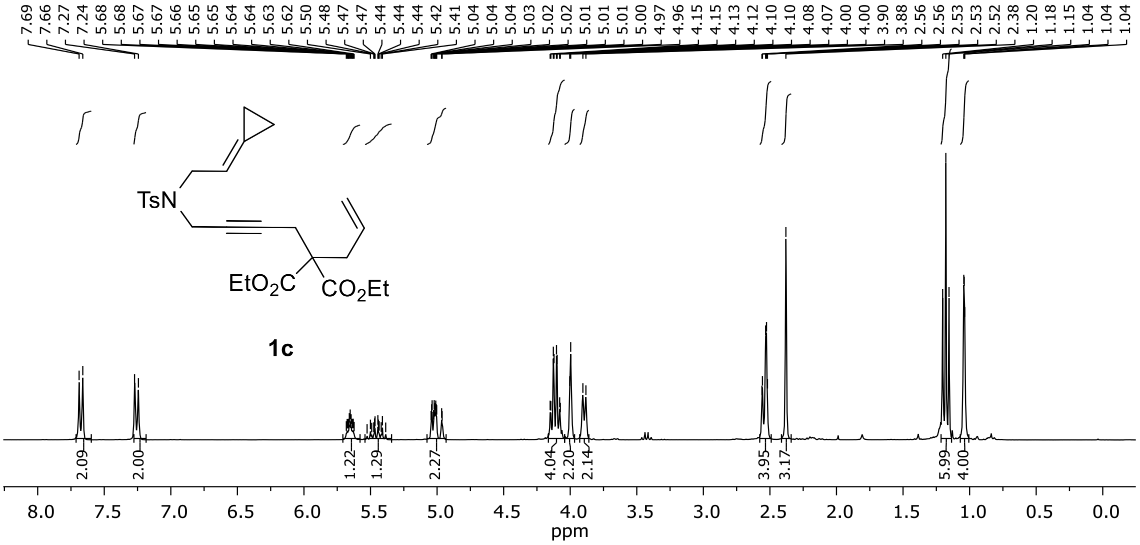

^{13}C NMR (75 MHz, $CDCl_3$)

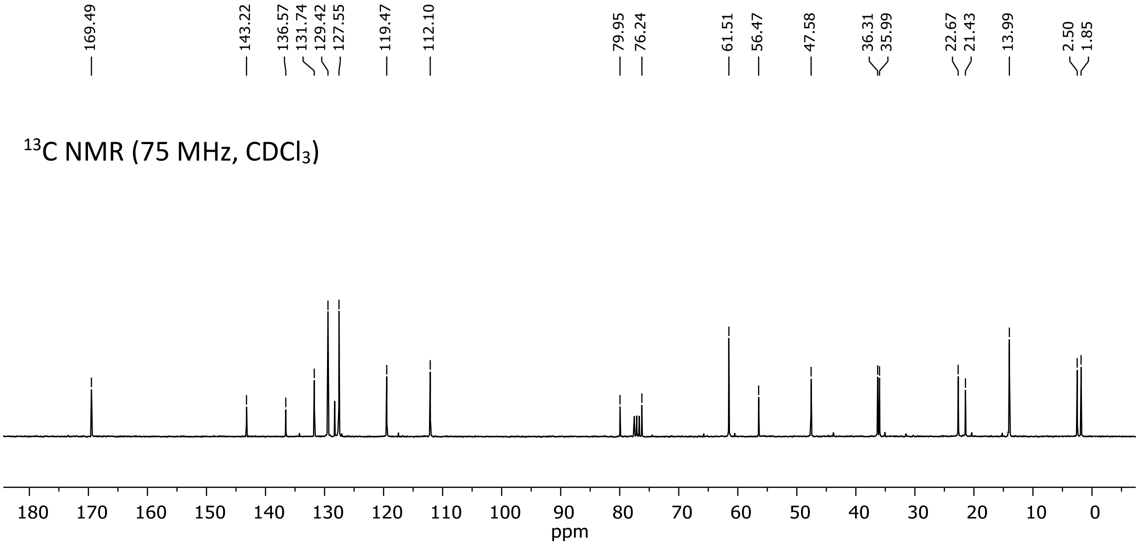

DEPT (75 MHz, $CDCl_3$)

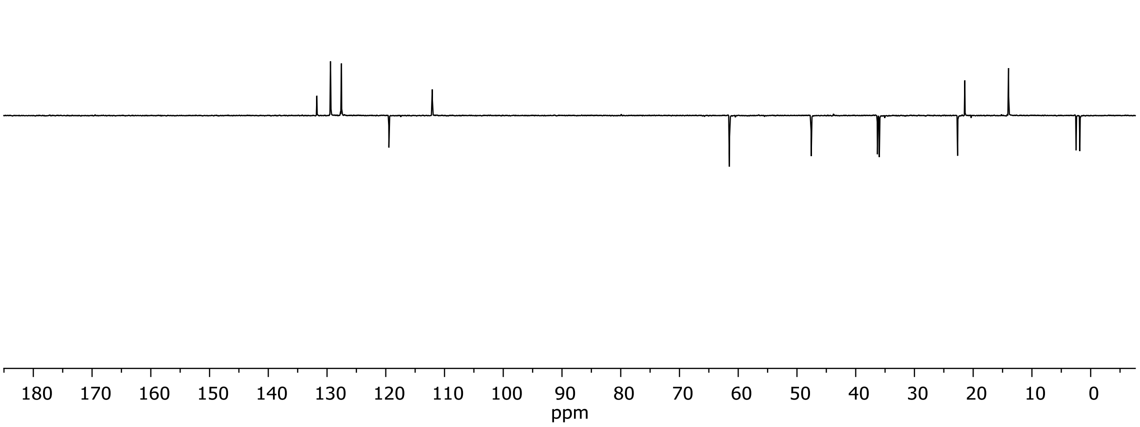

^{1}H NMR (300 MHz, $CDCl_3$)

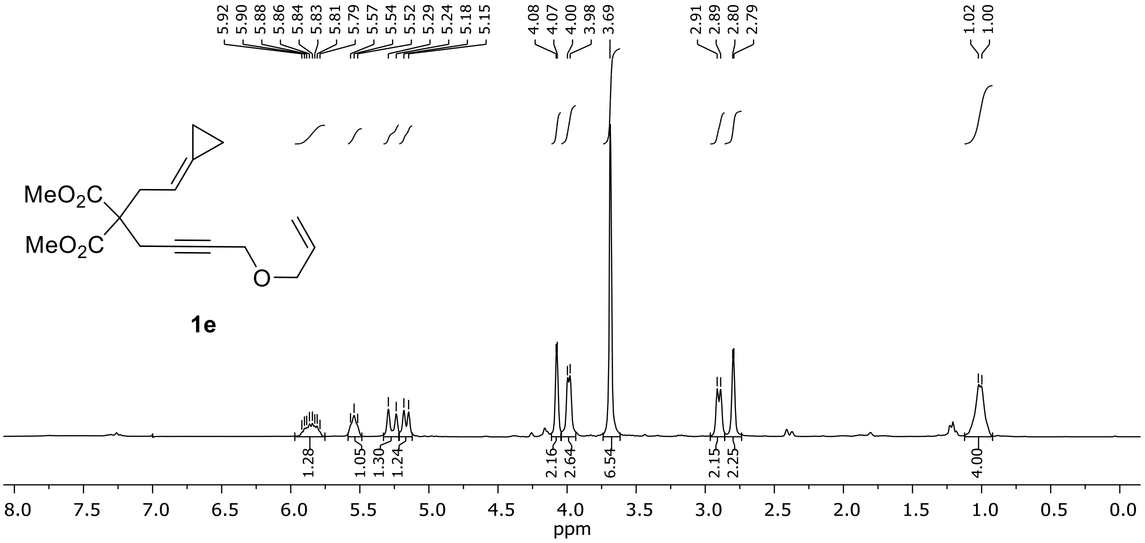

^{13}C NMR (75 MHz, $CDCl_3$)

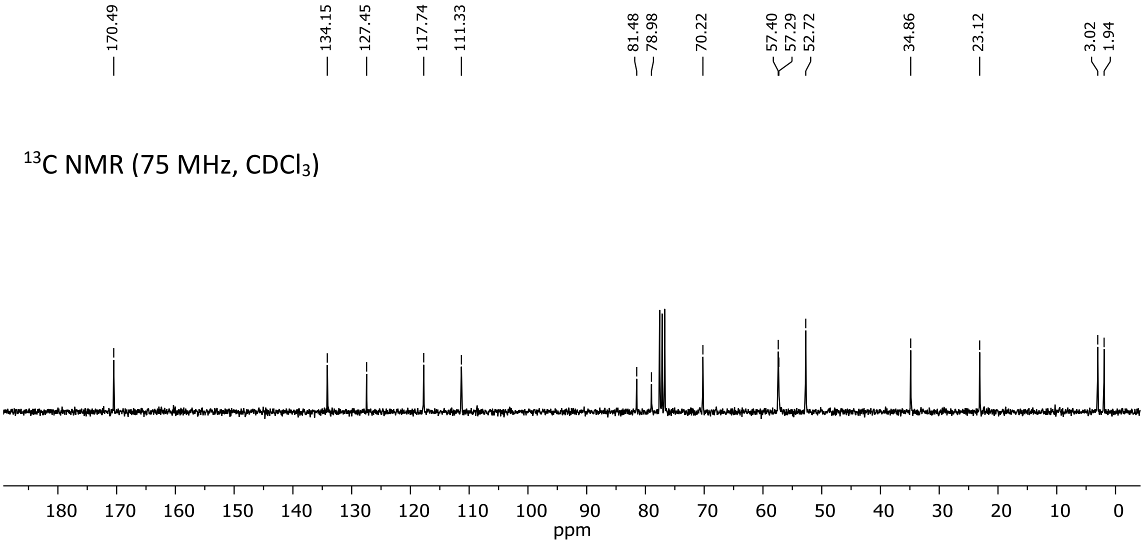

DEPT (75 MHz, $CDCl_3$)

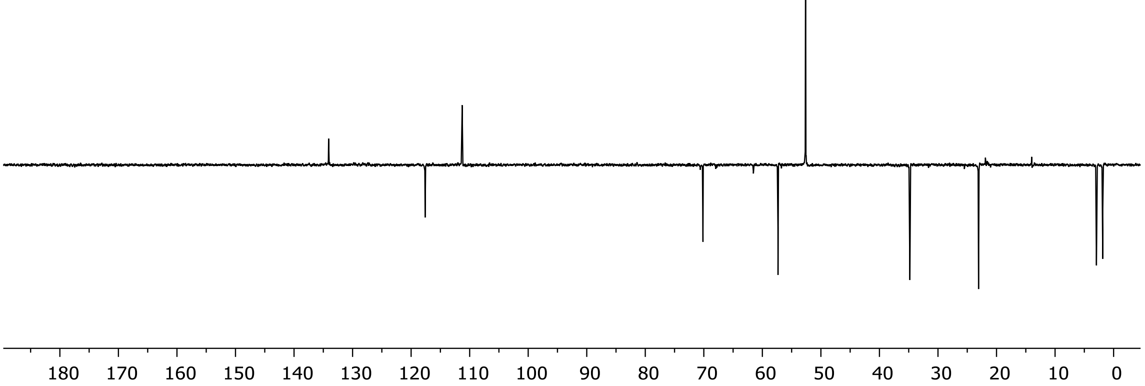

^1H NMR (300 MHz, $CDCl_3$)

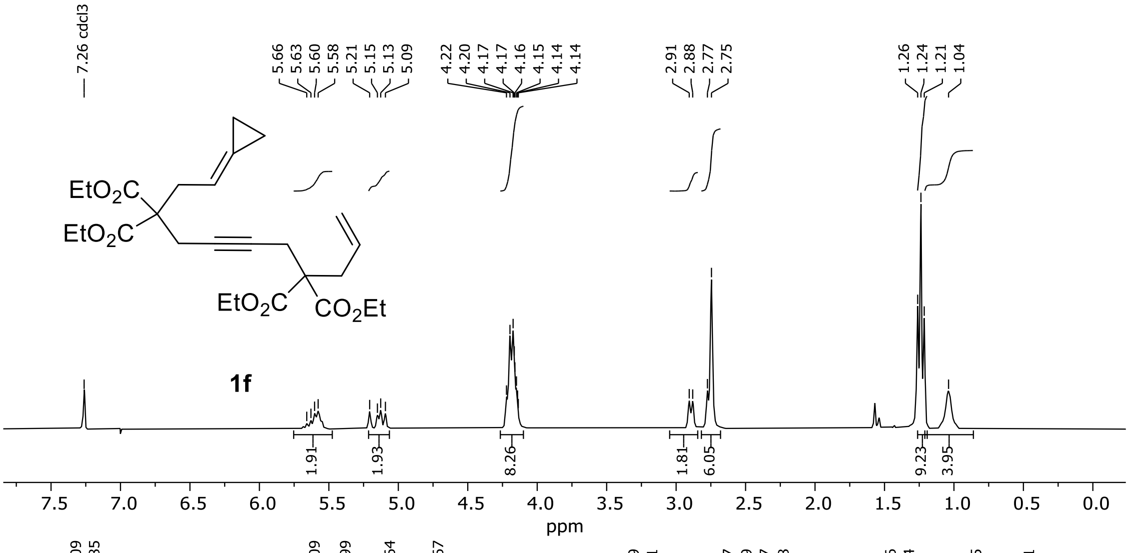

^{13}C NMR (75 MHz, $CDCl_3$)

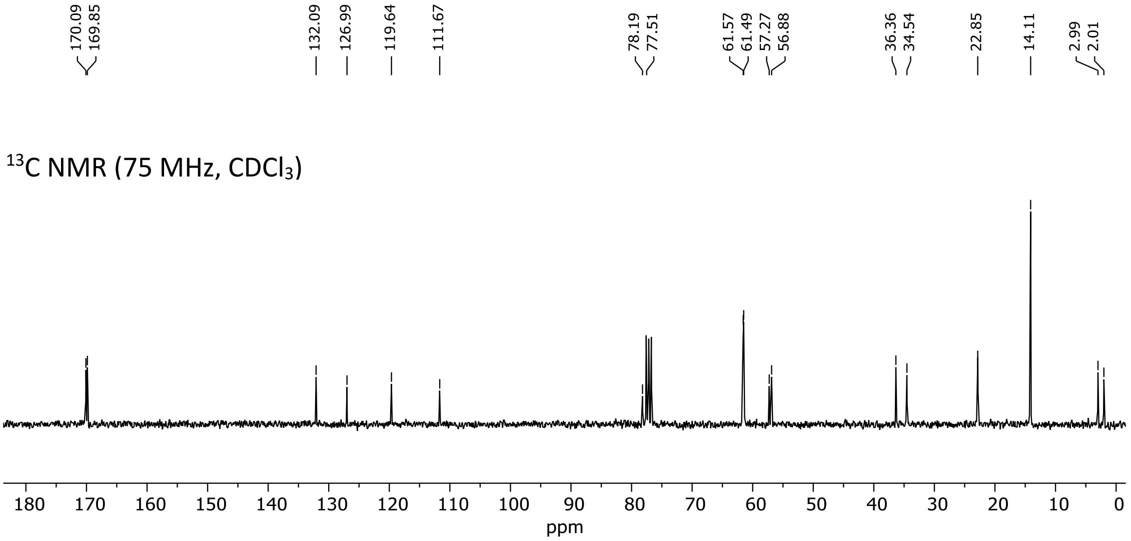

DEPT (75 MHz, $CDCl_3$)

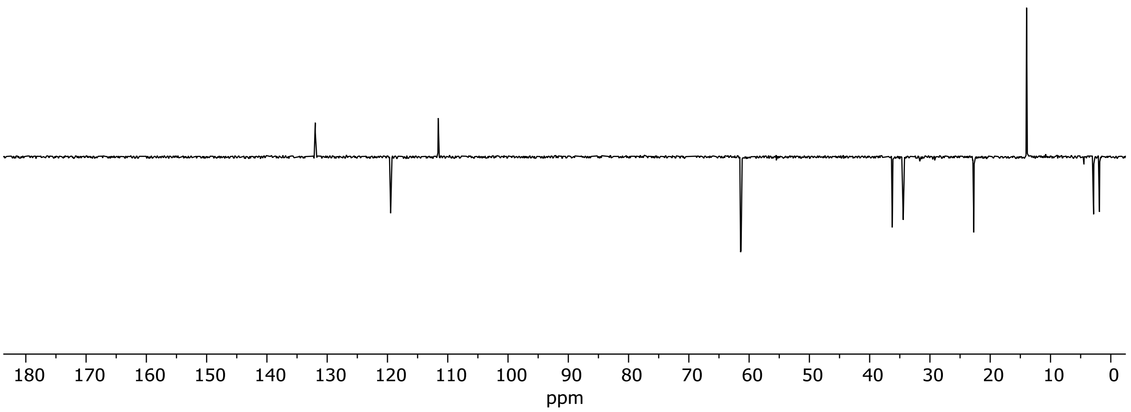

^{1}H NMR (300 MHz, $CDCl_3$)

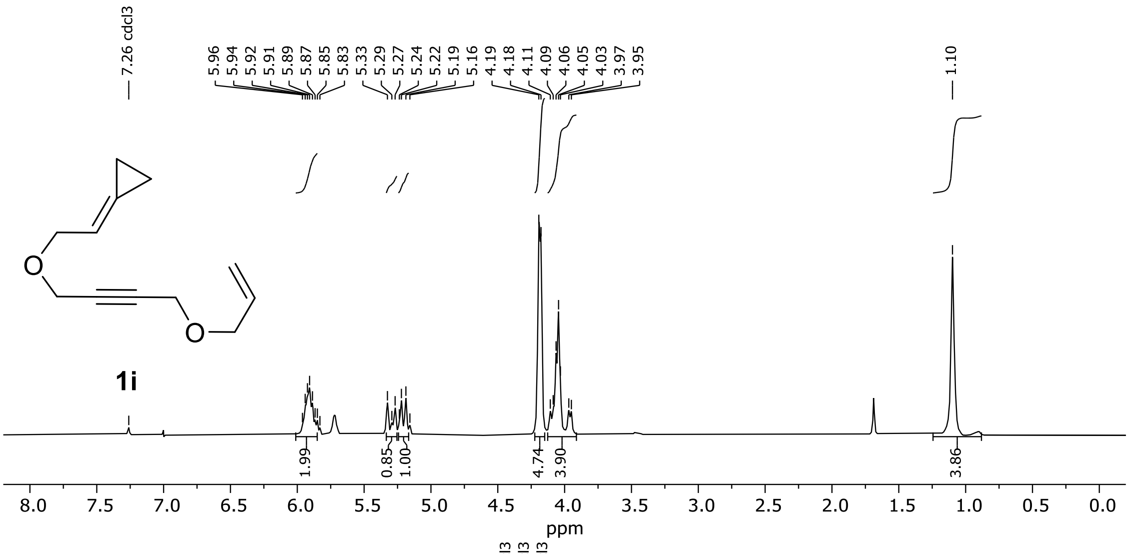

^{13}C NMR (75 MHz, $CDCl_3$)

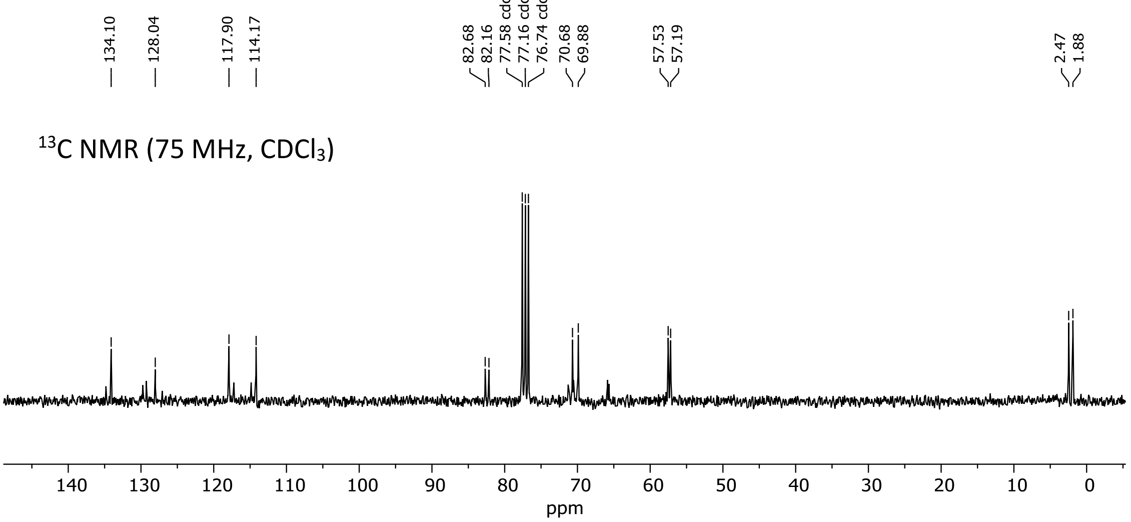

DEPT (75 MHz, $CDCl_3$)

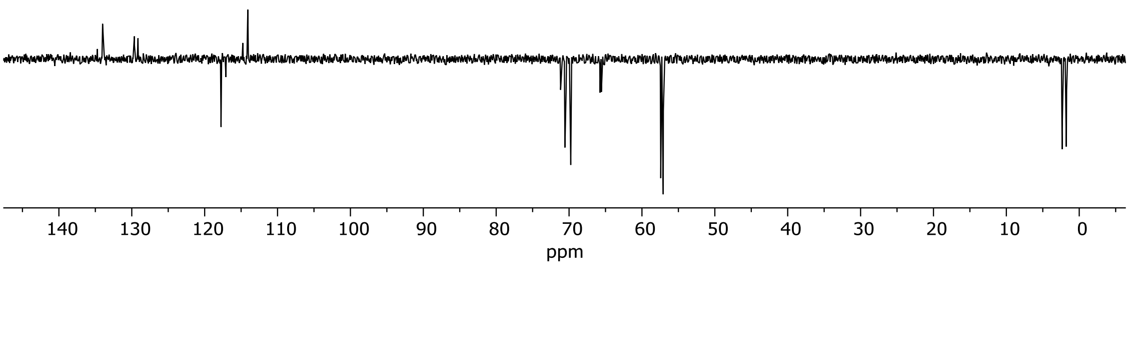

^{1}H NMR (300 MHz, $CDCl_3$)

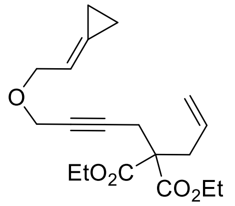

1h

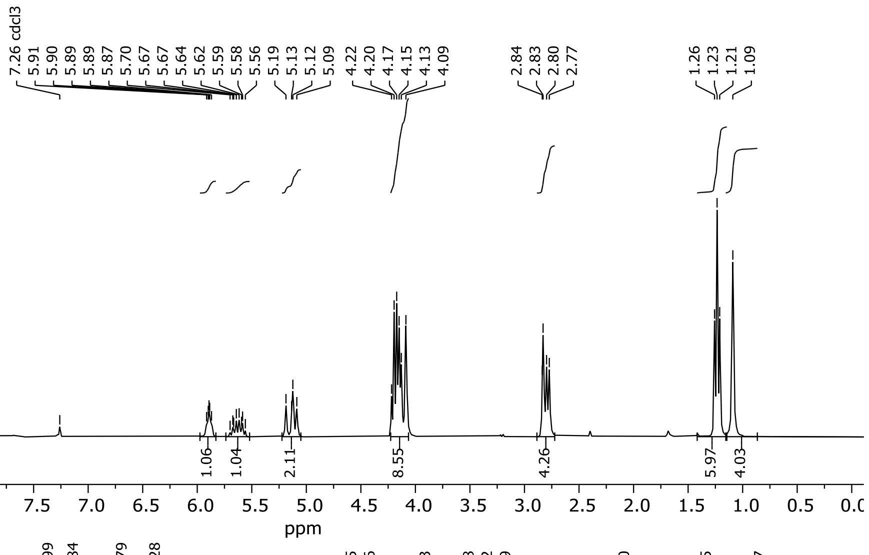

^{13}C NMR (75 MHz, $CDCl_3$)

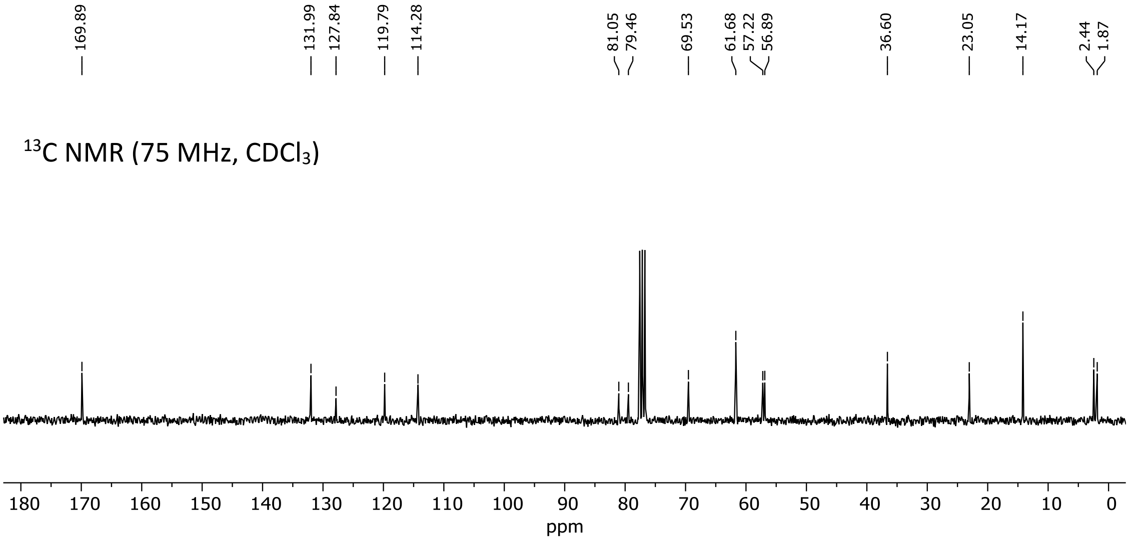

DEPT (75 MHz, $CDCl_3$)

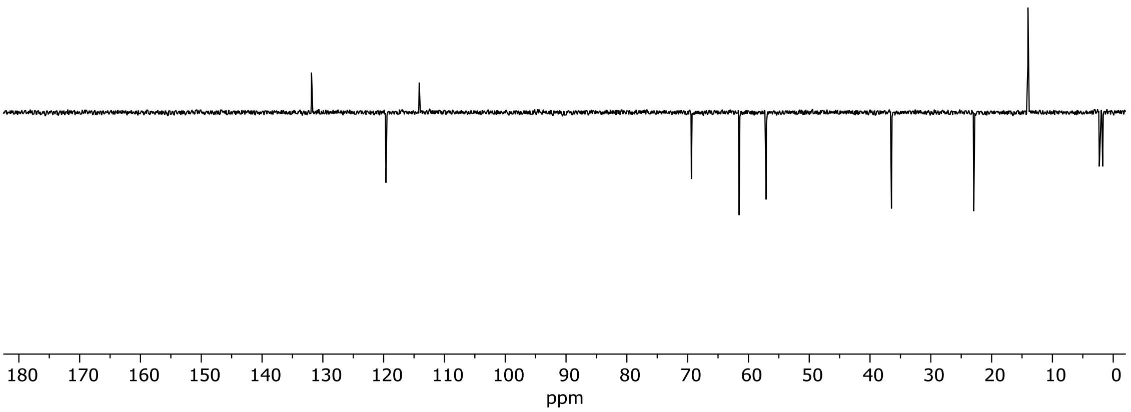

^{1}H NMR (300 MHz, $CDCl_3$)

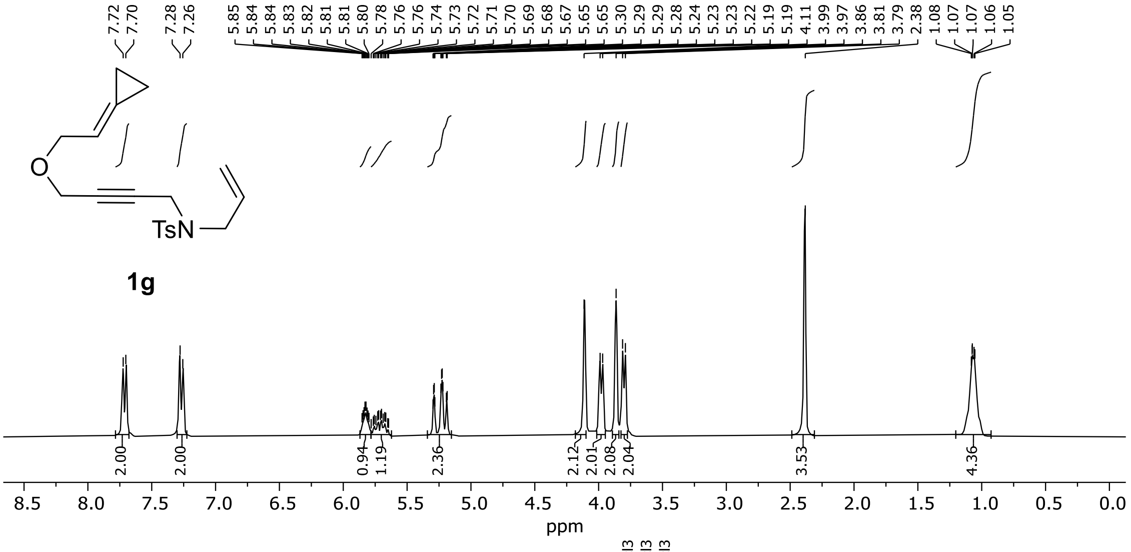

^{13}C NMR (75 MHz, $CDCl_3$)

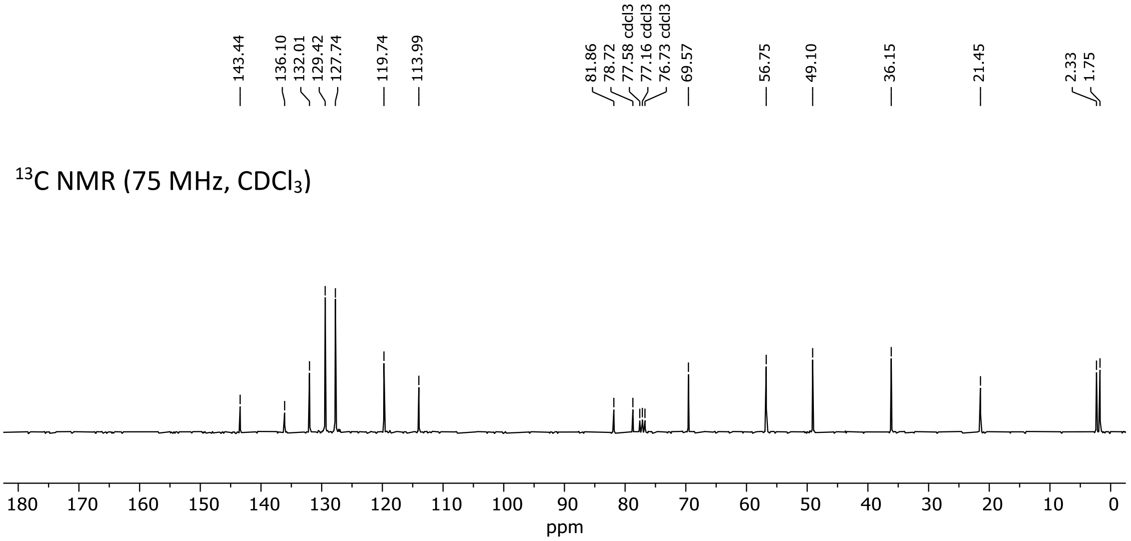

DEPT (75 MHz, $CDCl_3$)

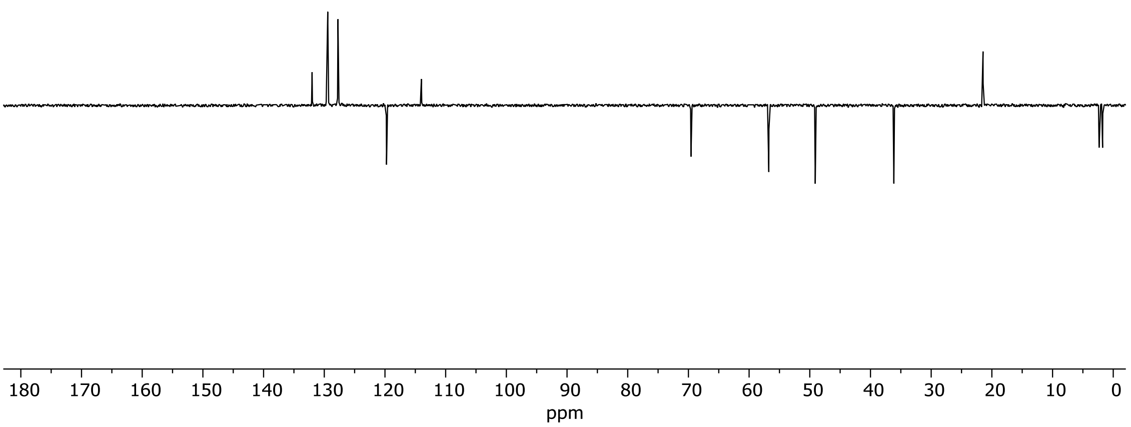

^{1}H NMR (300 MHz, $CDCl_3$)

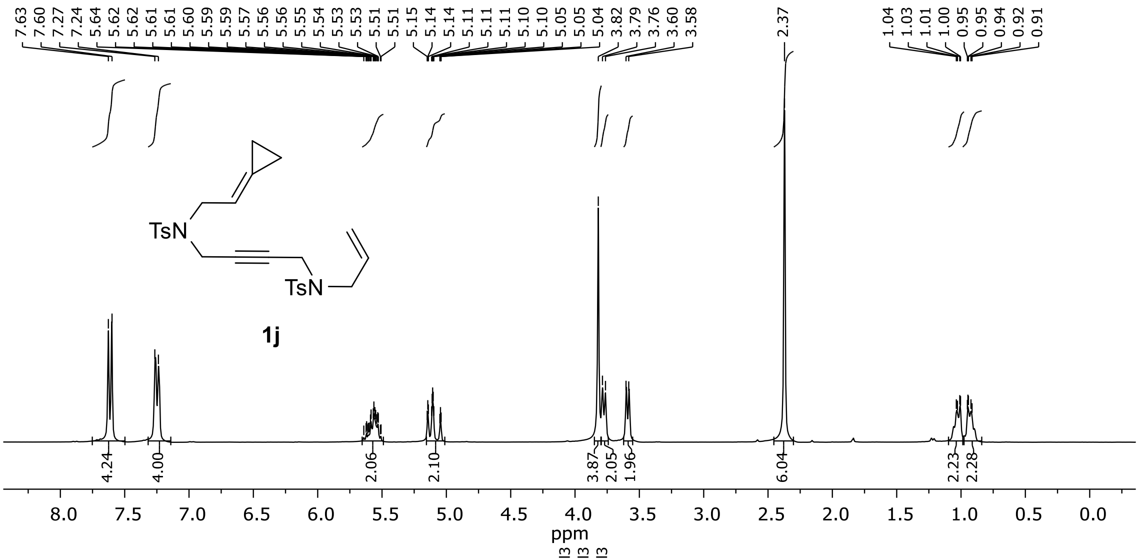

^{13}C NMR (75 MHz, $CDCl_3$)

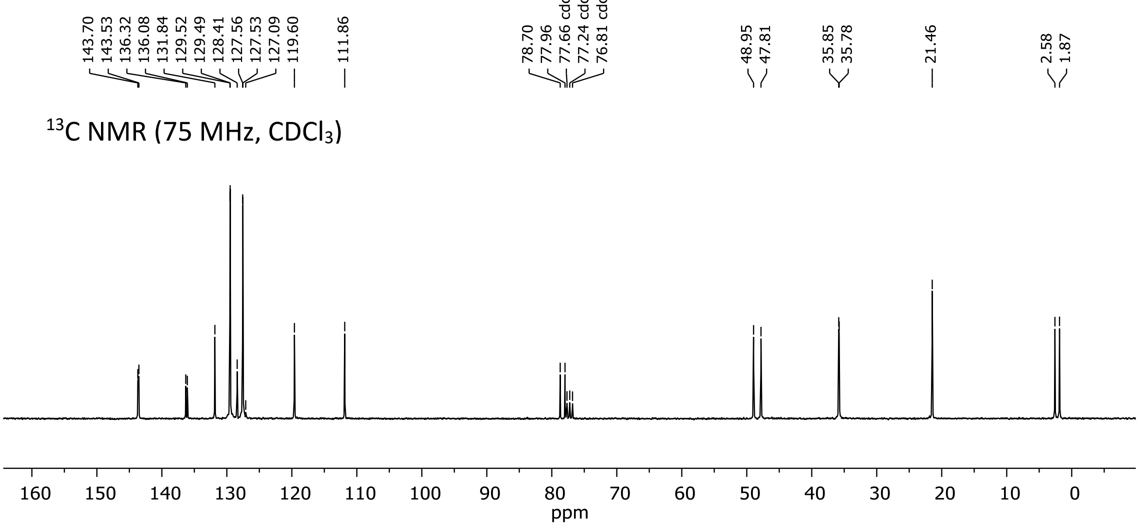

DEPT (75 MHz, $CDCl_3$)

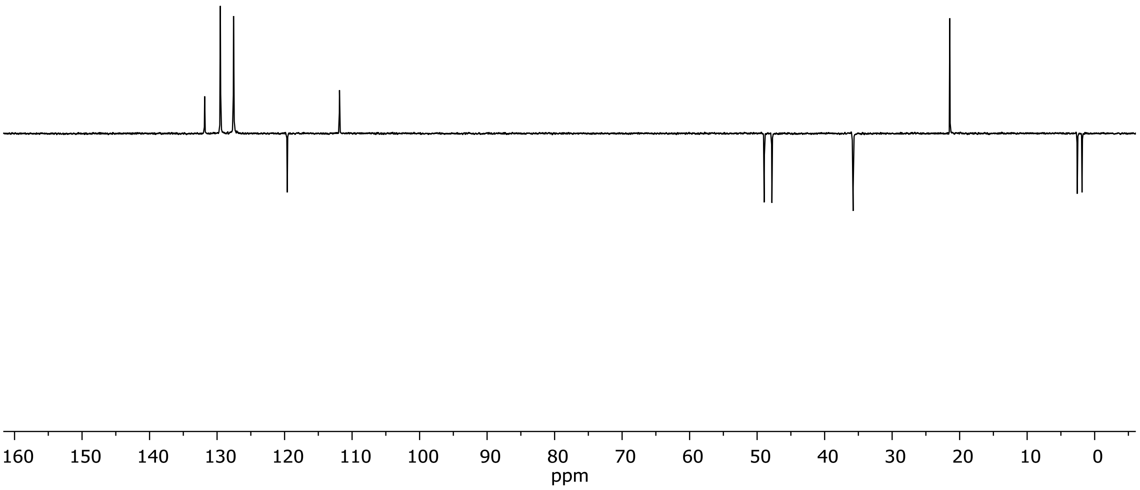

^1H NMR (300 MHz, $CDCl_3$)

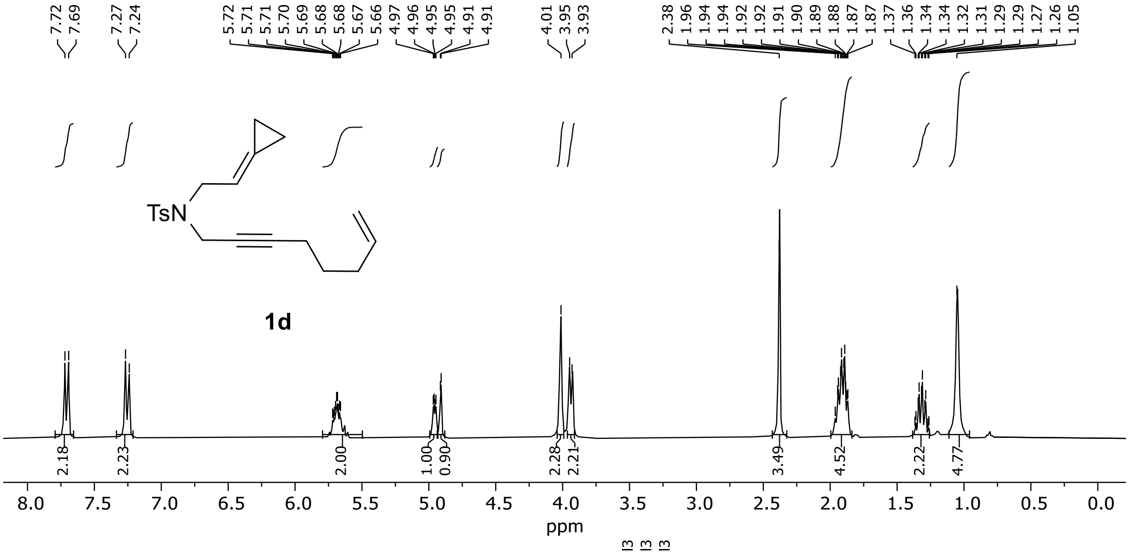

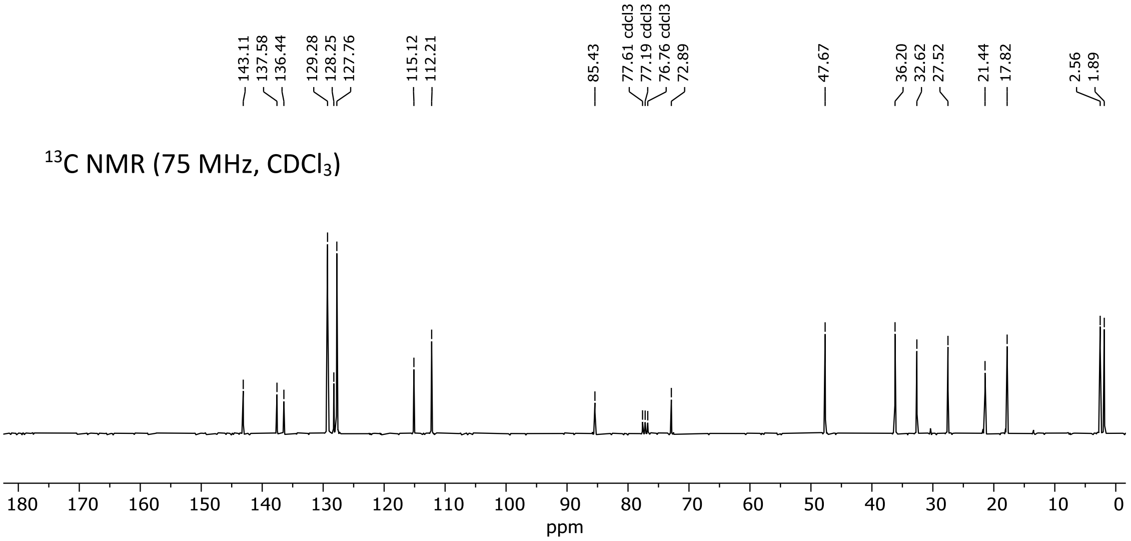

^{13}C NMR (75 MHz, $CDCl_3$)

DEPT (75 MHz, $CDCl_3$)

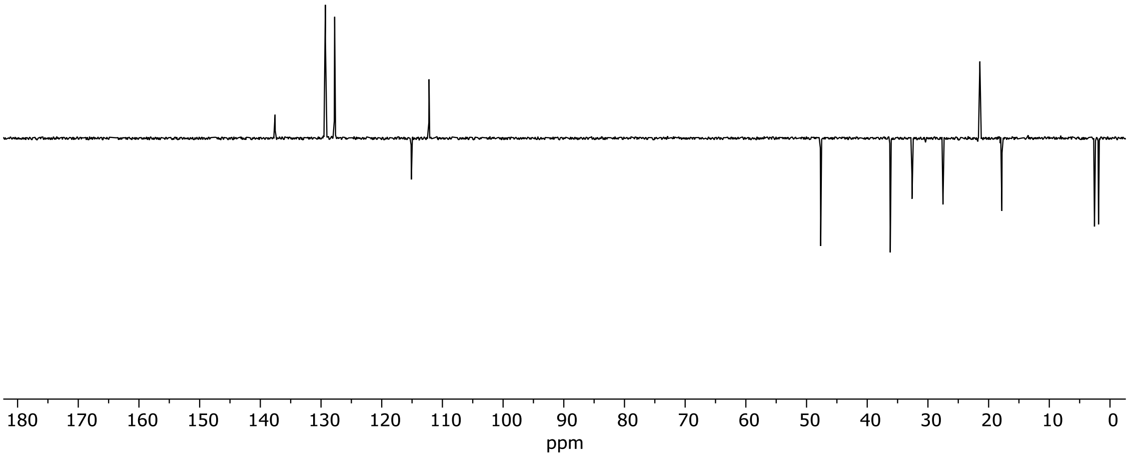

^{1}H NMR (300 MHz, $CDCl_3$)

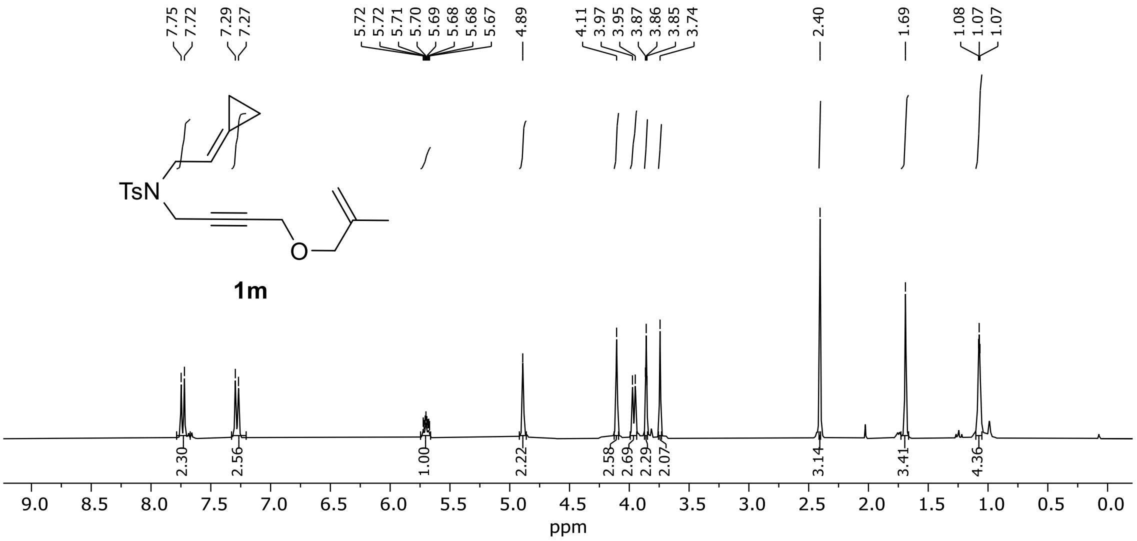

^{13}C NMR (75 MHz, $CDCl_3$)

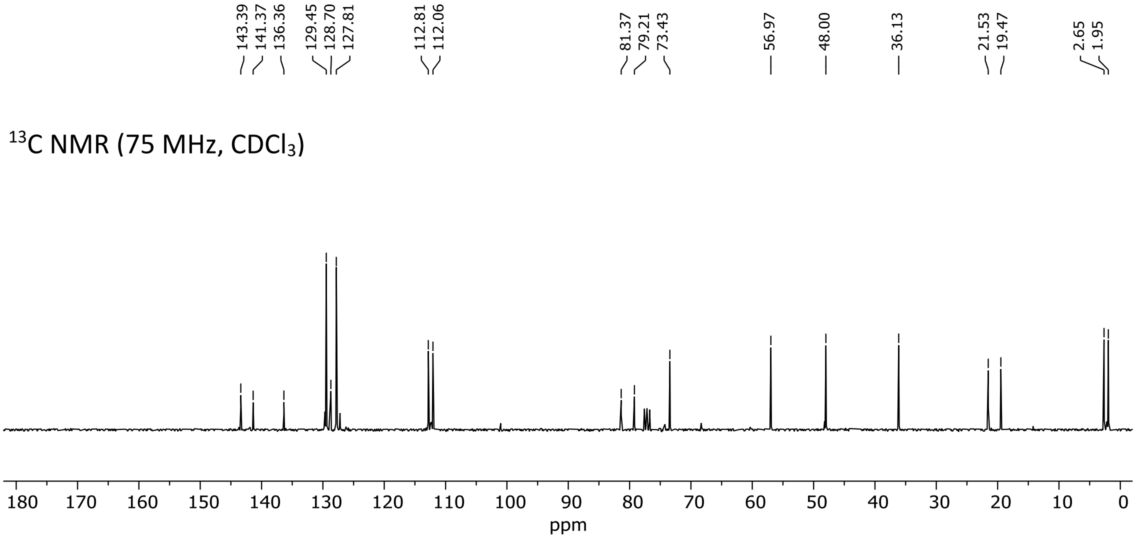

DEPT (75 MHz, $CDCl_3$)

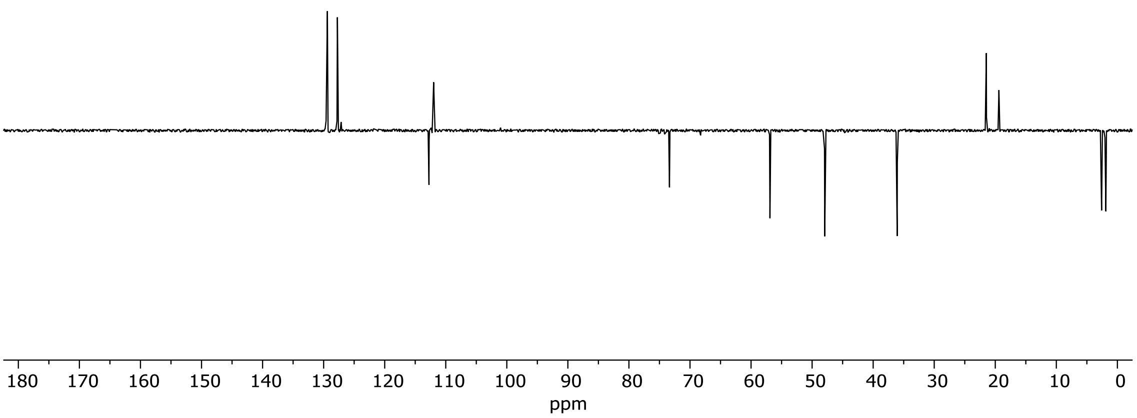

^{1}H NMR (300 MHz, $CDCl_3$)

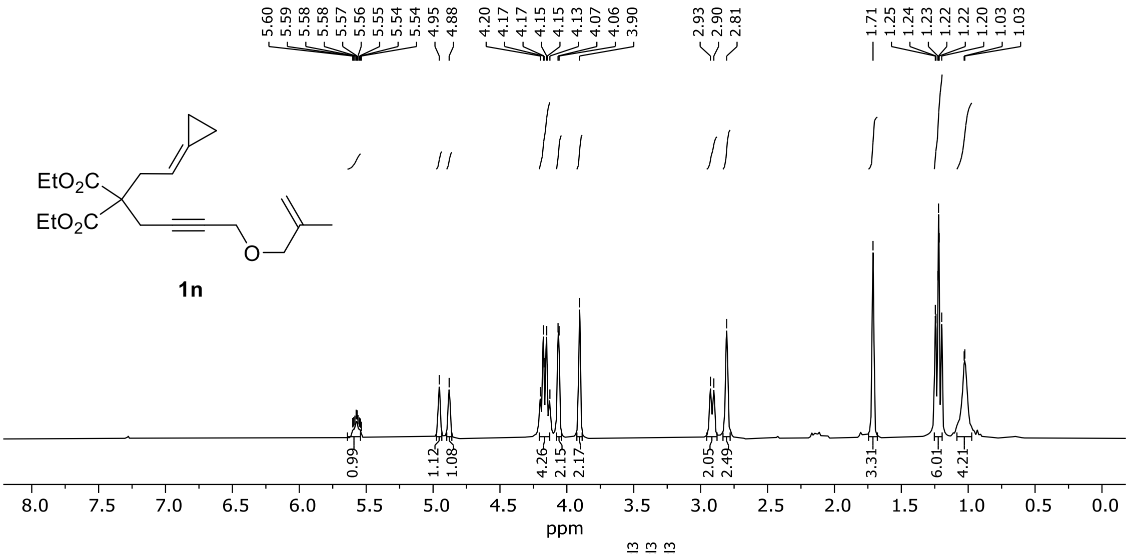

^{13}C NMR (75 MHz, $CDCl_3$)

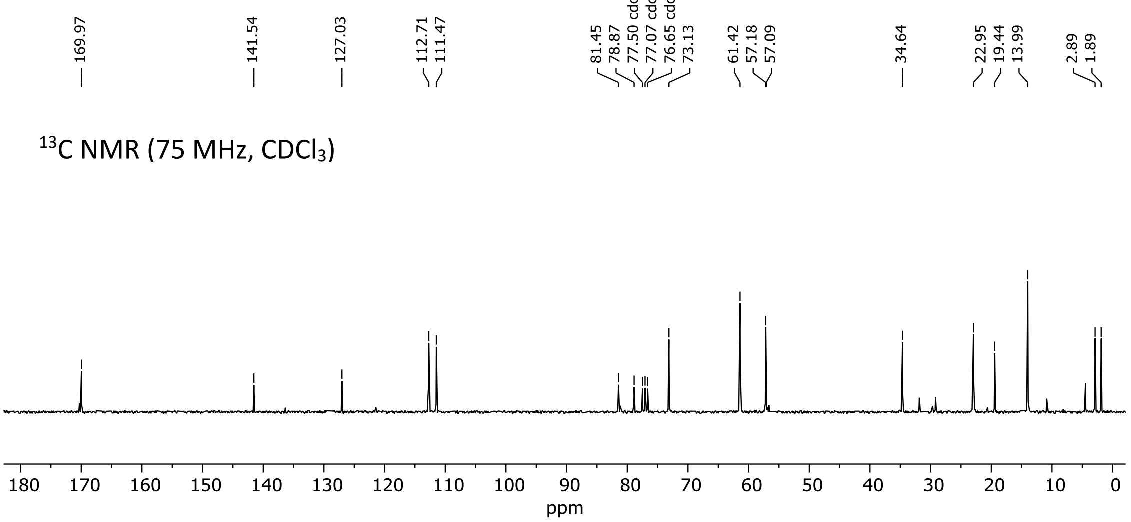

DEPT (75 MHz, $CDCl_3$)

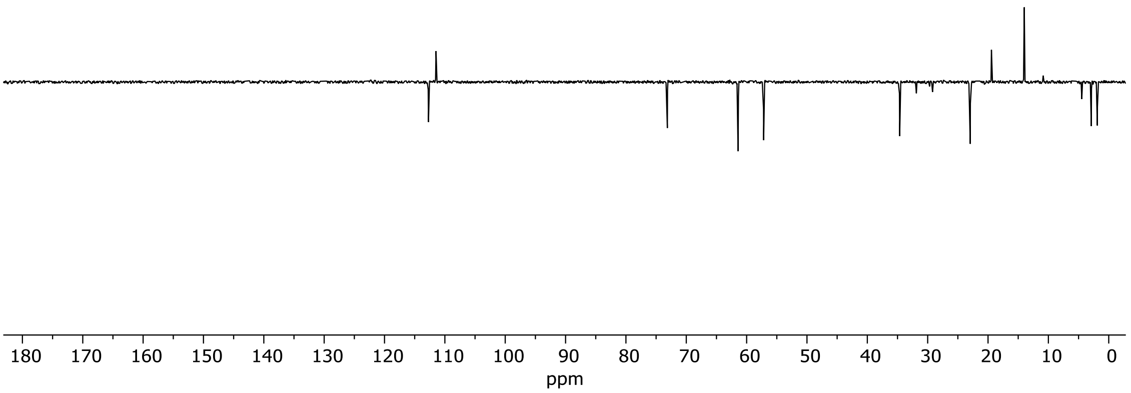

^{1}H NMR (300 MHz, $CDCl_3$)

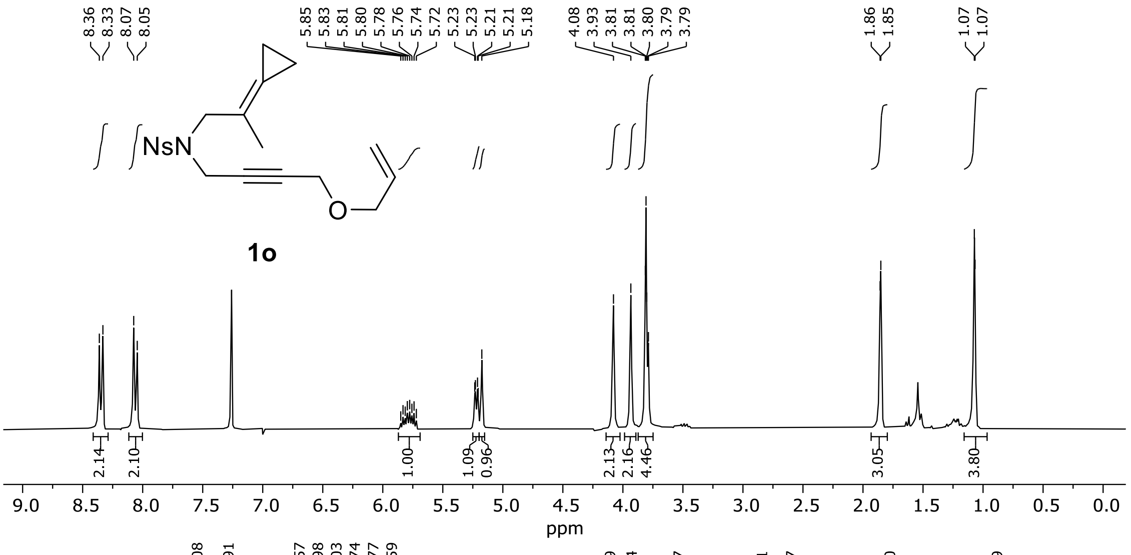

^{13}C NMR (75 MHz, $CDCl_3$)

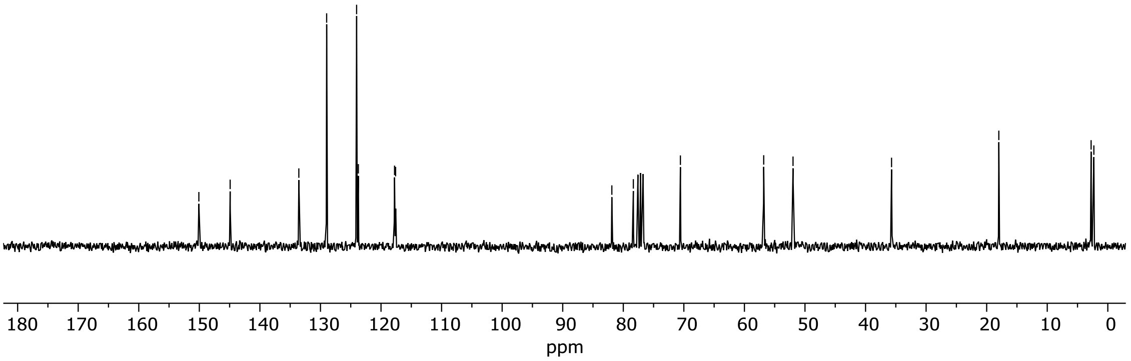

DEPT (75 MHz, $CDCl_3$)

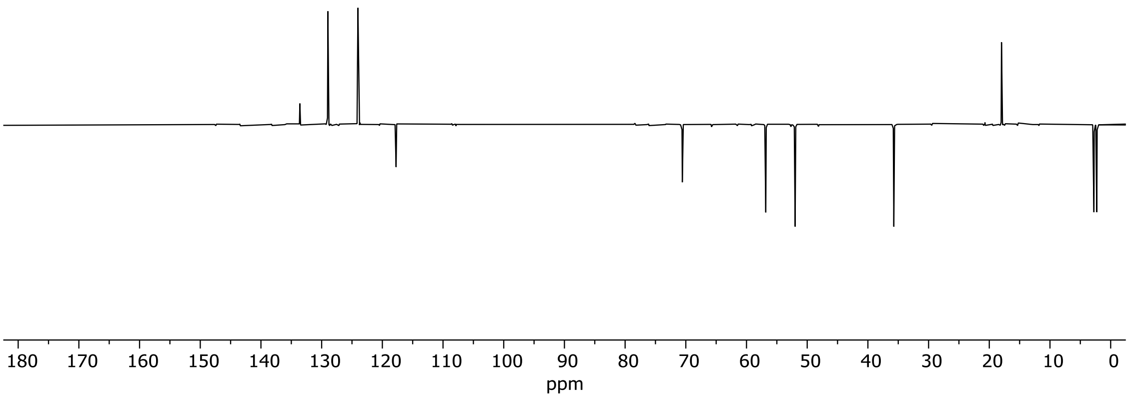

^{1}H NMR (300 MHz, $CDCl_3$)

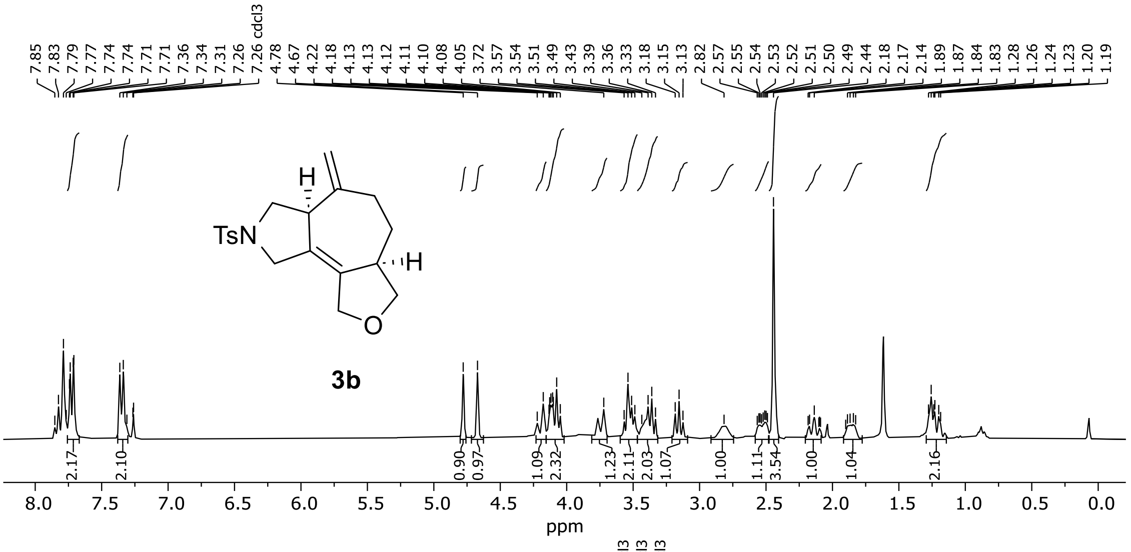

^{13}C NMR (75 MHz, $CDCl_3$)

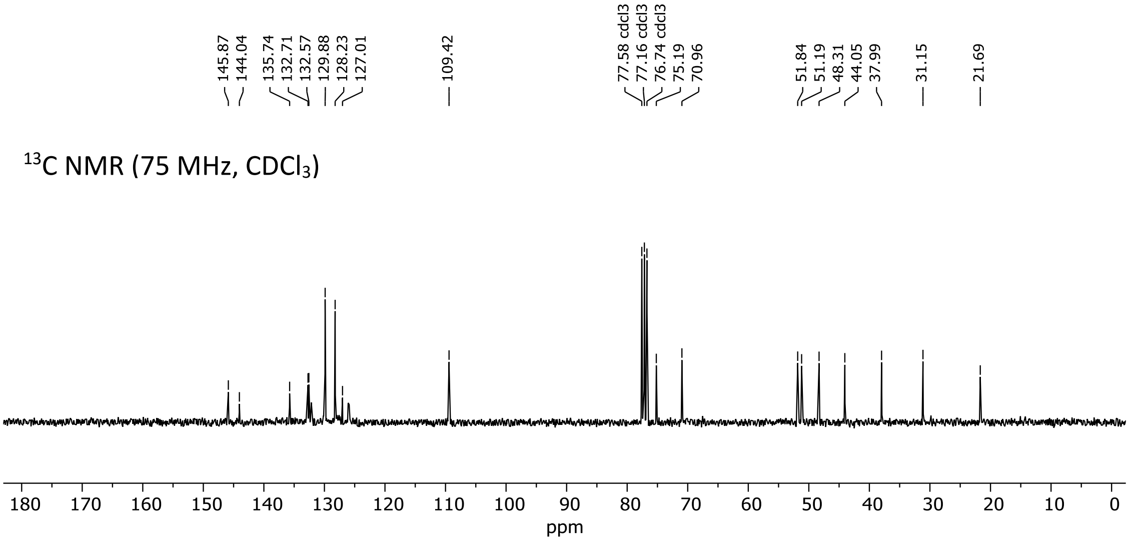

DEPT (75 MHz, $CDCl_3$)

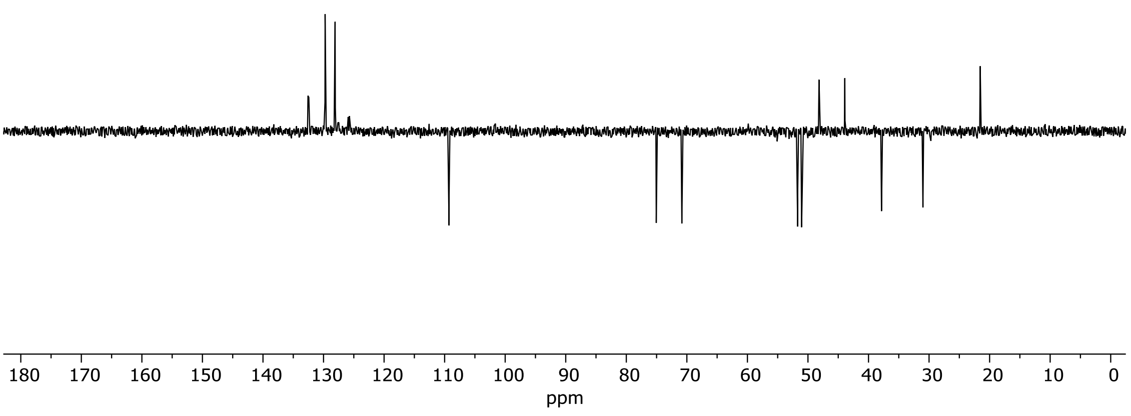

^{1}H NMR (500 MHz, $CDCl_3$)

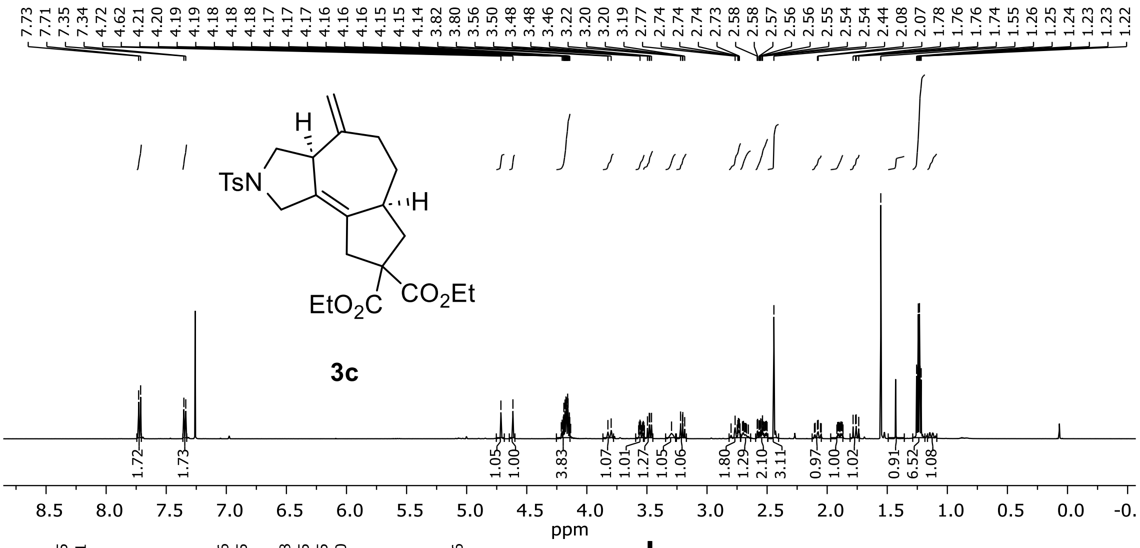

3c

^{13}C NMR 126 MHz, $CDCl_3$)

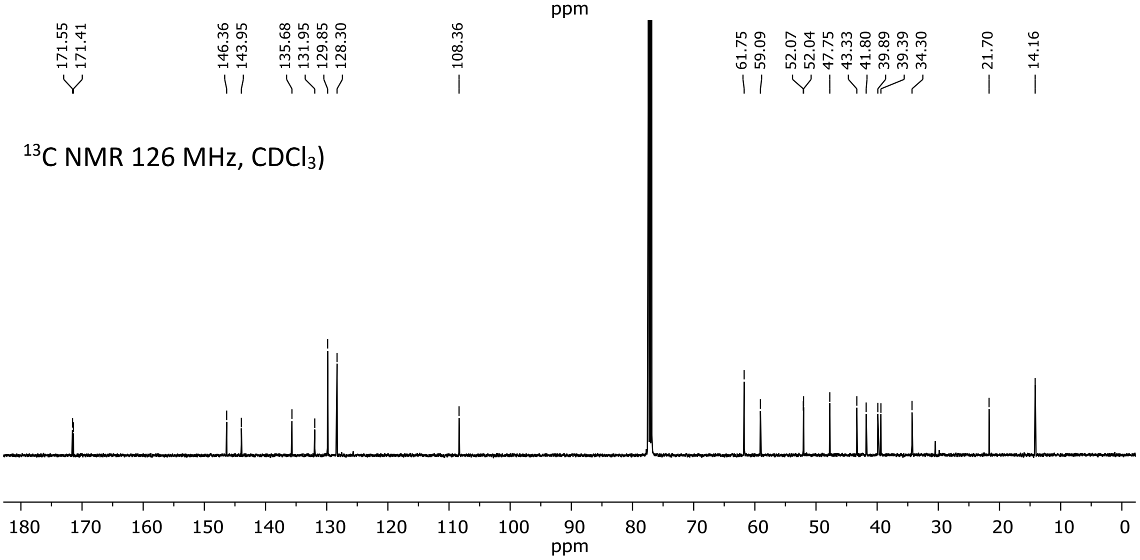

DEPT (75 MHz, $CDCl_3$)

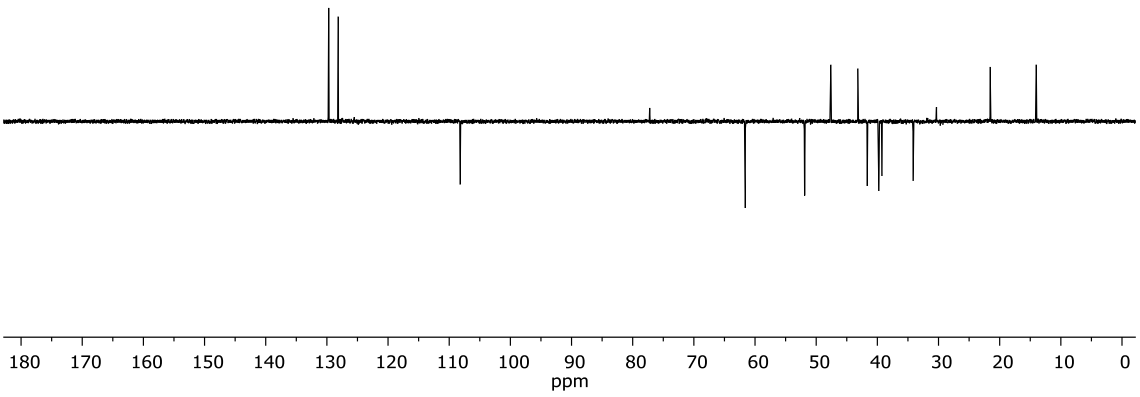

^1H NMR (300 MHz, $CDCl_3$)

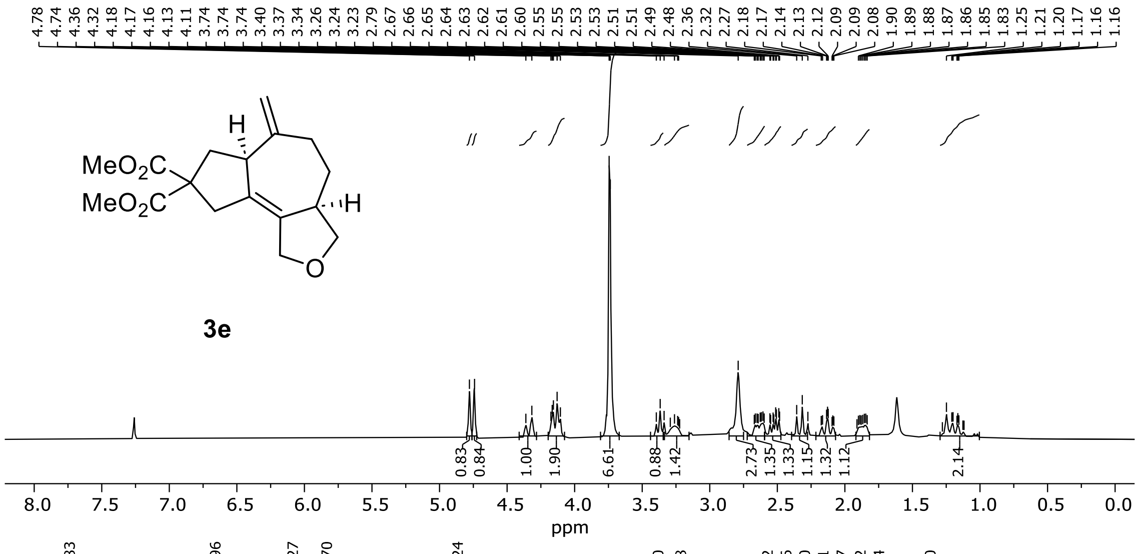

^{13}C NMR (75 MHz, $CDCl_3$)

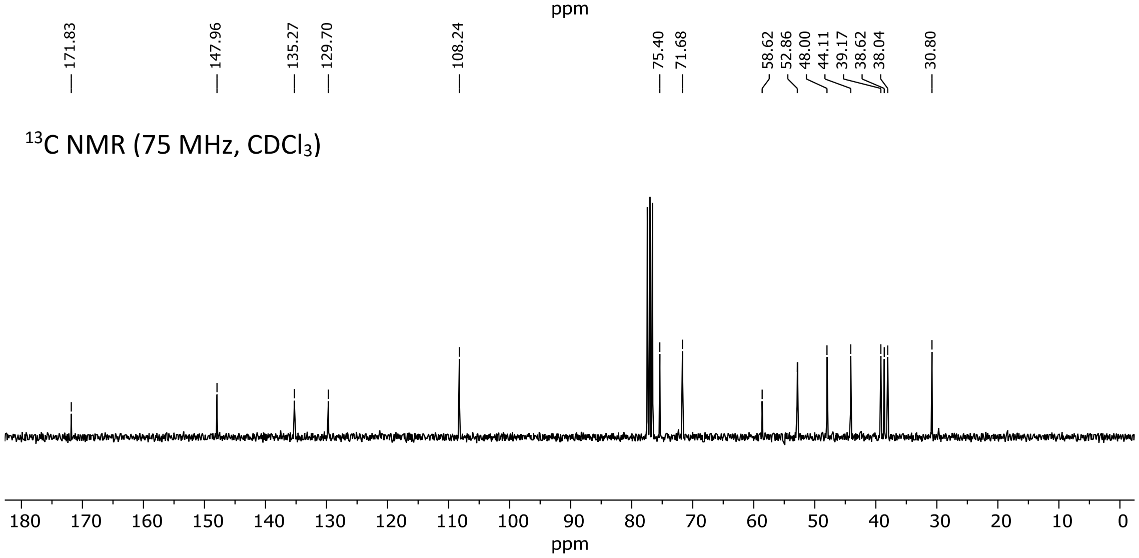

DEPT (75 MHz, $CDCl_3$)

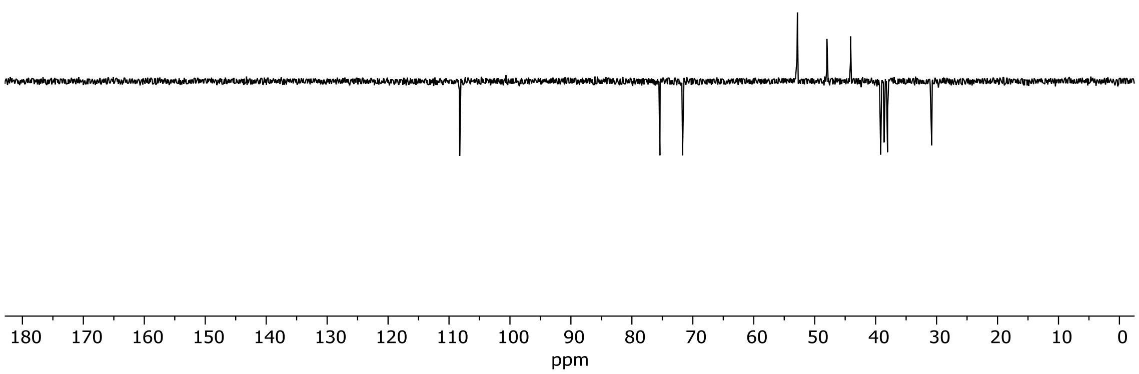

^1H NMR (300 MHz, $CDCl_3$)

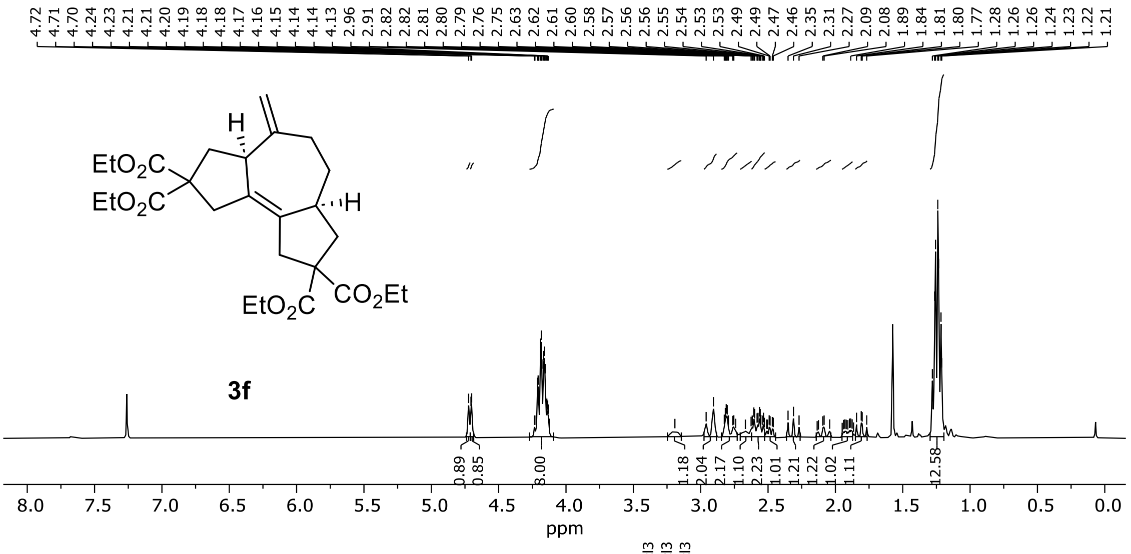

^{13}C NMR (75 MHz, $CDCl_3$)

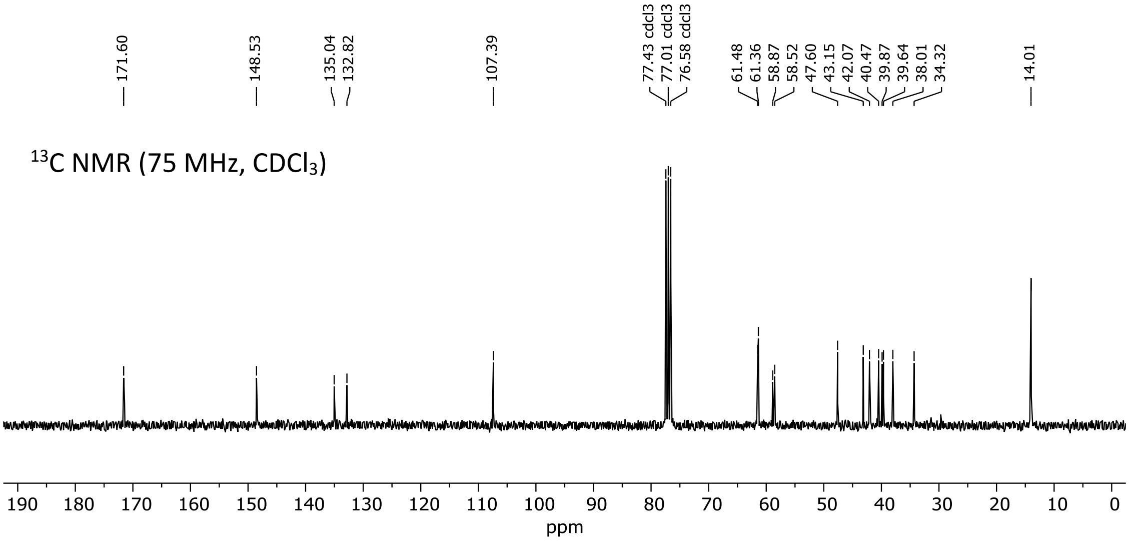

DEPT (75 MHz, $CDCl_3$)

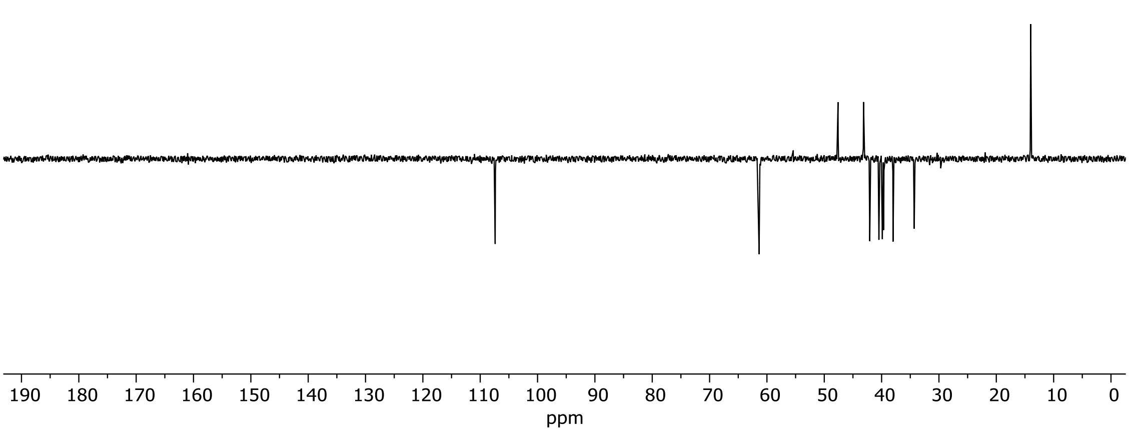

^{1}H NMR (300 MHz, $CDCl_3$)

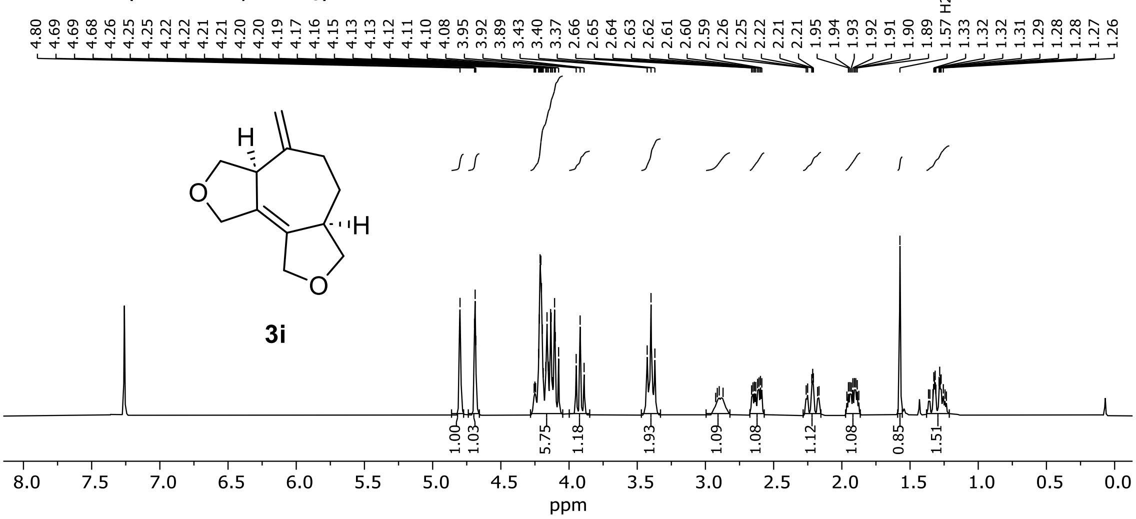

^{13}C NMR (75 MHz, $CDCl_3$)

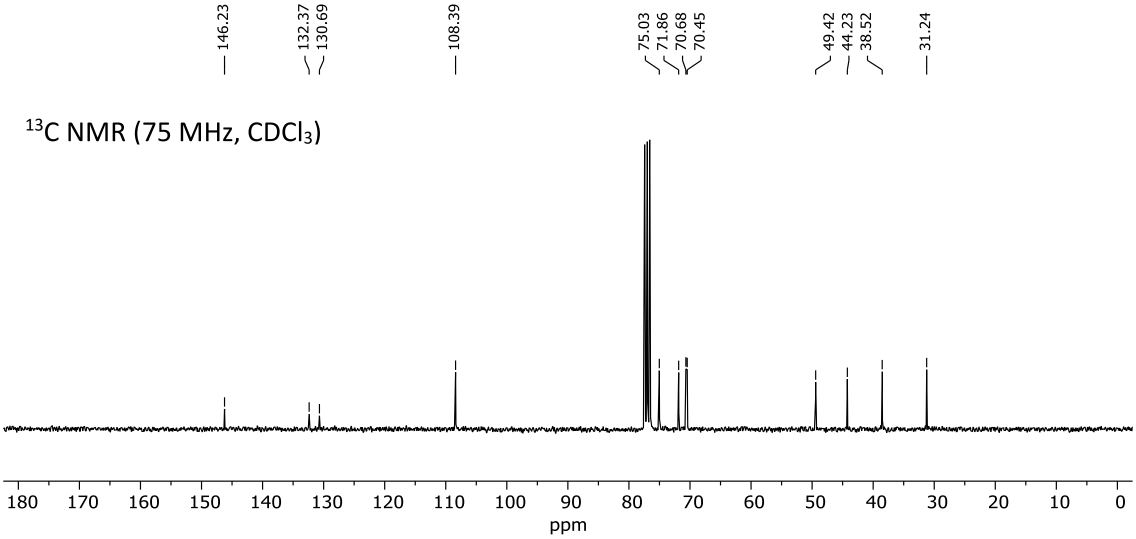

DEPT (75 MHz, $CDCl_3$)

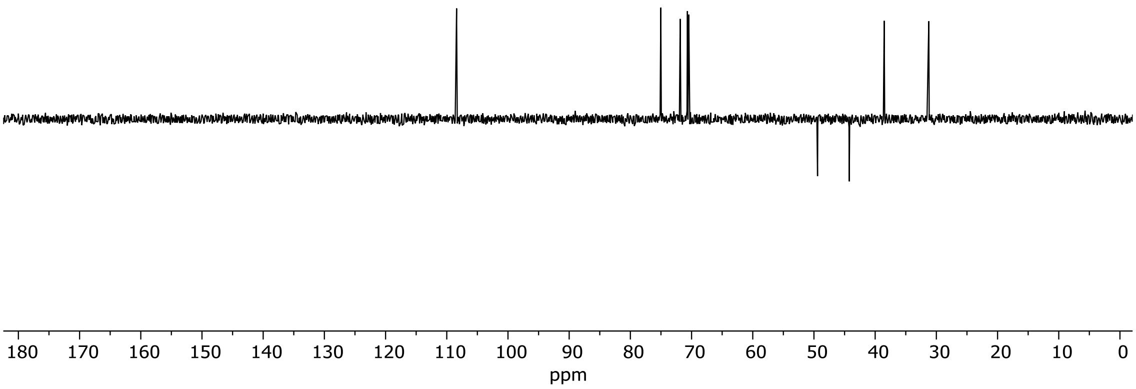

^{1}H NMR (300 MHz, $CDCl_3$)

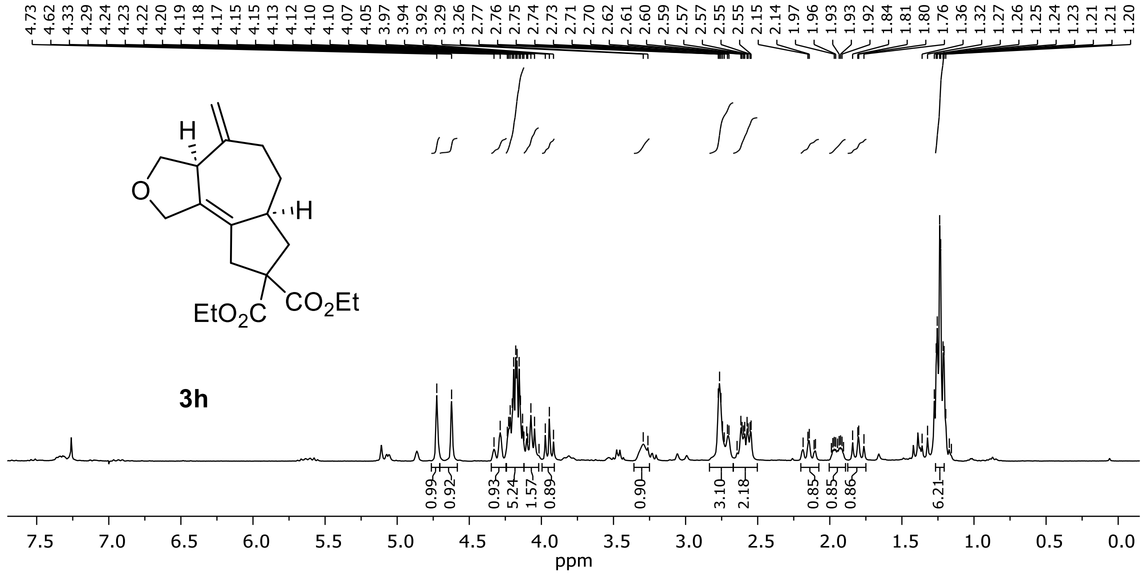

^{13}C NMR (75 MHz, $CDCl_3$)

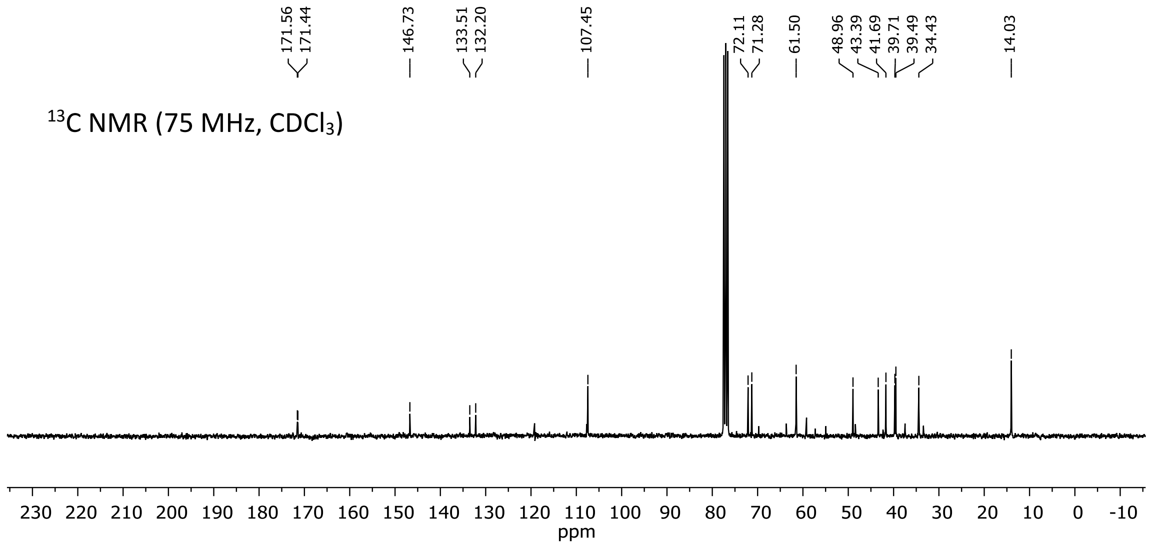

DEPT (75 MHz, $CDCl_3$)

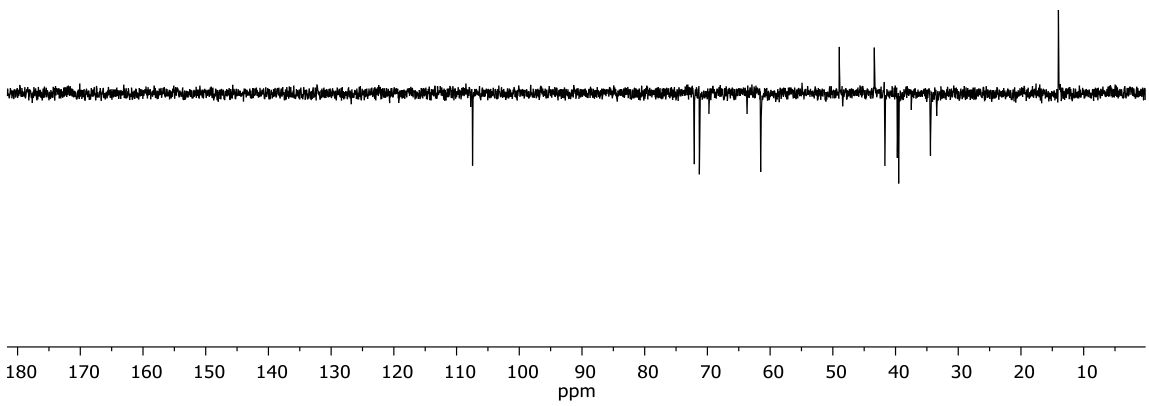

^{1}H NMR (300 MHz, $CDCl_3$)

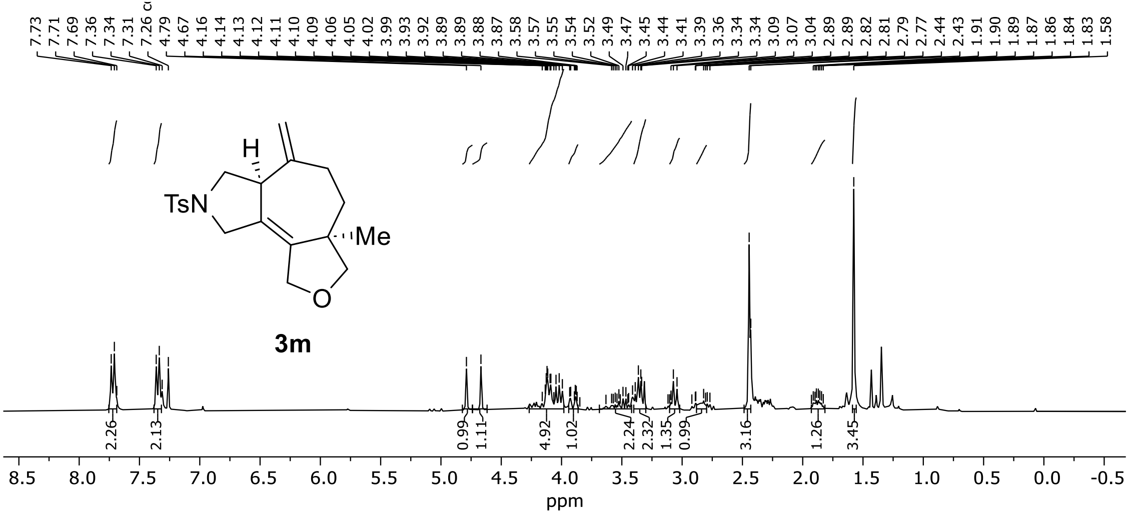

^{13}C NMR (75 MHz, $CDCl_3$)

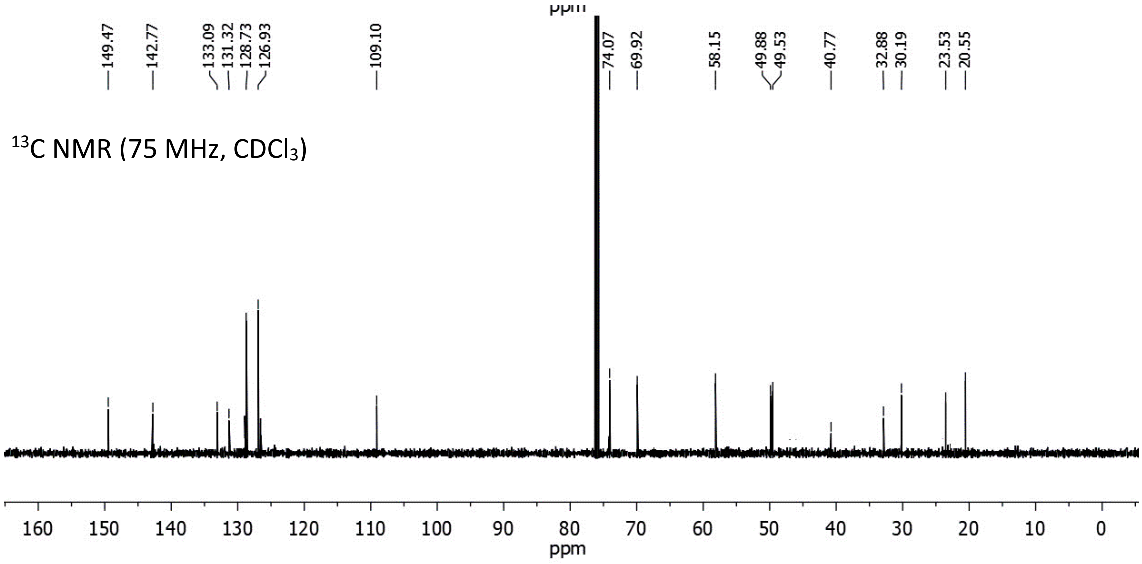

DEPT (75 MHz, $CDCl_3$)

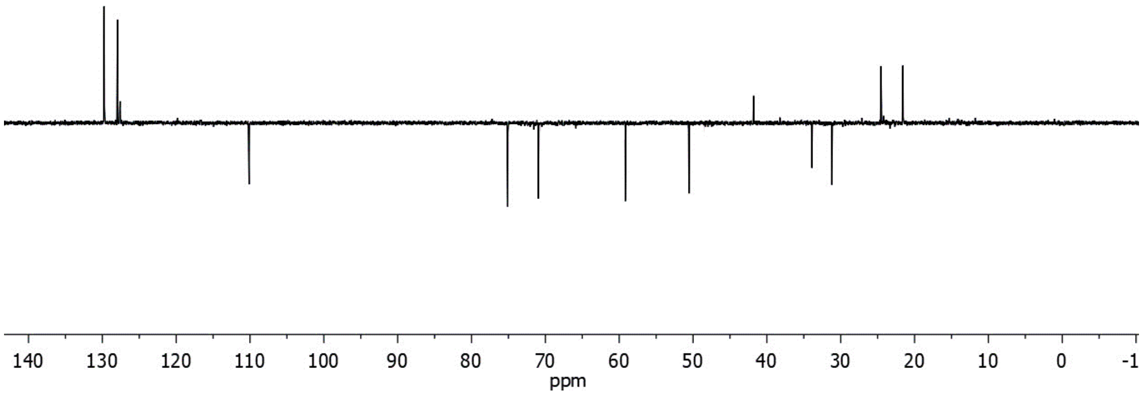

^1H NMR (500 MHz, $CDCl_3$)

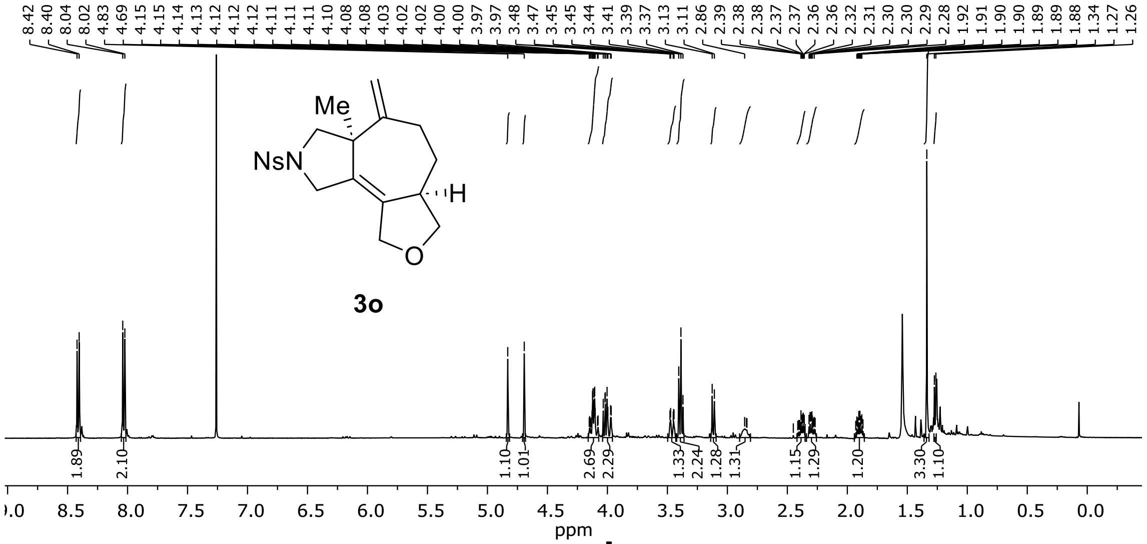

^{13}C NMR (126 MHz, $CDCl_3$)

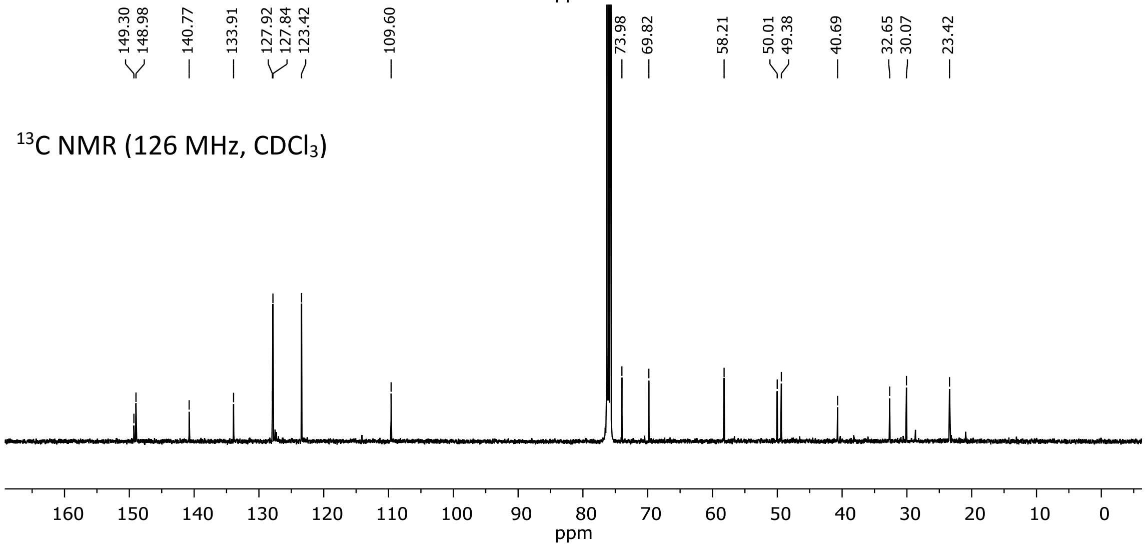

DEPT (75 MHz, $CDCl_3$)

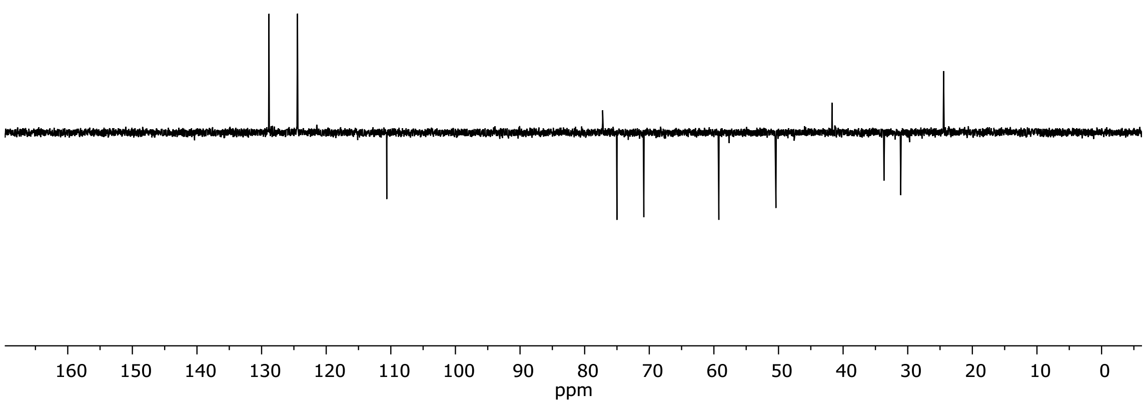

^{1}H NMR (300 MHz, $CDCl_3$)

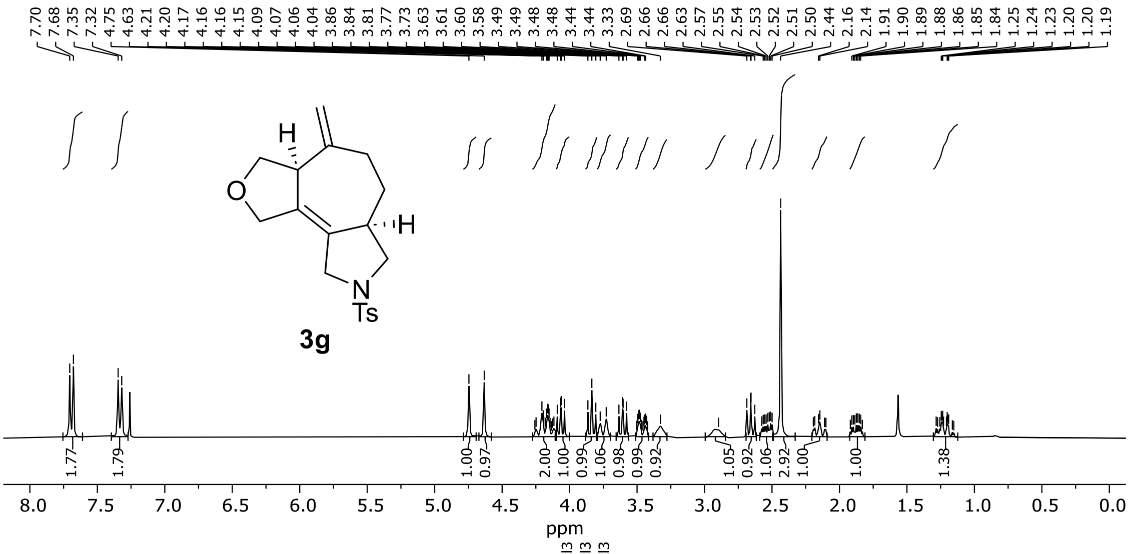

^{13}C NMR (75 MHz, $CDCl_3$)

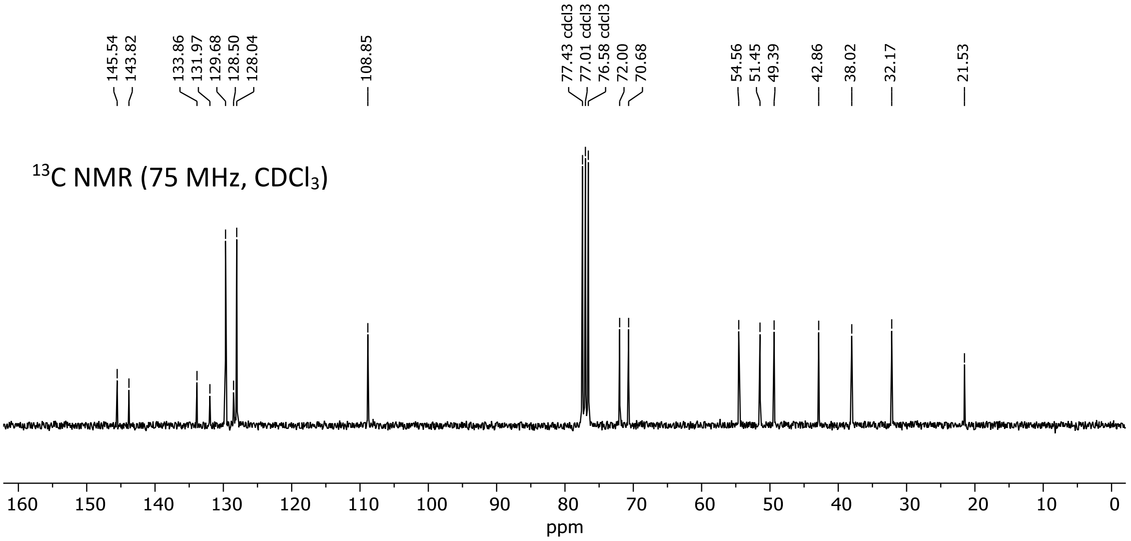

DEPT (75 MHz, $CDCl_3$)

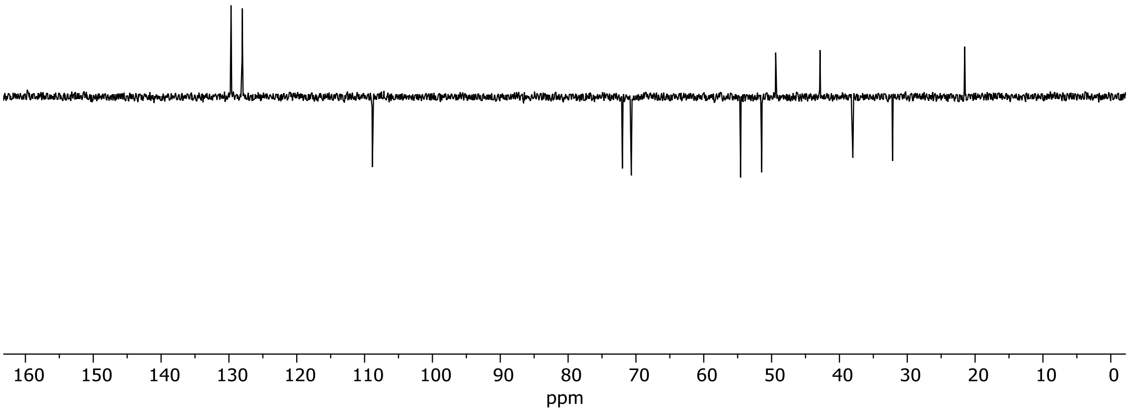

^{1}H NMR (300 MHz, $CDCl_3$)

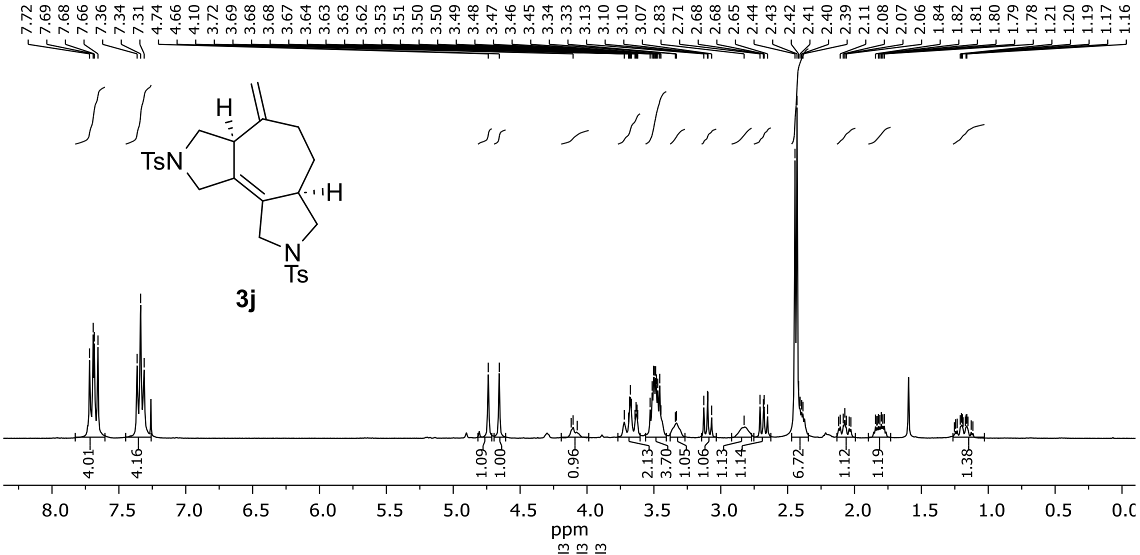

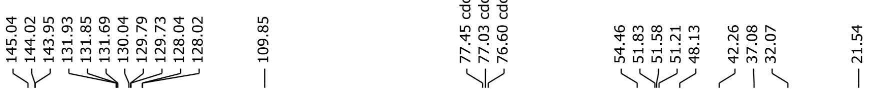

^{13}C NMR (75 MHz, $CDCl_3$)

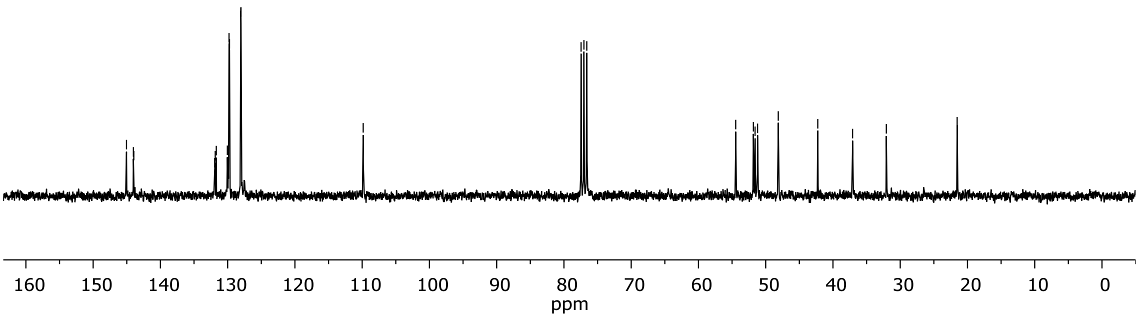

DEPT (75 MHz, $CDCl_3$)

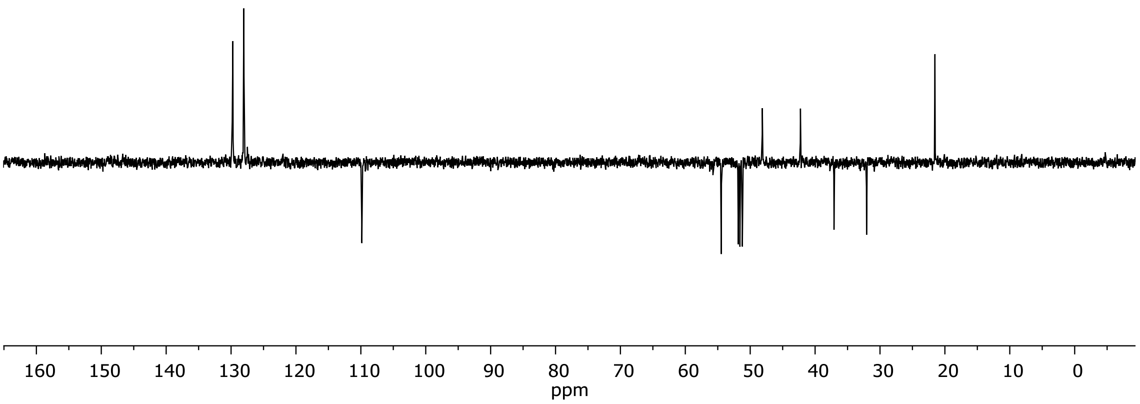

^{1}H NMR (300 MHz, $CDCl_3$)

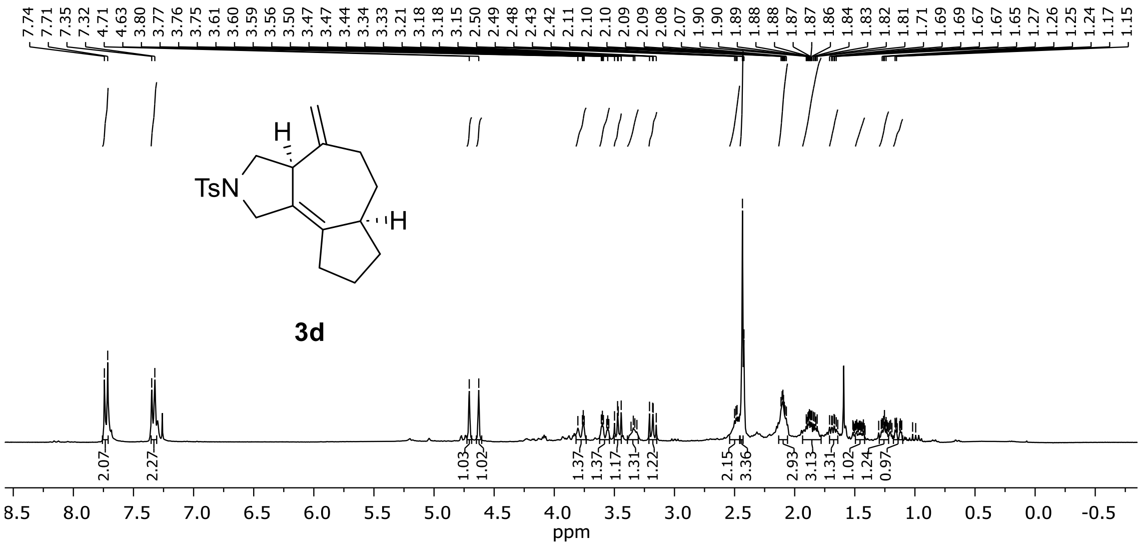

3d

^{13}C NMR (75 MHz, $CDCl_3$)

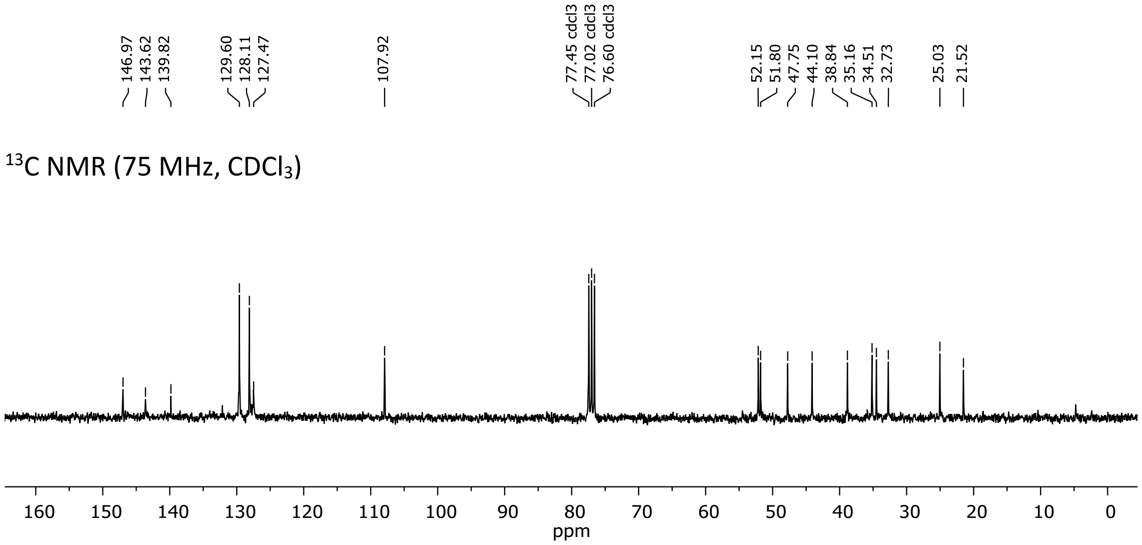

DEPT (75 MHz, $CDCl_3$)

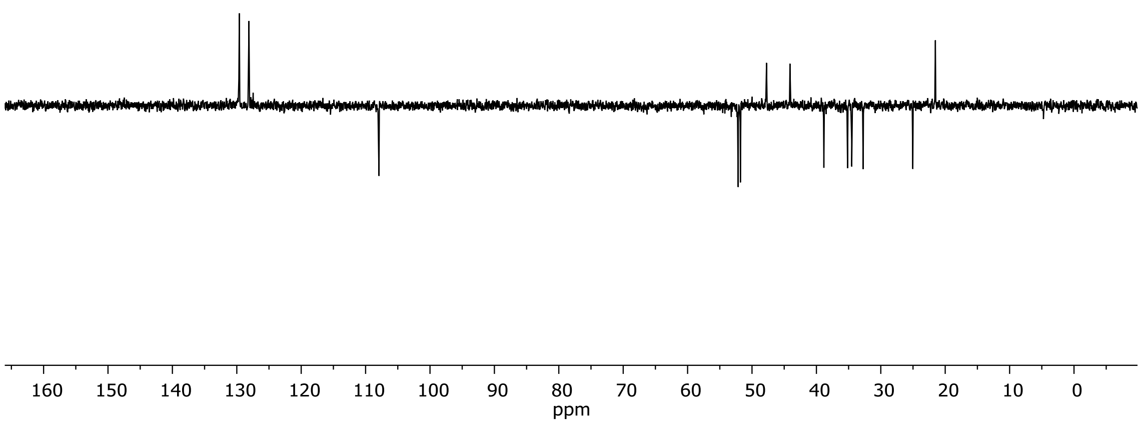

^{1}H NMR (300 MHz, $CDCl_3$)

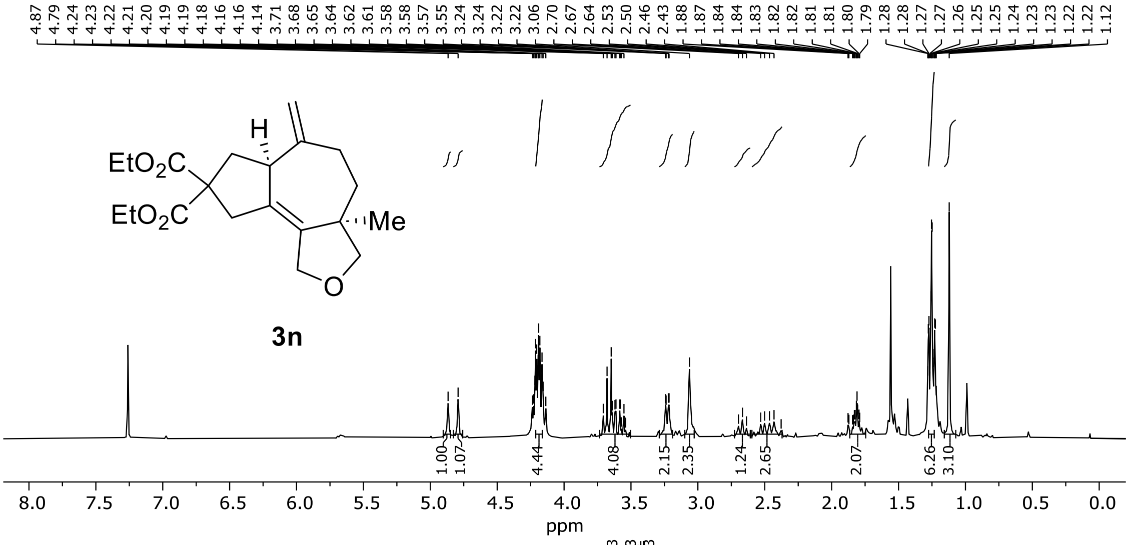

^{13}C NMR (75 MHz, $CDCl_3$)

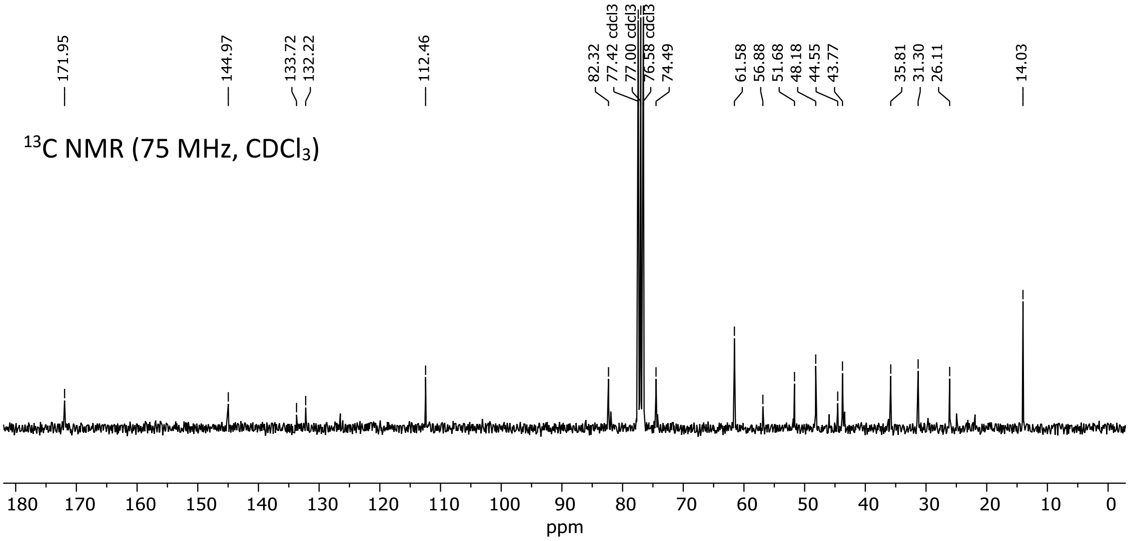

DEPT (75 MHz, $CDCl_3$)

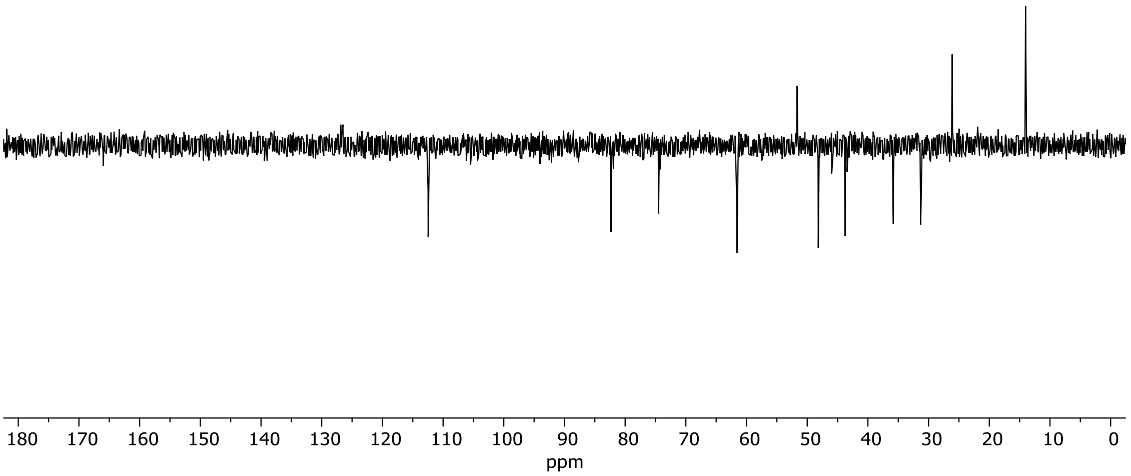

^{1}H NMR (300 MHz, $CDCl_3$)

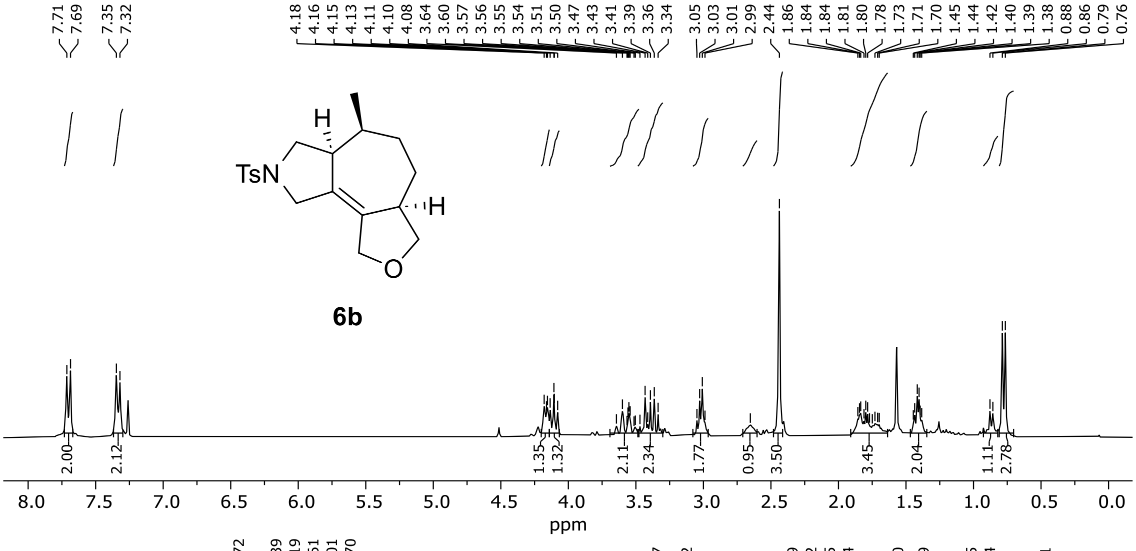

6b

^{13}C NMR (75 MHz, $CDCl_3$)

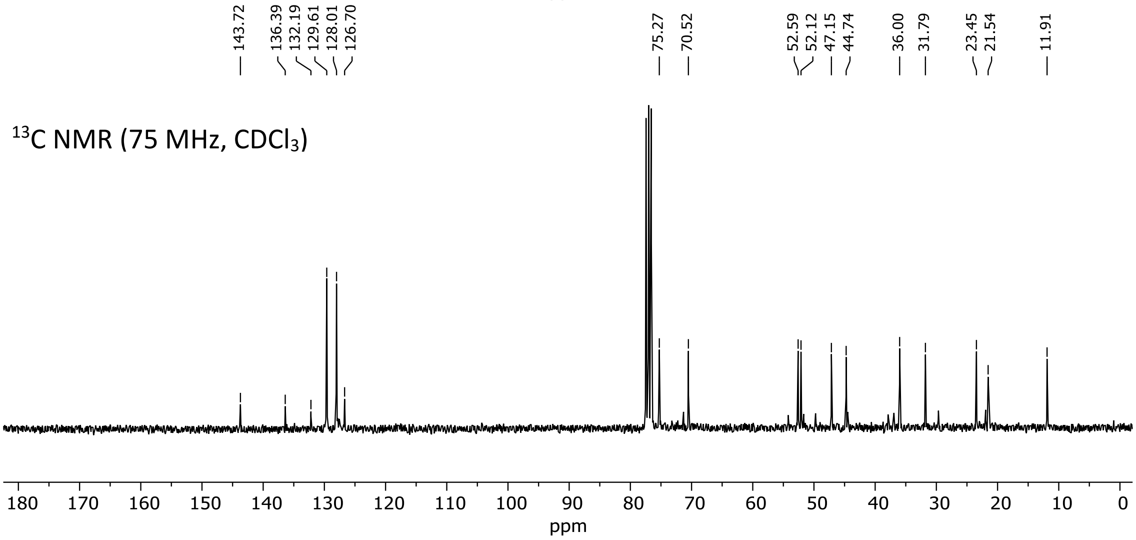

DEPT (75 MHz, $CDCl_3$)

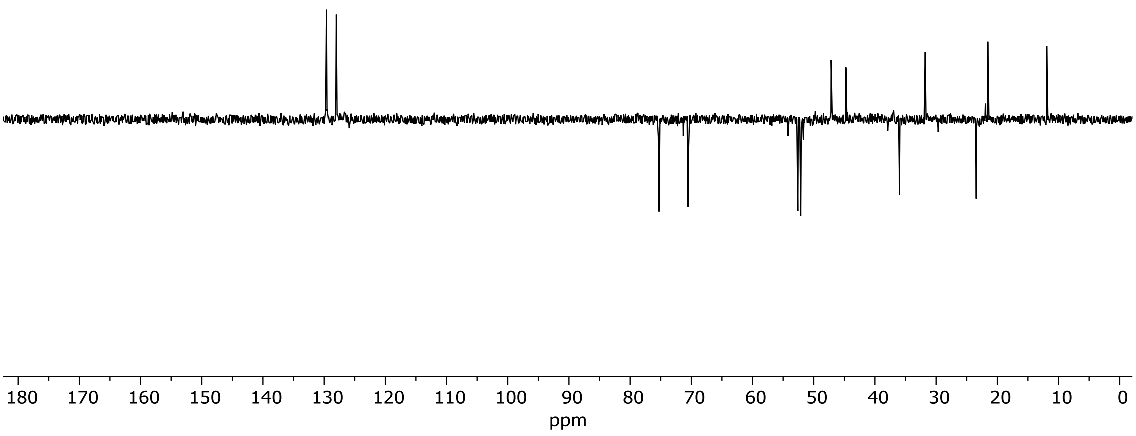

^1H NMR (300 MHz, $CDCl_3$)

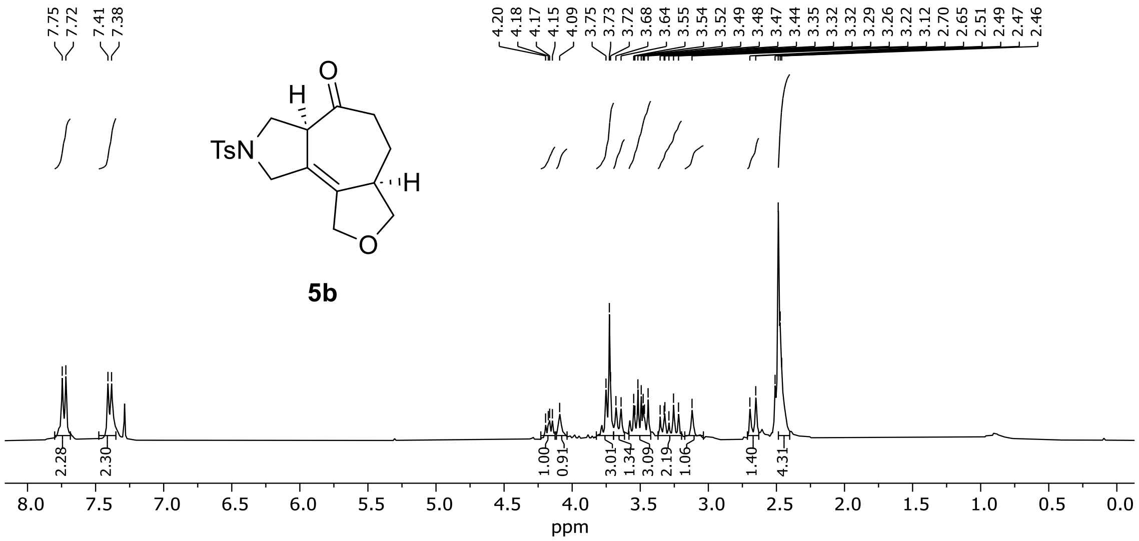

^{13}C NMR (75 MHz, $CDCl_3$)

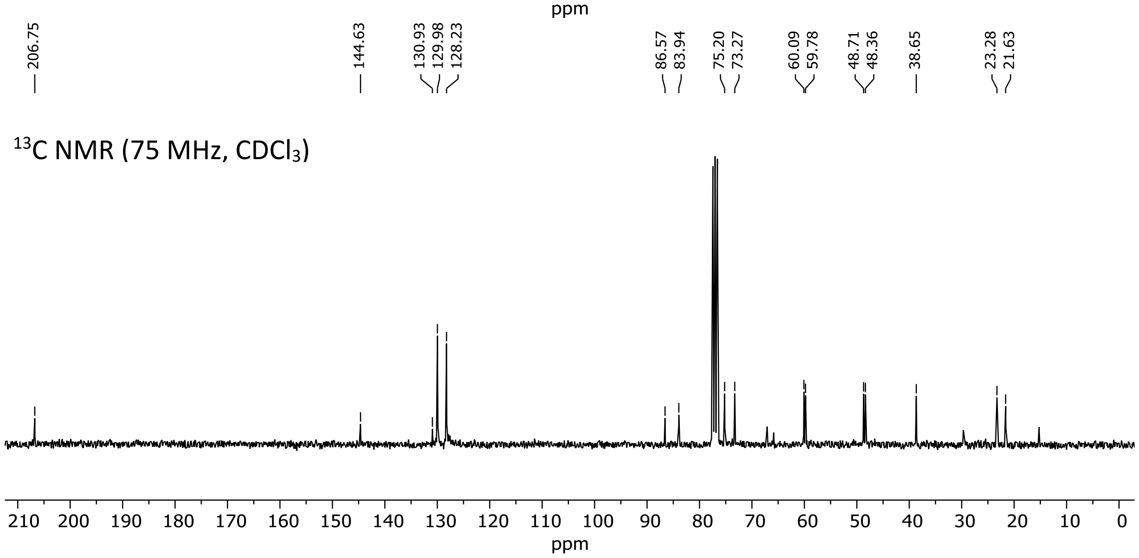

DEPT (75 MHz, $CDCl_3$)

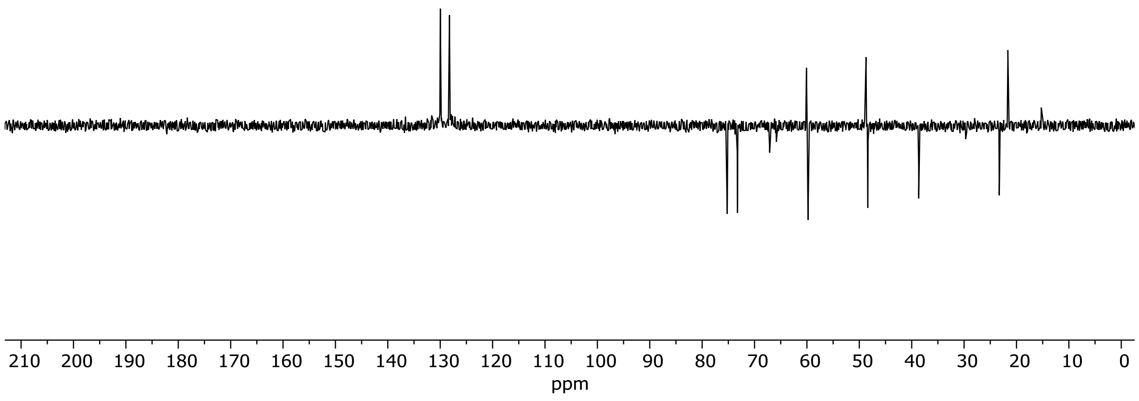

9. References

[1] Saya, L.; Bhargava, G.; Navarro, M. A.; Gulías, M.; López, F.; Fernández, I.; Castedo, L.; Mascareñas, J. L. *Angew. Chem. Int. Ed.* **2010**, 9886–9890.

[2] Araya, M.; Gulías, M.; Fernández, I.; Bhargava, G.; Castedo, L.; Mascareñas, J. L.; López, F.; *Chem. Eur. J.* **2014**, *20*, 10255–10259

[3] Gulías, M.; Durán, J.; López, F.; Castedo, L; Mascareñas, J. L.; *J. Am. Chem. Soc.* **2007**, *129*, 11026–11027.

[4] Bhargava, G.; Trillo, B.; Araya, M.; López, F.; Castedo, L.; Mascareñas, J. L. *Chem. Commun*. **2010**, *46*, 270-27.

[5] P. A. Evans, M. J. Dushnicky, D. Cho, J. Majhi, S. Choi, B. V. Pipaliya, P. A. Inglesby, M.-H. Baik, *Asian J. Org. Chem.* **2021**, *10*, 2174-2183.

[6] Gaussian 09, Revision D.01, M. J. Frisch, G. W. Trucks, H. B. Schlegel, G. E. Scuseria, M. A. Robb, J. R. Cheeseman, G. Scalmani, V. Barone, G. A. Petersson, H. Nakatsuji, X. Li, M. Caricato, A. Marenich, J. Bloino, B. G. Janesko, R. Gomperts, B. Mennucci, H. P. Hratchian, J. V. Ortiz, A. F. Izmaylov, J. L. Sonnenberg, D. Williams-Young, F. Ding, F. Lipparini, F. Egidi, J. Goings, B. Peng, A. Petrone, T. Henderson, D. Ranasinghe, V. G. Zakrzewski, J. Gao, N. Rega, G. Zheng, W. Liang, M. Hada, M. Ehara, K. Toyota, R. Fukuda, J. Hasegawa, M. Ishida, T. Nakajima, Y. Honda, O. Kitao, H. Nakai, T. Vreven, K. Throssell, J. A. Montgomery, Jr., J. E. Peralta, F. Ogliaro, M. Bearpark, J. J. Heyd, E. Brothers, K. N. Kudin, V. N. Staroverov, T. Keith, R. Kobayashi, J. Normand, K. Raghavachari, A. Rendell, J. C. Burant, S. S. Iyengar, J. Tomasi, M. Cossi, J. M. Millam, M. Klene, C. Adamo, R. Cammi, J. W. Ochterski, R. L. Martin, K. Morokuma, O. Farkas, J. B. Foresman, and D. J. Fox, Gaussian, Inc., Wallingford CT, 2009.

[7] (a) Becke, A. D. *J. Chem. Phys*. **1993**, *98*, 5648; (b) Lee, C.; Yang, W.; Parr, R. G. *Phys. Rev. B* **1998**, *37*, 785; (c) Vosko, S. H.; Wilk, L.; Nusair, M. *Can. J. Phys*. **1980**, *58*, 1200.

[8] Grimme, S.; Antony, J.; Ehrlich, S.; Krieg, H. *J. Chem. Phys*. **2010**, *132*, 154104.

[9] Weigend, F.; Ahlrichs, R. *Phys. Chem. Chem. Phys*. **2005**, *7*, 3297

[10] (a) Miertuš, S.; Scrocco, E.; Tomasi, J. *Chem. Phys*. **1981**, *55*, 117; (b) Pascual-Ahuir, J. L.; Silla, E.; Tuñón, I. *J. Comput. Chem.* **1994**, *15*, 1127; (c) Barone, V.; Cossi, M. *J. Phys. Chem. A* **1998**, *102*, 1995.

[11] McIver, J. W.; Komornicki, A. K. *J. Am. Chem. Soc.* **1972**, *94*, 2625.

[12] González, C.; Schlegel, H. B. *J. Phys. Chem*. **1990**, *94*, 5523.

[13] Da Concepción, E.; Fernández, I.; Mascareñas, J. L.; López, F. *Angew. Chem. Int. Ed*. **2021**, *60*, 8182.
